# A systematic review on the evaluation of endocrine-disrupting chemicals as potential neurotoxins during zebrafish development

**DOI:** 10.3389/fendo.2026.1741250

**Published:** 2026-04-23

**Authors:** Asok K. Dasmahapatra, Chayan Dutta, Anitha Myla, Paul B. Tchounwou

**Affiliations:** 1 Department of BioMolecular Science, Environmental Toxicology Division, University of Mississippi, University, Oxford, MS, United States; 2 Department of Biology, Experimental Biophysics, Humboldt University, Berlin, Germany; 3 Mississippi State Department of Health, Jackson State University, Jackson, MS, United States; 4 Research Centers in Minority Institutions (RCMI) Center for Urban Health Disparities Research and Innovation, School of Computer, Mathematical, and Natural Sciences, Morgan State University, Baltimore, MD, United States

**Keywords:** developmental neurotoxicity, endocrine disruptors, epigenetics, hypothalamus-pituitary-thyroid axis, neurobehavior, neurotransmitters, zebrafish

## Abstract

Endocrine-disrupting chemicals (EDCs) are well known for their ability to interfere with endocrine function; however, growing evidence indicates that they can also cause profound neurotoxic effects. These substances can affect both the central and peripheral nervous systems of aquatic organisms, including fish, and can disrupt neurobehavioral development, posing a potential threat to aquatic species. In this systematic review, we examine the neurotoxic effects of EDCs on zebrafish (*Danio rerio*), a widely utilized model organism for investigating vertebrate nervous system development. A comprehensive bibliographic search was conducted in PubMed using the terms “developmental neurotoxicity” and “fish,” which yielded 603 articles. The search was refined by incorporating the terms “endocrine disruptors” and “zebrafish,” resulting in 15 relevant studies. Of these, three were excluded—two due to irrelevance to the topic and one for being a review on strobilurin, a fungicide. The remaining 12 studies provided insights into the neurotoxic effects of 14 chemicals, including 6-OH-BDE-47 (2,2´,4,4´-tetrabromodiphenyl ether), atrazine, BDE-209, bisphenol (BPA), bisphenol S (BPS), Cd, estrone, OBS, Pb, tributyltin (TBT), tris(2-chloroethyl) phosphate (TCEP), tris(1,3-dichloro-2-propyl) phosphate (TDCPP), Thifluzamide (THM), and Ti. These compounds, encompassing biocides, heavy metals, hormones, brominated compounds, plastic components, and flame retardants, were identified as potential EDCs that disrupt nervous system development and behavior in zebrafish during embryonic, larval, and adult stages. Studies have also investigated co-exposure effects of binary mixtures like BDE-209 with Pb, BPA with Ti, and TBT with Cd. We identified significant neurodevelopmental endpoints, including alterations in thyroid/sex steroid hormone levels; neurotransmitter contents, including dopamine, serotonin, and GABA; AChE activity; locomotor behavior; and expression of sensitive genes and proteins. Notably, these neurotoxic effects were shown to have intergenerational/transgenerational/epigenetic consequences. Overall, this review provides comprehensive evidence of the neurotoxic potential of EDCs on zebrafish, emphasizing their relevance to vertebrate neurodevelopment and the potential implications for human health.

## Graphical Abstract

**Figure:**
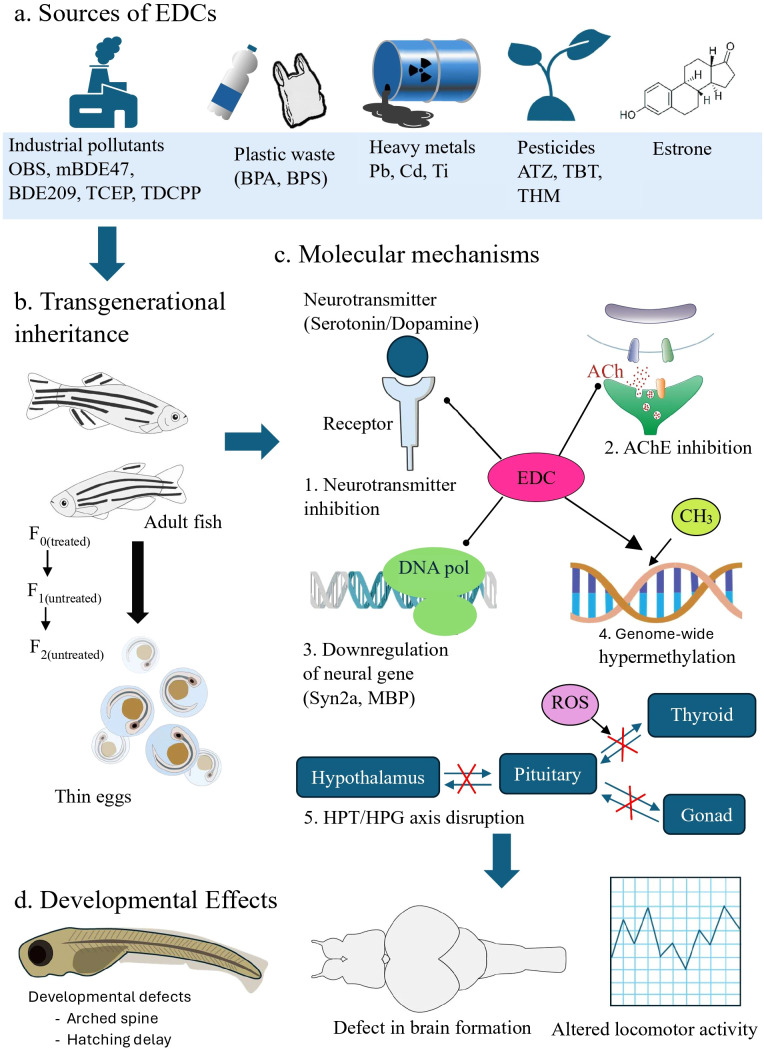

## 1 Introduction

Endocrine-disrupting chemicals (EDCs) are exogenous substances that interfere with the normal action of hormones and have been frequently implicated in adverse health effects (1–4). Developmental exposure to EDCs can induce irreversible structural and functional changes in tissues and organs, leading to detrimental health outcomes throughout the lifespan of an organism (5–7). The developmental period is recognized as the most sensitive stage to EDC exposure, with functional impairments often manifesting later in life (8, 9). It is widely acknowledged that aquatic ecosystems contain a mixture of pollutants that may act synergistically or additively to produce combined toxic effects in aquatic organisms (please see the graphical Abstract). The long-term exposure to these contaminants complicates the life cycle of the aquatic species and may result from the interactions among pollutants and their physicochemical properties (10). Current risk assessment approaches remain inadequate for evaluating the combined and transgenerational toxicity of environmentally relevant mixtures of contaminants. Although the health risks associated with environmental pollution—such as that arising from waste generated by electrical and electronic equipment (e-waste), persistent organic pollutants, and heavy metals—are well documented (11–13), research on the neurotoxic potential of these chemicals, particularly during development, remains limited. The Developmental Origins of Health and Disease paradigm suggests that early life exposure to environmental stressors can lead to genetic, epigenetic, or functional alterations associated with chronic diseases later (14, 15). Indeed, developmental exposure to toxicants has been linked to immune system dysfunction, obesity, metabolic syndrome, altered neurodevelopment, neurological deficits, and cancer (16).

The zebrafish was first identified as a genetically tractable organism in the 1980s (17). Owing to its ease of husbandry, high fecundity, sequenced genome, and conserved metabolic and signaling pathways, zebrafish have become widely used in toxicological and biomedical research (17–20). Furthermore, their utility in integrating genetic, cellular, and whole-organism endpoints provides a powerful approach for assessing the biological effects of toxicants (21–25).

In this study, we have evaluated the developmental origin of EDC-induced neurotoxicity using zebrafish as a model organism. Our systematic review focuses on the hypothesis that developmental exposure to EDCs induced neurological alterations observable later in life, including changes in body and brain size, neurobehavior, brain histopathology, neurotransmitter levels, acetylcholinesterase enzyme (AChE) activity, hypothalamus-pituitary-gonad (HPG) and hypothalamus-pituitary-thyroid (HPT) axis function, hormonal status, reproduction, and transgenerational effects, all of which have significant implications for human health.

## 2 Materials and methods

**Figure 1:**
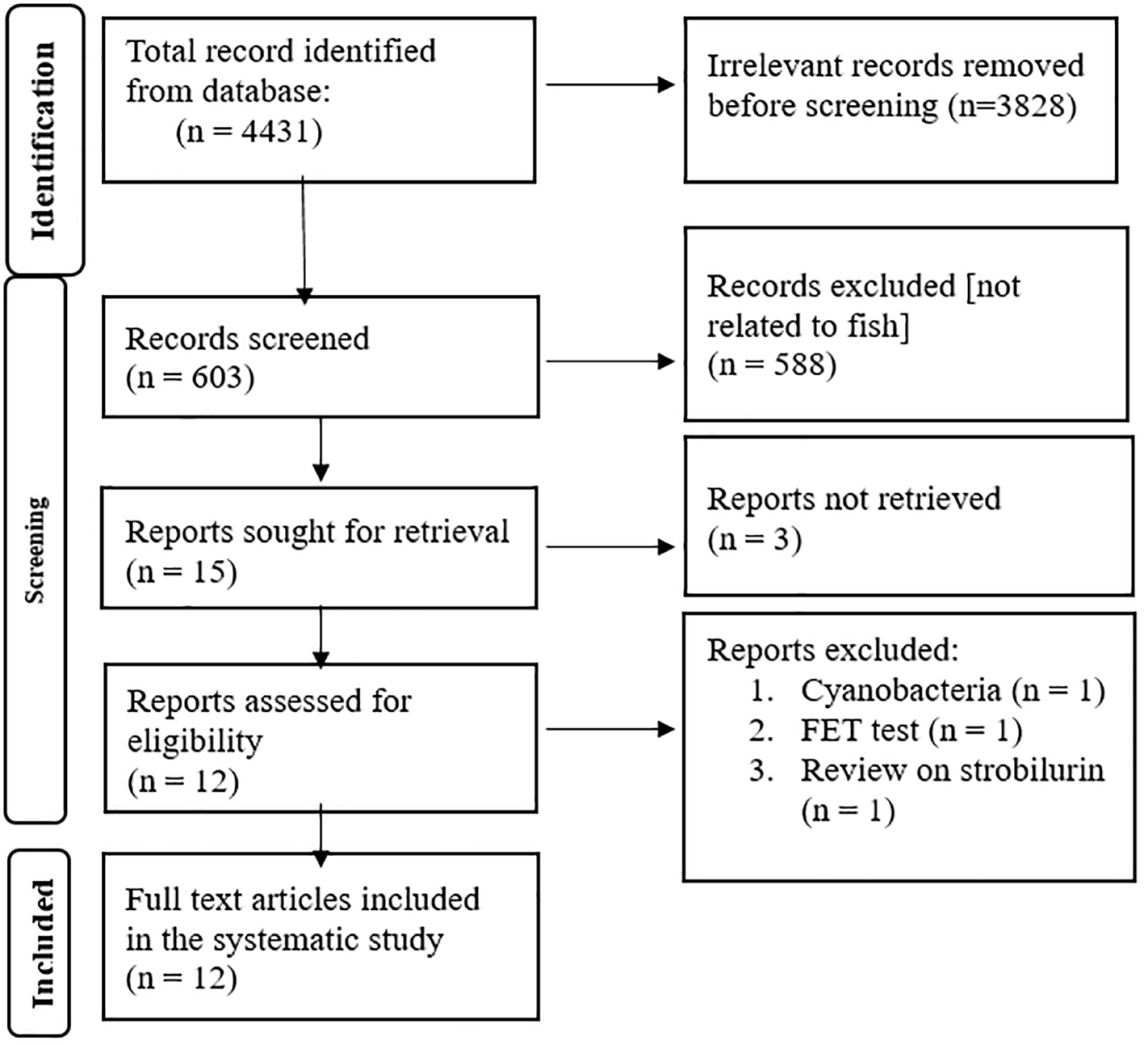
PRISMA flow diagram of the literature search.

Based on Preferred Reporting Items for Systematic Reviews and Meta-Analyses (PRISMA) (26), we conducted a comprehensive literature search of English-language journal articles to identify studies investigating the neurotoxic effects of EDCs during zebrafish development, with a special focus on the impacts at the molecular level (**Figure 1**). The electronic search was performed in PubMed (http://www.ncbi.nlm.nih.gov/pubmed) on 23 November 2024, using the search terms “developmental neurotoxicity” and “fish,” which yielded 603 articles. The search was further refined by including the terms “endocrine disruptors” and “zebrafish,” resulting in the selection of 15 relevant articles for detailed review. Among these 15 articles, three were determined to be unrelated to the study objectives and were excluded (one with the compounds found in cyanobacteria, another is a method paper related to the Fish Embryo Toxicity Test, OECD TG 236, and the third paper is a review article on strobilurin) from further analysis (please see Flow diagram). Ultimately, 12 articles were included, from which we identified 14 chemicals (**Table 1**) representing various classes—biocides including fungicides and herbicides, heavy metals, hormones, flame retardants, plastic components, and brominated compounds. These chemicals were recognized as potential EDCs capable of disrupting the nervous system and altering zebrafish behavior during development. Several key neurodevelopmental endpoints and genes were found to be dysregulated by these EDCs. Detailed information on the neurodevelopmental disorders associated with the 14 chemicals is provided in **Supplementary Table (ST-1)**, which has been deposited in Figshare (www.figshare.com).

**Table 1:** Chemical structure of EDCs with potential neurotoxicity in zebrafish.

Serial number	Common name	IUPAC name	Formula	Structure
1	Atrazine	2-chloro-4-ethylamino-6-isopropylamino-s-triazine	C_8_H_14_ClN_5_	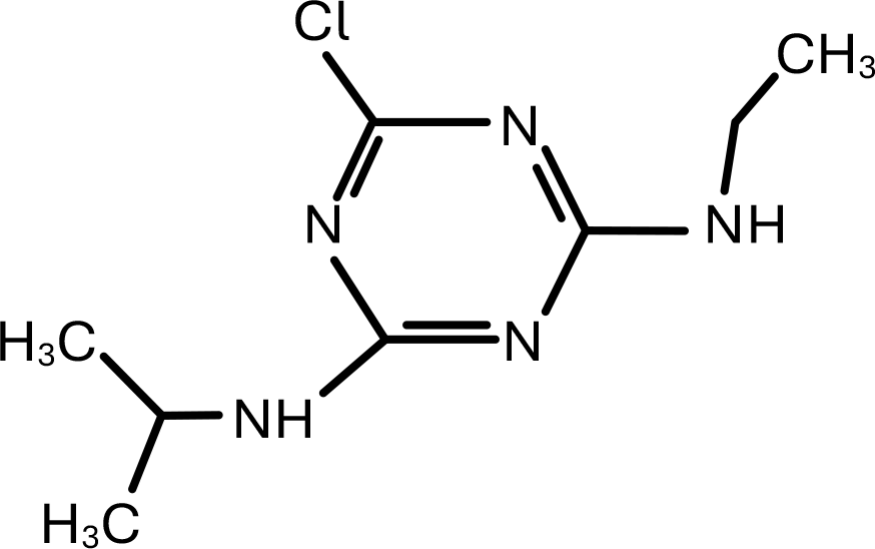 .
2	mBDE-47	6-hydroxy-2,2′,4,4′-Tetrabromodiphenyl ether	C_12_H_6_Br_4_O	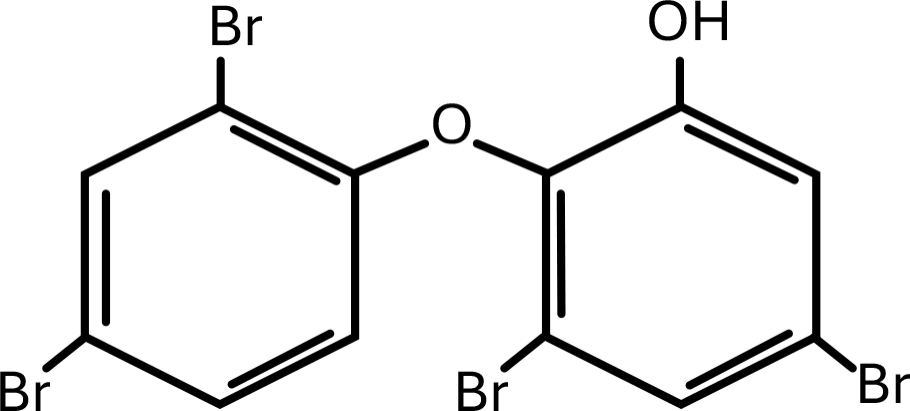 .
3	BDE-209	1,2,3,4,5-pentabromo-6-(2,3,4,5,6-pentabromophenoxy)benzene	C_12_Br_10_O	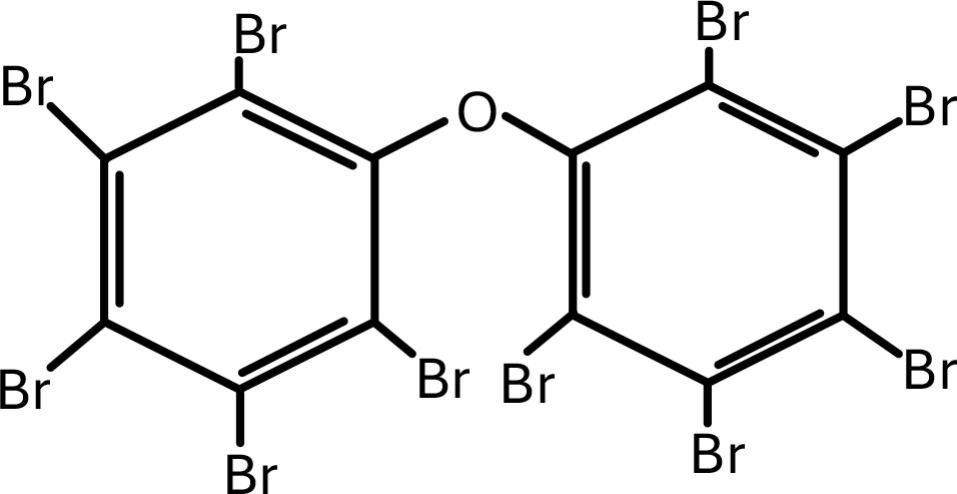 .
4	Bisphenol-A	4,4’-(propane-2,2-diyl)diphenol	C_15_H_16_O_2_	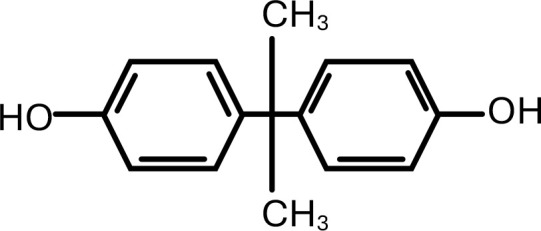 .
5	Bisphenol-S	4,4’-Sulfonyldiphenol	C_12_H_10_O_4_S	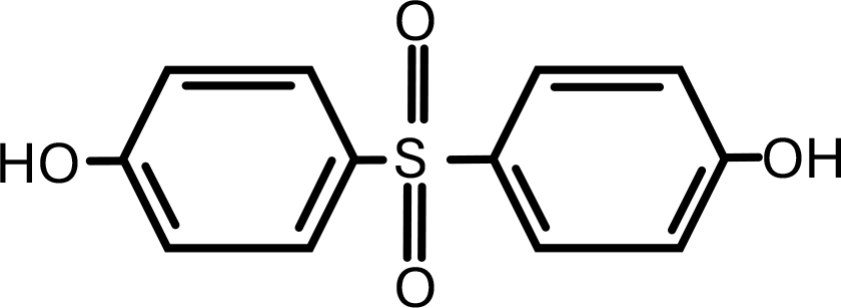 .
6	Cadmium	Cadmium Chloride	CdCl_2_	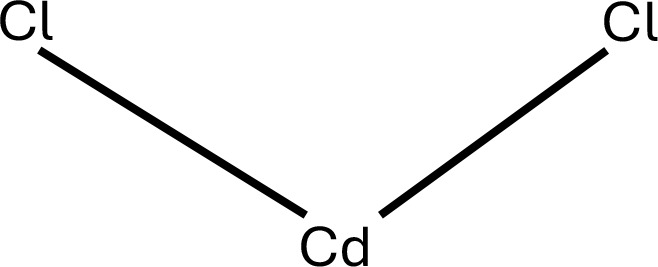 .
7	Estrone	(8R,9S,13S,14S)-3-hydroxy-13-methyl-7,8,9,11,12,14,15,16-octahydro-6H-cyclopenta[a]phenanthren-17-one	C_18_H_22_O_2_	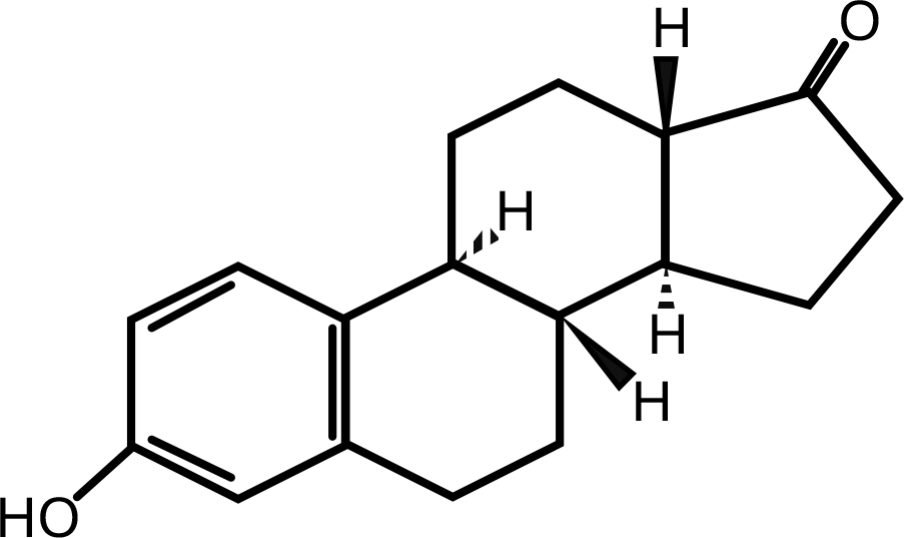 .
8	OBS	sodium;4-[1,1,1,4,5,5,5-heptafluoro-3-(1,1,1,2,3,3,3-heptafluoropropan-2-yl)-4-(trifluoromethyl)pent-2-en-2-yl]oxybenzenesulfonate	C_9_F_17_OC_6_H_4_SO_3_Na	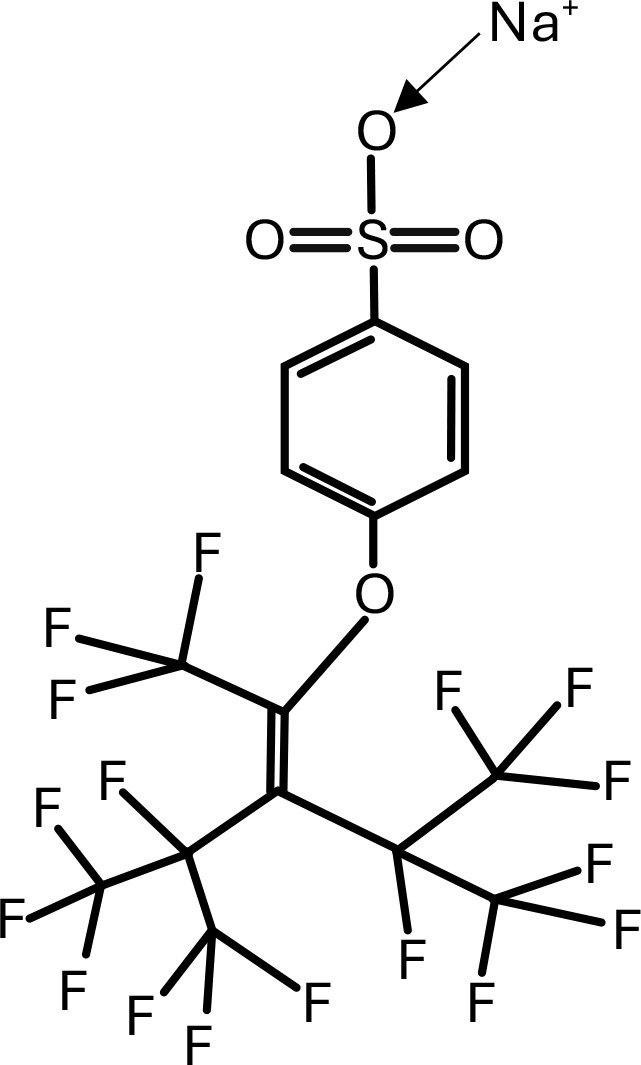 .
9	Lead	Lead acetate trihydrate	(C_2_H_3_O_2_)_2_ Pb. 3H_2_O	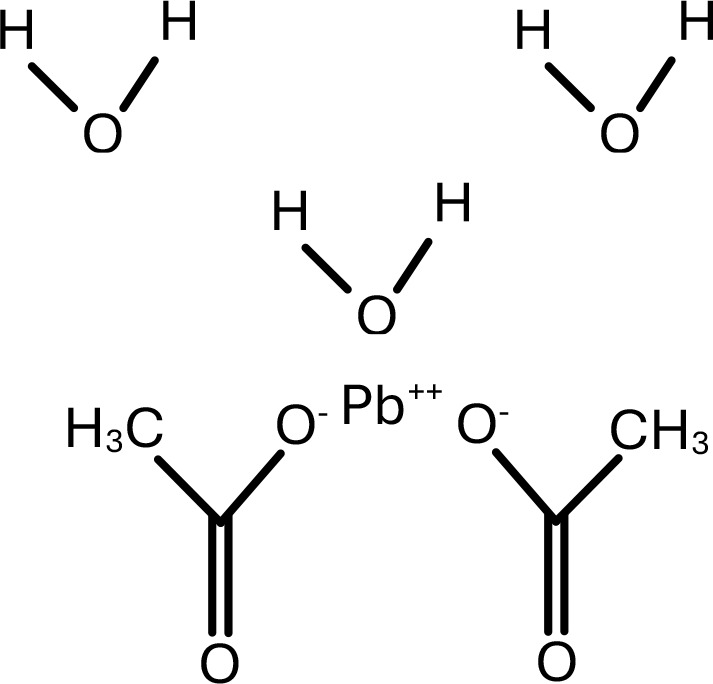 .
10	TBT	Tributyltin	C_12_H_27_Sn	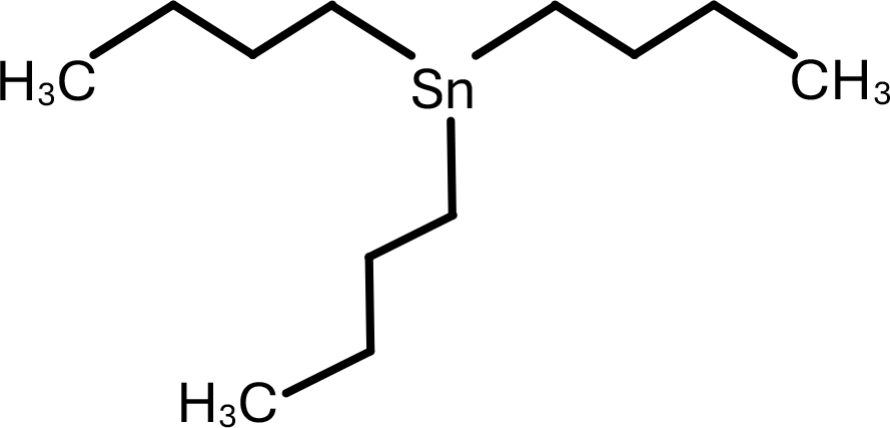 .
11	TCEP	tris(2-chloroethyl) phosphate	C_6_H_12_Cl_3_O_4_P	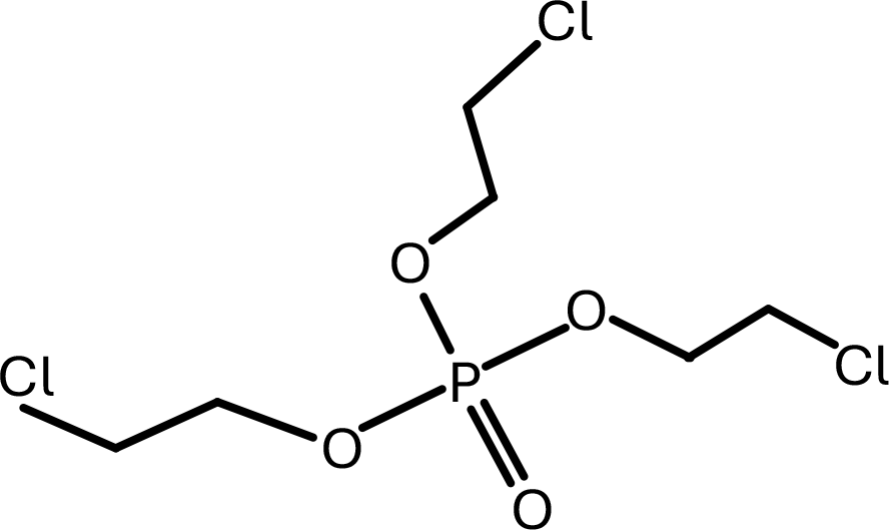 .
12	TDCPP	Tris(1,3-dichloro-2-propyl) phosphate	C_9_H_15_Cl_6_O_4_P	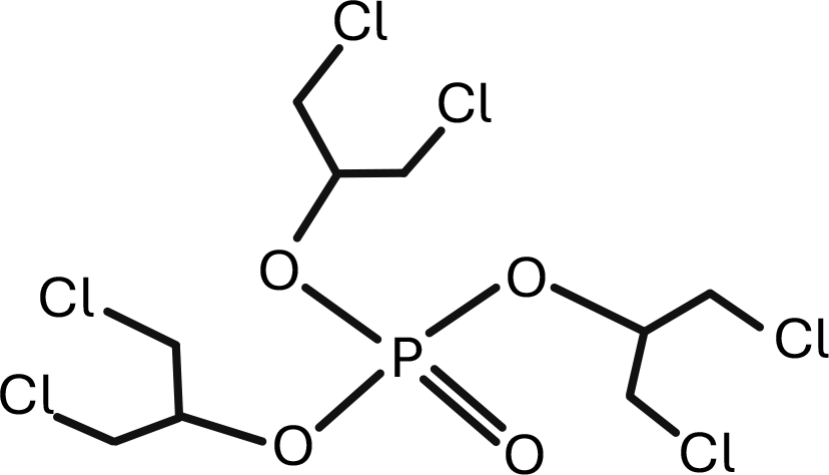 .
13	Thifluzamide	N-[2,6-dibromo-4-(trifluoromethoxy)phenyl]-2-methyl-4-(trifluoromethyl)-1,3-thiazole-5-carboxamide	C_13_H_6_Br_2_F_6_N_2_O_2_S	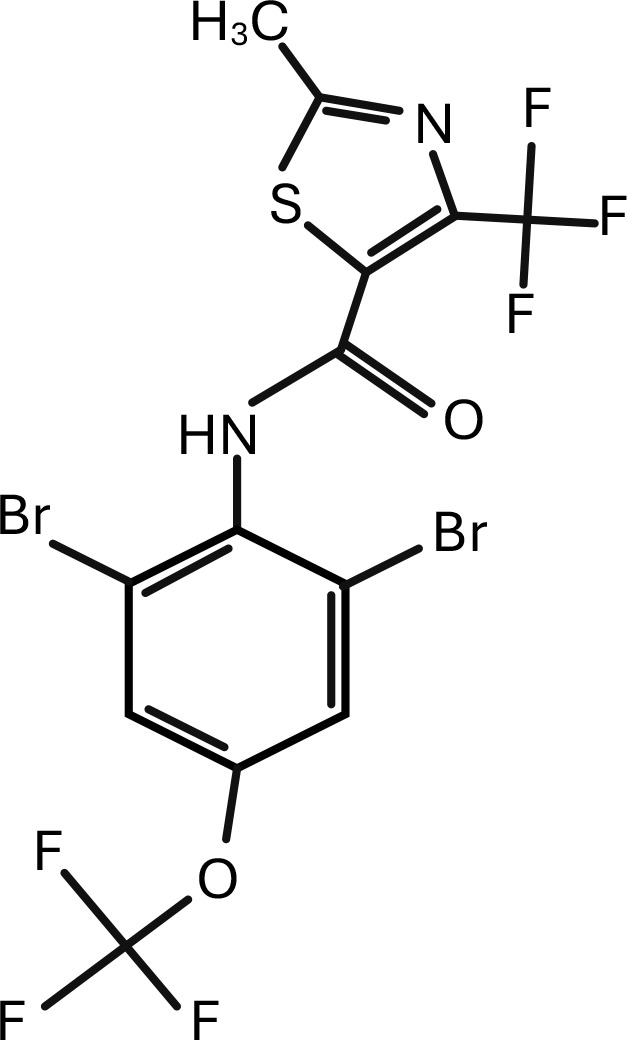 .
14	Titanium	Titanium dioxide	TiO_2_	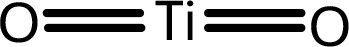 .

mBDE-47, 6-OH-bromodiphenyl ether.

OBS, sodium *p*-perfluorous nonenoxybenzene sulfonate.

TBT, tributyltin.

TCEP, tris(2-chloroethyl) phosphate.

TDCPP, tris(1,3-dichloro-2-propyl) phosphate.

The search of the articles on the PubMed database was made on 23 November 2024.

Inclusion criteria: (1) developmental neurotoxicity, (2) endocrine disruption, (3) zebrafish.

Exclusion criteria: (1) articles published in a non-English language (2) irrelevant to the topic (3) review articles.

## 3 Results

A total of 12 articles consisting of 14 chemicals that studied the effects of EDCs on the nervous system of zebrafish during development were found (**Table 2A**). Despite wide variability in experimental design, maintenance, and strain of the zebrafish (**Table 2B**), the targeted endocrine systems are thyroid (ATZ, BDE-209, mBDE-47, BPA, BPS, Cd, OBS, TBT, TCEP, TDCPP, THM, TiO_2_) and steroid (BDE-209, E1, and Pb) hormone synthesis and pathways (**Tables 3**–**7**; **Supplementary Data Table ST-1**). Our study also indicated that, among 14 chemicals, seven chemicals were bought from US companies (AccuStandard, New Haven, CT, USA; Spectrum Laboratory Products, USA; Sigma-Aldrich, St. Louis, MO, USA), with the highest purity level available. Rests are either from China (Shanghai Futian Chemical Technology Co., Sinopharm Chemical Company Reagent Co. Ltd., Shanghai, China; Beijing Huarong Biological Hormone Plant) or Japan (Tokyo Chemical Industry Co. Ltd., Tokyo, Japan) (**Table 2B**).

**Table 2A:** List of authors studied the effect of EDC on neurotoxicity in zebrafish.

Serial number	Authors	chemicals	Developmental stage	Mode of exposure/additives	Concentration/dose	Durations	Evaluation
1	[Chen et al. (27)]	BDE-209 and Pb	Adults	Waterborne [[14L: 10D; 28 ± 0.5°C]	BDE-209 (1, 10, 100 µg/L), Pb (10 µg/L)	3 months (parental exposure)	F0 (adults), eggs (F1), larvae (5 dpf, F1)
2	[Guo et al. (28)]	BPA and TiO_2_	Adults [AB strain]	Waterborne [14L: 10D; 28 ± 0.5°C]	BPA (2 and 20 µg/L); TiO2 (100 µg/L)	4 months (parental exposure)	F0 adults, eggs (F1) and F1 larvae (10 dpf)
3	[Horzmann et al., (29)]	Atrazine	Embryos [AB wild type]	Waterborne [14L: 10D; 26–28°C]	0.3, 3, 30 µg/L	1–72 hpf	9, 12, and 14 months
4.	[Hu et al. (30)]	TCEP	Embryos [Tuebingen]	Waterborne [[14L: 10D; 28 ± 1°C]	0.2, 2. 20, and 200 µg/L	3–120 hpf	48, 72, 96, and 120 hpf
5	[Wang et al. (31)]	TDCPP	Adults [wild type AB strain]	Waterborne [14L: 10D; 28°C]	4, 20, and 100 µg/L	3 months	F0 adults, F1 eggs, F1 larvae (5 dpf)
6	[Li and Li (32)]	TBT and Cd	Adults (F0), F1 and F2 [AB strain]	Waterborne [14L: 10D; 27 ± 1°C]	100 ng/L TBT and 100 ng/L Cd	90 days (F0 parents); 5 months (F1 parents); 7 dpf (F1 larvae); 7 dpf (F2 larvae)	F0 (parents), F1 (eggs, larvae, and parents), and F2 (Eggs and larvae)
7	[Wang et al. (33)]	mBDE-47	Embryos, 5-D (AB line)	Waterborne [14L: 10D; 28°C]	1, 10, 50, and 100 nM [0.5018-50.18 µg/L)	4–96 hpf	22, 26, 30, 34, and 96 hpf
8.	(Wang et al. (34))	Atrazine	Embryos [AB wild type]	Waterborne [14L: 10D; 26–28°C]	0.3, 3, 30 µg/L	1–72 hpf	9 months
9.	[Wei et al. (35)]	BPS	Embryos [Tuebingen]	Waterborne [14L: 10D; 28 ± 0.5°C]	1, 10, and 100 µg/L	2 hpf–120 dpf	F0 adults. F1 as eggs and larvae
10 a.*	[Wu et al. (36)]	BPA	Embryos [AB wild type]	Waterborne [14L: 10D; 27–30°C]	1, 10, 100, 1000, and 10,000 nm BPA [0.23 μg/l-2.28 mg/L]	0–5 dpf and 4–5 dpf	24, 48, 72, 96, and 120 hpf
10.b*	[Wu et al. (36)]	Estrone	Embryos [AB wild type]	Waterborne [14L: 10D; 27–30°C]	0.01, 0.1, 1, 10, and 100 nm estrone (0.027–2.7 µg/L)	0–5 dpf and 4–5 dpf	24, 48, 72, 96, and 120 hpf
11	[Yang et al. (37)]	Thifluzamide	Embryos [AB strain]	Waterborne	0.19, 1.90, and 2.85 mg/L	4–6 days	4–6 days
12	[Zhao et al. (38)]	OBS	Embryos	Waterborne [14L: 10D; 26 ± 0.5°C]	3, 30, and 300 µg/L	2 hpf–21 days	F0 adults (180 dpf) and F1 larvae (7 dpf)

*Wu et al. (36) studied the effects of BPA and estrone on zebrafish embryos as individual compounds and included in one article (article number 10a and 10b).

**Table 2B:** Sources of chemicals and mode of application in zebrafish.

Name of the chemical	Source	IACUC	Condition of addition	Feeding	References
Atrazine [biocide]	CAS 1912-24–9 [technical grade; 98.1% purity]	Purdue University	Exposed 72 hpf; media containing ATZ did not change during exposure period	Adults: Mixture of brine shrimp, golden Pearls (500–800 µm), and Zeigler adult zebrafish food [fed twice daily; stocking density 5–10 fish/L] Larvae: Zeigler larval AP 100 powdered diet, paramecia, and brine shrimp [stocking density 50 larvae/L] Juveniles: Brine shrimp and Golden Pearls (300–500 µm; Artemia International) [stocking density 5–10 fish/L)	(Horzmann et al. (29)
Atrazine [biocide]	CAS 1912-24-9 (98.1% purity)	Purdue University	Exposed 72 hpf; media containing ATZ did not change during exposure period	Mixture of brine shrimp (*Artemia franciscana*; Artemia International LLC., Fairview, TX, USA), Zeigler adult zebrafish food (Zeigler Bros Inc., Gardners, PA, USA) [Adults fed twice daily]	(Wang et al. (34)
^4^BDE-209 (brominated compound)	Purity (> 95%); Tokyo Chemical industry Co. Ltd., Tokyo, Japan.	Exposure solution was renewed daily [the duration of exposure was 3 months]		Adults are fed twice daily with flake food and newly hatched *Artemia nauplii*. [stocking density 10 males and 10 females in 10 L media]	(Chen et al. (27)
Bisphenol A^1^ (plastic components)	Sigma-Aldrich (St. Louis, MO, USA)	Exposure solution renewed daily with 0.005% DMSO and treatment continued for 4 months.	National Institute for Food and Drug Control of China.	Adult fish fed twice daily with pellet food (Zeigler Bros Inc., Gardners, PA, USA) [stocking density 12 males and 20 females in 20 L exposure medium]	(Guo et al. (28)
Bisphenol A (plastic components)	CAS 80-05-7, Sigma-Aldrich, USA	Vehicle (0.01% acetone). Media prepared daily and renewed every day. (30 embryos/exposure solution)	Wayne State University	Adult fish fed twice daily with Aquatox fish diet flakes (Zeigler, PA, USA)	(Wu et al. (36)
Bisphenol S (plastic components)	CAS No. 80-09-1 (purity 99%) Sigma-Aldrich (Shanghai, China)	Two-thirds of the exposure solution renewed daily containing 0.002% DMSO. Exposure duration was 120 dpf	OECD TG 210 and OECD TG 230.	From 5–15 dpf fed with paramecium and onwards with *artemia nauplii* twice daily	(Wei et al. (35)
Cd ^2^ (metal)	Sigma-Aldrich (St. Louis, MO, USA)	Half of the solution was changed every day [exposure duration was 90 days]	Shandong University, Weihai, 264209, China.	Fed with freshly hatched brine shrimp (*Artemia salina*)	(Li and Li (32)
E1 (steroid hormone)	CAS 53-16-7, Spectrum Laboratory Products, USA.	Vehicle (0.01% acetone). Media prepared daily and renewed every day. (30 embryos/exposure solution)	Wayne State University	Adult fish fed twice daily with Aquatox fish diet flakes (Zeigler, PA, USA)	(Wu et al. (36)
mBDE-47 (brominated compound)	Accustandard (new Haven, CT, USA); Catalog No; HBDE-4005S-CN-0.2x	Duke University	Embryos were exposed from 4–96 hpf	Adults fed twice daily with hatched brine shrimp and Zeigler’s Adult Zebrafish Complete Diet (Aquatic Habitats, Apopka, FL, USA)	(Wang et al. (33)
OBS (flame retardant)	CAS No 70,929-87-7 (97% purity), Shanghai Futian Chemical Technology Co., Ltd. (Shanghai, China)	The exposure solutions (150 embryos/100 ml until 7 dpf; larvae after 7 days in 1 L exposure solution, after 14 days in 2.5 L exposure solutions), were half renewed every day during the first 7 days exposure, and renewed every other day after first 7 days until next 14 days (total exposure duration 21 days)	Guide for the care and use of laboratory animals of China	Fed with egg yolk suspension from 5–14 dpf and Artemia nauplii twice daily after 14 dpf.	(Zhao et al. (38)
^4^ Pb (metal)	Lead acetate trihydrate (PbAC, 3H_2_O; 99% purity) Sinopharm Chemical Company Ltd., Shanghai, China	Exposure solution was renewed daily containing 0.001% DMSO [the duration of exposure was 3 months]		Adults are fed twice daily with flake food and newly hatched *Artemia nauplii*. [stocking density 10 males and 10 females in 10 L media]	(Chen et al. (27)
TBT ^2^ (biocides)	Sigma-Aldrich (St. Louis, MO, USA)	Half of the solution was changed every day [exposure duration was 90 days]	Wayne State University	Adult fish fed with freshly hatched brine shrimp (*Artemia salina*)	(Li and Li (32)
TCEP (Flame retardants)	CAS 115-96-8 (<97% purity), Sigma-Aldrich Chemical Co. (St. Louis, USA)	40 ml of exposure solutions refreshed daily (duration of exposure 120 hpf)	College of Animal Science, Fujian Agricultural and Forestry University (GB/T 35892-2018)	Adult fish fed 3 times/day with live brine shrimp (*Artemis nauplii*) and commercial fish food	(Hu et al. (30)
TDCPP^3^ (Flame retardants)	CAS#13674-87-8) Tokyo Chemical Industry Co. (Tokyo, Japan).	Media replaced daily, consisting of 0.001% DMSO [duration of exposure 3 months]	National Institute for Food and Drug Control of China.	10 adult fish (single sex)/tank	(Wang et al. (31)
THM (Biocides)	95% pure; Beijing Huarong Biological Hormone Plant	Acetone was used as solvent (0.01%)	Chinese Academy of Agricultural Sciences		(Yang et al. (37)
n-TiO_2_^1^ (metal)	Hangzhou Wan Jing New Material Company, China (purity 99.9%); CAS 13463-67-7). Average diameter 5 nm.	Exposure solution renewed daily with 0.005% DMSO and treatment continued for 4 months.	National Institute for Food and Drug Control of China.	Adult fish fed twice daily with pellet food (Zeigler Bros Inc., Gardners, PA, USA) [stocking density 12 males and 20 females in 20 L exposure medium]	(Guo et al. (28)

^1^Bisphenol A was dissolved in 0.005% DMSO and TiO_2_ added either alone or in combinations.

^2^Cd and TBT were added either alone or in combinations.

^3^Feeding conditions were not mentioned.

^4^Pb and BDE-209 were exposed either alone or in combinations.

**Table 3:** Toxicological effects EDCs on zebrafish during embryo-larval development and in adult stages.

Name of the chemicals and concentrations	Developmental stage	Duration of exposure	Evaluation stages	Morphological endpoints	References
ATZ (0.3, 3, and 30 μg/L)	Embryos	1–72 hpf	9, 12, and 14 months	1. No significant effect on the body weight at 14 months of age 2. Global methylation status (5 mC percentage) did not significantly change during 12 months of age	(Horzmann et al. (69)
BPA (1, 10, 100, 1,000, and 10, 000 nM) [0.223–2282.9 μg/L]	Embryos	0–5 dpf	24, 48, 72, 96, and 120 hpf	1. Induced significant concentration-dependent skeletal and total abnormalities. 2. Other abnormalities, like uninflated swim bladder, cardiac edema, yolk sac edema did not show any significant difference.	(Wu et al. (36)
BPA (1, 10, 100, 1,000, and 10, 000 nM)	larvae	4–5 dpf	5 dpf	1. A trend of increasing skeletal abnormalities, uninflated swim bladder, and total abnormalities were observed, (not significantly different from controls)	(Wu et al. (36)
BPS (1, 10, and 100 μg/L)	Embryos	2 hpf–120 dpf	F1 eggs, derived from F0 parents.	1. The head-trunk angles (HTA) were significantly reduced and the length of otic vesicles significantly increased in embryos (30 hpf) 2. Hatching delayed in embryos	(Wei et al. (35)
BPS (1, 10, and 100 μg/L)	Embryos	2 hpf–120 dpf	F1 larvae (96 hpf), derived from F0 parents.	1. Inflation of the swim bladder significantly decreased. 2. The number (concentration-dependent) and area (in all concentrations) of lateral stripe melanocytes were significantly reduced.	(Wei et al. (35)
E1 (0.01, 0.1, 1, and 10 nm) [.027–2.7μg/L)	Embryos	0–5 dpf	24, 48, 72, 96, and 120 hpf	1. Significant skeletal abnormalities 2. Uninflated swim bladder 3. Total abnormalities not significantly different	(Wu et al. (36)
E1 (0.01, 0.1, 1, and 10 nm)	Larvae (4 dpf)	94 hpf–120 hpf	120 hpf	1. Significant skeletal abnormalities 2. Uninflated swim bladder 3. Total abnormalities are not significantly different from controls	(Wu et al. (36)
mBDE-47 (1, 10, 50, and 100 nM)	Embryos	4–96 hpf	22, 24, 30–34 and 36 hpf	1. Significantly delayed the reduction in coiling frequency in a concentration and time-dependent manner.	(Wang et al. (33)
mBDE-47 (1, 10, 50, and 100 nM) + tshβ mRNA (3 nl of 265 ng/μl)	Embryos	4–26 hpf	26 hpf	1. thrβ mRNA partially rescued the embryos from the toxic effects (disrupting coiling frequency) of mBDE-47	(Wang et al. (33)
OBS	Embryos (F0)	2 hpf–21 dph	Eggs (F1) , derived from F0 parents	1. The rates of hatching, did not show any significant difference	(Zhao et al. (38)
OBS	Embryos (F0)	2hpf–21 dph	F1 Larvae (7 dpf), derived from F0 parents	2. The malformation rates, survival, and the growth (lengths and weight) of the hatched larvae (7 dpf) did not show any significant difference	(Zhao et al. (38)
TCEP	Embryos	120 hpf	48, 72, 96, and 120 hpf	1. Significant development-specific concentration-dependent reduction in heartbeats was observed in embryos (200μg/L; 48 hpf). 2. A concentration-dependent inhibition (not significant) in hatching rates of the embryos (20 and 200 μg/L) was observed only during 72 hpf.	(Hu et al. (30)
TCEP	Embryos	120 hpf	120 hpf	1. The length of the larvae reduced in a concentration-dependent manner (20–200 μg/L) in 72 and 120 hpf of development. 2. Did not affect the malformation rates of the larvae (yolk sac edema, tail deformation, bent spine)	(Hu et al. (30)
THM	Embryos	4–6 days	4–6 days	1. Hatching delayed 2. Induced pericardial edema (72 hpf)	(Yang et al. (37)
THM	Embryos	4–6 days	Larvae (96 hpf)	1. Larval length significantly decreased. 3. Induced spinal curvature	(Yang et al. (37)
BDE-209 (1, 10, and 100 μg/L)	Adults	4 months	F0 (parents)	4. Growth (length and weight) of male fish did not significantly alter. 5. In females, significant nonmonotonic increase in weight was observed (1 μg/L BDE-209). 6. Significant nonmonotonic sex-specific alteration in the condition factor of the male fish (enhanced in 1 μg/L and reduced in 100 μg/L) 7. The HSI and BSI in male and HSI of female fish remained unaltered. 8. BSI in females, showed a concentration-dependent increase. 9. Fecundity (eggs/female/day) remained unaltered	(Chen et al. (27)
BDE-209 (1, 10, and 100 μg/L) + Pb (10 μg/L)	Adults (F0)	4 months	F0 (parents)	1. Although the length remained unaltered, the weight of the male fish significantly reduced by BDE-209 (1 μg/L) and enhanced in 100 μg/L in presence of Pb (10 μg/L). 2. BDE-209 (1 μg/L) significantly reduced the condition factor of male fish in presence of Pb (10 μg/L) 3. In females, BDE-209 (10 μg/L) in presence of Pb (10 μg/L) enhanced the weight of the fish. The length and the condition factor remained unaltered. 4. The HSI and BSI of male and female fish remained unaltered in coexposure (BDE-209+Pb). However, significant reduction in brain weight (BSI) was observed in females exposed to BDE-209 (100 μg/L) with Pb. 5. The GSI of the male fish significantly decreased by BDE-209 (10 and 100 μg/L) in presence of Pb in a concentration-dependent manner. 6. In contrast to males, significant increase in GSI was observed in females exposed only to BDE-209 (10 μg/L) in presence of Pb. 10. Significant concentration-dependent reduction in fecundity [BDE-209 (100 μg/L) + Pb (10 μg/L)]	(Chen et al. (27)
BDE-209 (1, 10, and 100 μg/L)	Adults (F0)	4 months	Eggs (F1) and larvae (F1) [derived from F0 parents]	1. Size of the laid eggs remained unaltered. 2. Significant concentration-dependent accumulation of PBDE (total) was found in F1 eggs. 11. No effect on hatching, and growth (length and weight) of the larvae	(Chen et al. (27)
BDE-209 (1, 10, and 100 μg/L) + Pb (10 μg/L)	Adults (F0)	4 months	Eggs (F1) and larvae (F1) [derived from F0 parents]	1. Compared to BDE-209 alone, Pb in the media further increased the concentration of PBDE in the eggs 2. Size of the laid eggs increased only by BDE-209 (1 μg/L) in presence of Pb 3. Hatching was significantly delayed. 12. The growth (length and weight) of the larvae did not show significant alteration	(Chen et al. (27)
BPA (2 and 20 μg/L)	Adults (F0)	3 months	F0 adult males and females	1. Concentration-dependent increase in the BPA content in the body burden of both males and females	(Guo et al. (28)
BPA (2 and 20 μg/L)	Adults (F0)	4 months	F1 eggs, derived from F0 parents	1. Concentration-dependent increase in the BPA content in the F1 eggs.	(Guo et al. (28)
BPA (2 and 20 μg/L)	Adults (F0)	4 months	F1 larvae (5–10 dpf), derived from F0 parents	1. Hatching rates are significantly delayed in a concentration-dependent manner. 2. Concentration-dependent increase in malformations (pericardial edema and axial spinal curvature) 3. Concentration-dependent decrease in survival rates of the larvae compared with controls. 2. Concentration-dependent decrease in the growth of the larvae observed on 10 dpf larvae	(Guo et al. (28)
TiO2 (100 μg/L)	Adults (F0)	4 months	F0 males and females	1. Accumulation of Ti was observed in the body burdens of both males and females	(Guo et al. (28)
TiO2 (100 μg/L)	Adults (F0)	4 months	F1 (eggs), derived from F0 parents	1. Significant amount of Ti was accumulated in the F1 eggs	(Guo et al. (28)
TiO2 (100 μg/L)	Adults (F0)	4 months	F1 larvae (5–10 dpf), derived from F0 parents	1. No effect was observed in hatching, malformation, and survival of the larvae	(Guo et al. (28)
BPA (2 and 20 μg/L) + TiO2 (100 μg/L)	Adults (F0)	4 months	F0 males and females	1. Both BPA and Ti were accumulated in the body burdens of both male and female fish which was significantly higher than the fish exposed either to Ti or BPA alone.	(Guo et al. (28)
BPA (2 and 20 μg/L) + TiO2 (100 μg/L)	Adults (F0)	4 months	F1 eggs, derived from F0 parents	1. Both BPA and Ti were accumulated in F1 eggs which was significantly higher than the fish exposed either to Ti or BPA alone.	(Guo et al. (28)
BPA (2 and 20 μg/L) + TiO2 (100 μg/L)	Adults (F0)	4 months	F1 larvae (5–10 dpf), derived from F0 parents	Concentration-dependent decrease in hatching, malformation, survival, and weight of the larvae (F1) when compared with controls	(Guo et al. (28)
Pb (10 μg/L)	Adults	4 months	F0 adults	1. No significant effect was observed in length and weight of the F0 parents (male and females), however, the condition factor in F0 males decreased significantly. 2. The HSI (liver), GSI (gonad), and BSI (brain) remained unaltered. 3. Fecundity remained unaltered after Pb exposure.	(Chen et al. (27)
Pb (10 μg/L)	Adults	4 months	F1 eggs, derived from F0 parents	1. A significant amount of Pb was accumulated in the eggs. 2. No significant effect was observed in the size and hatching rates of eggs (F1)	(Chen et al. (27)
Pb (10 μg/L)	Adults	4 months	F1 larvae (5 dpf), derived from F0 parents	1. No significant effect on development was observed 2. No significant effect was observed on the length and weight of the larvae (F1), however the condition factor significantly reduced when compared with controls (no Pb). 3. A significant amount of Pb was accumulated in the larvae.	(Chen et al. (27)
TDCPP (4, 20, and 100 μg/L)	Adults (F0)	3 months	adults	1. A concentration-dependent sex-specific accumulation (females appears to accumulate more than males) of TDCPP was observed (body burden) 2. Significant amount of BDCPP (metabolite of TDCPP) also detected in the body burden of both male and female fish.	(Wang et al. (31)
TDCPP (4, 20, and 100 μg/L)	Adults (F0)	3 months	Eggs (F1), derived from F0 parents	1. Significant amount of TDCPP was detected in the eggs of the fish (concentration-dependent). However, the metabolite BDCPP was found to be very minimum (undetectable/minimum) 2. The hatching of the embryos (3 dpf) was significantly reduced (concentration-dependent)	(Wang et al. (31)
TDCPP (4, 20, and 100 μg/L)	Adults (F0)	3 months	larvae (F1) (5 and 10 dpf), derived from F0 parents	1. The growth of the larvae was inhibited in a concentration-dependent manner. 2. The malformation (spinal curvature) increased, and survivability of the larvae (5 and 10 dpf) was significantly decreased in a concentration dependent. 3. The ROS content in the larvae (10 dpf) was found to be significantly increased (concentration-dependent)	(Wang et al. (31)
TBT (100 ng/L)	F1	7 days	Larvae, derived from F0 parents	1. No significant alterations in survival rates and hatching of the embryos. 2. TBT exposure alone did not induce any significant change in heart rates. 3. Significant decrease in the larval length (7 dpf) was observed in fish exposed to TBT	(Li and Li (32)
TBT (100 ng/L)	F2	7 days	F2 larvae, derived for F1 parents	1. Survivability during embryo-larval development was significantly decreased in fish exposed to TBT. 2. Hatching was not significantly different from controls. 3. Significant decreases in heart rates 4. Significant induction in the malformation rates and reduction in the length of the larvae	(Li and Li (32)
TBT (100 ng/L) + Cd (100 ng/L)	F1 larvae [derived from the parents exposed to TBT (100 ng/L) and Cd (100 ng/L) for 3 months]	7 days	Larvae, derived from F0 parents	1. No significant alterations in survival rates and hatching of the embryos. 2. A significant decrease in heart rates was observed. 3. Significant decrease in the larval length (7 dpf)	(Li and Li (32)
TBE (100 ng/L) +Cd (100 ng/L)	F2 larvae	7 days	Larvae, derived from F1 parents	1. Survivability during embryo-larval development was significantly decreased. 2. Hatching was significantly delayed. 3. Significant decreases in heart rates 4. Significant induction in the malformation rates and reduction in the length of the larvae (7 dpf) was observed	(Li and Li (32)
Cd (100 ng/L)	F1	7 days	Larvae, derived from F0 parents	1. No significant alterations in survival rates and hatching of the embryos. 2. No significant change in heart rates. 3. No significant change in the length of the larvae (7 dpf)	(Li and Li (32)
Cd (100 ng/L)	F2	7 days	Larvae, derived from the F1 parents	1. Survivability during embryo-larval development was significantly decreased. 2. Hatching was not significantly differed. 3. No change in heart rates 4. Significant reduction in the length of the larvae (7 dpf)	(Li and Li (32)

**Table 4:** Effects of EDCs on the endocrine system of zebrafish during embryo-larval development and in adult stages.

Name of the chemicals and concentrations	Developmental stage	Duration of exposure	Evaluation stages	Endocrinological endpoints	References
ATZ (0.3, 3, and 30 μg/L)	Embryos	1–72 hpf	9, 12, and 14 months	1. Transcriptomic analysis identified altered gene expression during reproductive system development	(Horzmann et al. (69)
BPS (1, 10, and 100 μg/L)	Embryos (2 hpf)	4 months	F0 adults	1. In females, plasma T4 levels significantly decreased, and plasma T3 levels significantly increased. *2.* In males, T4 levels in plasma remained unaltered; however, T3 levels significantly increased in 1 and 10 μg/L unaltered in fish exposed to 100 μg/L.	(Wei et al. (35)
BPS (1, 10, and 100 μg/L)	Embryos (2 hpf)	4 months	F1 eggs (derived from F0 parents)	1. T4 concentrations significantly decreased and T3 concentrations significantly increased in the F1 eggs	(Wei et al. (35)
mBDE-47 (1,10, 50, and 100 nm) + *thrβ* mRNA (3nL of 265 μg/mL)	Embryos	26 h	26 hpf	1*. thrβ* mRNA rescued the embryos from the toxic effects of mBDE-47 (delayed coiling) 2. *thrβ* mRNA protects the neurons from apoptosis induced by mBDE-47 in embryos (26 hpf)	(Wang et al. (33)
OBS (3, 30, and 300µg/L)	Embryos (F0)	2 hpf–21 dph	F0 parents (both male and females (180 dpf)	1. The average colloid area in the thyroid of male and female fish did not show any sex-specific difference. 2. The height of the epithelial cells in the thyroid significantly increased in male and female fish. 3. The thickening of the follicular area and depletion of the colloid were also observed in both sexes. 4. T3 levels in plasma of both male and female fish showed non-monotonic enhancements when exposed to lower concentrations of OBS (3 μg/L in females and 3 and 30 μg/L in males). 5. T4 levels in females showed a significant concentration-dependent decrease, and in males significant concentration-dependent increase was observed.	(Zhao et al. (38)
OBS (3, 30, and 300µg/L)	Embryos (F0)	2 hpf–21 dph	Eggs (F1), derived from F0 parents	1. The T3 contents showed significant increase while T4 levels remained unaltered.	(Zhao et al. (38)
OBS (3, 30, and 300 µg/L)	F0 Embryos (2 hpf)	2 hpf–21 dpf	F1 larvae (7 dpf), derived from F0 parents	1. The T3 content in the whole larvae (7 dpf) was significantly decreased in lower concentration (3 μg/L) and increased in higher concentrations of OBS groups (30 and 300 μg/L) 2. T4 content decreased significantly	(Zhao et al. (38)
TCEP (0.2, 2, 20, and 200 µg/L)	Embryos	120 hpf	Larvae (120 hpf)	1. T3 levels of larvae (120 hpf) did not show any significant difference, 2. T4 levels were significantly reduced in a concentration-dependent manner	(Hu et al. (30)
THM (0.19, 1.90, and 2.85 mg/L)	Embryos	4–6 days	Larvae (96 hf)	1. The thyroid releasing hormone (TRH) and thyroid-stimulating hormones (TSH) did not show significant changes 2. The T3 and T4 contents in the larval body (96 hpf) were significantly reduced in a concentration-dependent manner	(Yang et al. (37)
BDE-209 (1, 10, and 100 μg/L)	Adults	4 months	F0 (male and female parents )	1. A significant concentration-dependent increase in serum T3 level was observed in male fish 2. No significant alteration in the serum T4 levels of male fish 3. In females, compared with controls, serum T3 level significantly decreased 4. Serum T4 contents in females decreased (concentration-dependent) 5. Serum testosterone (T) levels in males remained unaltered 6. Serum T levels in females significantly decreased. 7. The serum E2 levels in male fish enhanced in a concentration-dependent manner. 8. In females, serum E2 levels significantly decreased (concentration-dependent)	(Chen et al. (27)
BDE-209 (1, 10, and 100 μg/L)	Adults	4 months	F1 eggs (derived from parental exposure)	1. A significant nonmonotonic decrease in T3 content in eggs (F1) was observed [decreasing only in eggs derived from parents exposed to 1μg/L, higher exposed concentrations are ineffective] 2. T4 contents in eggs (F1) remained unaltered. 3. Concentration of T significantly reduced in eggs (F1) 4. E2 content in eggs was increased in a concentration-dependent manner	(Chen et al. (27)
BDE-209 (1, 10, and 100 μg/L)	Adults	4 months	F1 larvae (5dpf) [derived from parents exposed BDE-209]	1. T3 contents of the larvae were increased in a concentration-dependent manner 2. T4 contents in larvae remained unaltered. 3. The T contents in larvae showed a significant reduction 4. The E2 content in larvae showed a significant concentration-dependent reduction	(Chen et al. (27)
BDE-209 (1, 10, and 100 μg/L) + Pb (10 μg/L)	Adults (F0)	4 months	F0 Adults (male and female parents)	1. A significant concentration-dependent increase in serum T3 level was observed in male fish by BDE-209 (100 μg/L) when coexposed with Pb (10 μg/L). 2. Presence of Pb (10 μg/L) was unable to induce significant alteration in the serum T4 levels of male fish exposed to BDE-209 (1 and 100 μg/L); however, BDE-209 at 10 μg/L significantly increased serum T4 level when coexposed with Pb (10 μg/L) 3. In females, coexposure with lead 10 μg/L), significantly decreased serum T3 levels when BDE-209 concentrations are 1 and 10 μg/L. 4. Significant concentration-dependent decrease in serum T4 levels in females by BDE-209 (100 μg/L), when coexposed with lead (10 μg/L) 5. Coexposure of BDE-209 with lead (10 μg/L) significantly reduced serum T levels in male fish. 6. In females, BDE-209 + Pb was able to significantly reduce serum T levels 7. The serum E2 levels in male fish were reduced significantly by BDE-209 and Pb coexposure 8. In females, a significant reduction in serum E2 levels was observed in combined exposure conditions (BDE-209+Pb)	(Chen et al. (27)
BDE-209 (1, 10, and 100 μg/L) + Pb (10 μg/L)	Adults (F0)	4 months	F1 eggs derived from F0 parents	1. T3 content in eggs derived from parents exposed to BDE-209 (10 and 100 μg/L) and Pb (10 μg/L) significantly reduced. 2. T4 contents significantly reduced in eggs derived from the fish coexposed with Pb (10 μg/L) and BDE-209 (10 and 100 μg/L) 3. Concentration of T significantly reduced in eggs derived from parents exposed to BDE-209 (1, 10 μg/L) and Pb (10 μg/L) in combinations. While enhanced if the concentration of BDE-209 increased to 100 μg/L) 4. E2 content in eggs derived from parents exposed to Pb (10 μg/L) in combinations with BDE-209 (1, and 10 μg/L+ Pb) did not show any significant effect. However, significant (E2) enhancement was observed in eggs derived from the fish exposed to BDE 209 (100 μg/L) and Pb (10 μg/L) in combinations	(Chen et al. (27)
BDE-209 (1, 10, and 100 μg/L) + Pb (10 μg/L)	Adults (F0)	4 months	F1 larvae (5 dpf) derived from F0 parents	1. T3 content significantly increased in larvae derived from parents coexposed with lead (10 μg/L) and BDE-209 (10 and 100 μg/L). 2. A significant increase in T4 contents was observed in larvae derived from parents coexposed with BDE-209 (1,10, 100 μg/L) and Pb (10μg/L). 3. The T contents in larvae derived from the parents exposed to BDE-209 (1, 10, and 100 μg/L) and Pb (10 μg) in combination showed a significant reduction 4. E2 content in larvae derived from the parents exposed to BDE-209 (1, 10, and 100 μg/L) in combination with Pb (10 μg/L) significantly reduced	(Chen et al. (27)
BPA (2 and 20 μg/L)	Adults (F0)	4 months	F0 Adults (male and female)	1. T4 level in females, not in males, was significantly decreased in a concentration-dependent manner 2. T3 remained unaltered in both male and females	(Guo et al. (28)
BPA (2 and 20 μg/L)	Adults (F0)	4 months	F1 eggs (derived from F0 parents)	1. T4 contents significantly reduced in a concentration-dependent manner 2. T3 level remained unaltered	(Guo et al. (28)
BPA (2 and 20 μg/L)	Adults (F0)	4 months	F1 larvae (10 dpf), derived from F0 parents	1. T4 content in the F1 larvae (10 dpf) decreased significantly in a concentration-dependent manner 2. T3 level remained unaltered.	(Guo et al. (28)
Pb (10 μg/L)	Adults (F0)	3 months	F0 Adults (male and female)	1. No effect in the serum T3 content of male and female fish (F0). 2. Serum T4 content in male fish remained unaltered, while in females, significant decrease the T4 levels occurred. 3. Serum T and E2 level in male fish remained unaltered, while in females, both T and E2 levels in serum significantly reduced.	(Chen et al. (27)
Pb (10 μg/L)	Adults (F0)	3 months	F1 eggs derived from F0 parents	1. Both T3 and T4 contents of F1 eggs remained unaltered. 2. The T content of F1 eggs significantly decreased, while E2 content remained unaltered.	(Chen et al. (27)
Pb (10 μg/L)	Adults (F0)	4 months	F1 larvae (5 dpf) derived from F0 parents	1. Both T3 and T4 contents of F1 larvae (5 dpf) remained unaltered 2. The T content of F1 larvae significantly decreased while E2 content remained unaltered.	(Chen et al. (27)
TiO2 (100 μg/L)	Adults (F0)	4 months	F0 Adults (male and female)	1. Both T3 and T4 levels remained unaltered	(Guo et al. (28)
TiO2 (100 μg/L)	Adults (F0)	4 months	F1 eggs (derived from F0 parents)	1. Both T3 and T4 levels remained unaltered	(Guo et al. (28)
TiO2 (100 μg/L)	Adults (F0)	4 months	F1 larvae (5–10 dpf) [derived from F0 parents]	1. Both T3 and T4 levels remained unaltered	(Guo et al. (28)
BPA (0.2 and 20 μg/L) + TiO2 (100 μg/L)	Adults (F0)	4 months	F0 Adults (male and female)	1. T4 level was significantly reduced both in males and females in a concentration-dependent manner 2. T3 levels were significantly reduced only in females, not in males, in a concentration-dependent manner	(Guo et al. (28)
BPA (2 and 20 μg/L) + TiO2 (100 μg/L)	Adults (F0)	4 months	F1 eggs (derived from F0 parents)	1. T4 level significantly reduced in a concentration-dependent manner 2. T3 level remained unaltered.	(Guo et al. (28)
BPA (2 and 20 μg/L) + TiO2 (100 μg/L)	Adults (F0)	4 months	F1 larvae (5–10 dpf)	1. Both T3 and T4 levels significantly reduced in a concentration-dependent manner.	(Guo et al. (28)
TDCPP (4, 20, and 100 μg/L)	Adults	3 months	F0 adults; males and females	1. Plasma T4 level in females was significantly reduced in a concentration-dependent manner (20-100 μg/L) 2. Plasma T3 levels in females significantly reduced in a concentration-dependent manner (100 μg/L). 3. In males no significant change in the thyroid hormone levels (T3 and T4) in the plasma of the fish.	(Wang et al. (31)
TDCPP (4, 20, and 100 μg/L)	Adults	3 months	F1 eggs, derived from F0 parents	1. T4 levels were significantly reduced in a concentration-dependent manner (only parents exposed to 100 μg/L TDCPP). 2. T3 content remained unaltered.	(Wang et al. (31)
TDCPP (4, 20, and 100 μg/L)	Adults	3 months	F1 larvae (5 and 10 dpf), derived from F0 parents	1. T4 level significantly reduced in both 5 dpf and 10 dpf larvae in a concentration-dependent manner 2. T3 content remained unaltered (both 5 and 10 dpf)	(Wang et al. (31)
TBT (100 ng/L)	F0	3 months	adults	1. In both male and female fish, plasma T3 and T4 levels remained unaltered	(Li and Li (32)
TBT (100 ng/L)	F1	7 days	Larvae, derived from F0 parents	1. The T3 and T4 contents of the whole body of the larvae (7 dpf) remained unaltered	(Li and Li (32)
TBT (100 ng/L)	F1	5 months	Adults, derived from F0 parents.	1. In F1 fish (both male and females), plasma T3 content remained unaltered. 2. Plasma T4 level in male fish remained unaltered 3. Plasma T4 level in female fish significantly reduced	(Li and Li (32)
TBT (100 ng/L)	F2	7 days	Larvae (derived from F1 parents)	1. Compared with controls, both T3 and T4 contents in the whole body of the larvae significantly reduced	(Li and Li (32)
TBT (100 ng/L) + Cd (100 ng/L)	F0	3 months	Adults	1. Thyroid hormones (T3 and T4) levels in males remained unaltered, but in females TH levels (both T3 and T4) significantly reduced	(Li and Li (32)
TBT (100 ng/L) + Cd (100 ng/L)	F1	Larvae, derived from F0 parents	7 days	1. The T3 and T4 contents of the whole body of the larvae significantly reduced.	(Li and Li (32)
TBT (100 ng/L) + Cd (100 ng/L)	F1	5 months	Adults, derived from F0 parents	1. Coexposure (Cd+ TBT) significantly reduced plasma T3 content in male and female fish. 2. Coexposure (Cd+ TBT) significantly reduced T4 levels in plasma of male fish 3. Plasma T4 level in female fish significantly reduced in fish exposed in combinations (TBT+ Cd)	(Li and Li (32)
TBT (100 ng/L) + Cd (100 ng/L)	F2	7 days	Larvae, derived from F1 parents	1. Both T3 and T4 contents in the whole body of the larvae significantly reduced in fish exposed to combinations of TBT and Cd (TBT+ Cd).	(Li and Li (32)
Cd (50 ng/L)	F0	3 months	Adults	1. In both male and female fish, plasma T3 and T4 remained unaltered	(Li and Li (32)
Cd (50 mg/L)	F1	7 days	Larvae, derived from F0 parents	1. The T3 and T4 contents of the whole body of the larvae (7 dpf) remained unaltered	(Li and Li (32)
Cd (50 ng/L)	F1	5 months	Adults, derived from F0 parents	1. In F1 fish (both male and female), plasma T3 content remained unaltered 2. Plasma T4 level in male fish remained unaltered. 3. Plasma T4 level in female fish significantly reduced	(Li and Li (32)
Cd (50 ng/L)	F2	7 days	Larvae, derived from F1 parents	1. Both T3 and T4 contents in the whole body of the larvae significantly reduced in fish	(Li and Li (32)

**Table 5:** Effects of EDCs on the Neurobehavioral disorders during embryo larval development and in adult zebrafish.

Name of the chemicals and concentrations	Developmental stage	Duration of exposure	Evaluation stages	Neurological and behavioral endpoints	References
ATZ (0.3, 3, and 30 μg/L)	Embryos	1–72 hpf	9, 12, and 14 months	1. At 9 months post-fertilization males showed decreased locomotor parameters. 2. No significant difference was observed in brain size, brain weight, and cranio-somatic index (brain weight X100)/body weight) at 14 months of age. 3. Histopathological analysis of the brain identified morphometric differences and reduced number of cells in the raphe populations.	(Horzmann et al. (69); Wang et al. (34)
BPA (1, 10, 100, 1,000, and 10,000 nM)	Embryos	0–5 dpf	24, 48, 72, 96, and 120 hpf	1. Distance travelled in the dark were significantly higher (significant only 100 and 10,000 nM concentrations)	(Wu et al. (36)
BPA (1, 10, 100, 1,000, and 10,000 nM)	larvae	4–5 dpf	120 hpf	1. Distance traveled in dark is significantly less (except 1,000 nM) than the control groups	(Wu et al. (36)
BPS (1, 10, and 100 μg/L)	Embryos (2 hpf)	4 months	F1 embryos (derived from F0 parents)	1. Spontaneous movement (touch-evoked escape response) was significantly reduced in F1 embryos (30 hpf) 2. The average swimming speed and the swirl-escape rate was also significantly reduced in F1 larvae (96 hpf)	(Wei et al. (35)
E1 (0.01, 0.1, 1, and 10 nm)	Embryos	0–5 dpf	24, 48, 72, 96, and 120 hpf	1. The distance travelled in the dark is significantly decreased in a non-monotonic manner	(Wu et al. (36)
E1 (0.01, 0.1, 1, and 10 nm)	larvae	4–5 days	120 hpf	1. The distance travelled in dark inconsistently decreased only in two concentrations (0.01 and 100 nm)	(Wu et al. (36)
mBDE-47	Embryos	4–96 hpf	22, 26, 30, 34, and 96 hpf	2. Reduction of coiling behavior frequency was significantly delayed in a time and concentration-dependent manner. 3. Enhanced neuronal apoptosis in the brain of the embryos (26 hpf) 4. Reduced the number of 5-HT immunoreactive neurons in the hypothalamus of the larvae (96 hpf)	(Wang et al. (33)
OBS	Embryos (F0)	2 hpf–21 dph	F1 Larvae (7 dpf), derived from F0 parents	1. The swirl-escape rate in the F1 larvae (7 dpf) significantly decreased in a concentration-dependent manner	(Zhao et al. (38)
THM	Embryos	4–6 days	Larvae (96 hpf)	1. The distance moved, the average speed, and the larval activity was significantly inhibited. 2. The neurotransmitters, serotonin (5-HT) and norepinephrine significantly increased, and the AChE activity significantly decreased in larvae (concentration-dependent)	(Yang et al. (37)
BDE-209 (1, 10, and 100 μg/L)	Adults (F0 parents)	4 months	F1 larvae (5 dpf), derived from F0 parents	1. The average swimming speed in both light and dark phages decreased significantly. 2. Among the 12 neurotransmitters studied, only ACh was found to upregulate in larvae derived from the parents exposed to BDE-209 (1 μg/L) 3. GABA, tryptophan, and choline were upregulated, and glutamate was downregulated in larvae derived from parents exposed to 10μg/L BDE-209. 4. Glutamate, GABA, and tryptophan were upregulated while 5-HIAA was downregulated in larvae derived from the parents exposed to BDE-209 (100 μg/L).	(Chen et al. (27)
BDE-209 (1, 10, and 100 μg/L) + Pb (10 μg/L)	Adults (F0 parents)	4 months	F1 larvae (5 dpf), derived from F0 parents	1. The average swimming speed in both light and dark phages decreased significantly in larvae. 2. Among the 12 neurotransmitters studied, GABA, and ACh was upregulated in larvae derived from the parents coexposed to BDE-209 (1μg/L) and Pb (10 μg/L); while GABA was upregulated and glutamate, ACh, and norepinephrine were downregulated in larvae derived from parents exposed to 10 μg/L BDE-209+Pb (10 μg/L) and glutamate, 3. GABA, ACh and norepinephrine were tended to downregulate in larvae derived from parents co-exposed with BDE-209 (100 μg/L) + Pb (10 μg/L)	(Chen et al. (27)
BPA (2 and 20 μg/L)	Adults (F0)	4 months	Adults’ male and female (F0 parents)	1. In the brain of both male and female fish, the serotonin, dopamine, and acetylcholine contents remained unaltered.	(Guo et al. (28)
BPA (2 and 20 μg/L)	Adults (F0)	4 months	F1 larvae (5–10 dpf)	1. Significant concentration-dependent reduction in the swimming speed of the larvae in light phase 2. During the light-dark transition, the average swimming speed was significantly lower than the control larvae. 3. The neurotransmitters, serotonin and dopamine significantly reduced, while no effect was observed in the ACh contents. 4. AChE activity remained unaltered	(Guo et al. (28)
TiO2 (100 μg/L)	Adults (F0)	4 months	Adults (F0 parents)	1. In females, the serotonin and dopamine contents in the brain significantly reduced while the ACh contains remained unaltered. 2. In males, TiO2 did not induce any significant change in the serotonin, dopamine, or ACh contents in the brain	(Guo et al. (28)
TiO2 (100 μg/L)	Adults (F0)	4 months	F1 (5–10 dpf), derived from F0 parents	1. Significant decrease in the swimming speed of the larvae during light phase. 2. During the light- dark transition, the average swimming speed was significantly lower. 3. Significant decrease in the serotonin and dopamine contents, while ACh contents and AChE activity remained unaltered	(Guo et al. (28)
BPA (2 and 20 μg/L) + TiO2 (100 μg/L)	Adults (F0)	4 months	F0 adults (male and female)	1. In females, the serotonin and dopamine contents significantly reduced in a concentration-dependent manner, while ACh contents remained unaltered. 2. In males, no significant effect was observed in the serotonin, dopamine, and ACh contents of the brain.	(Guo et al. (28)
BPA (2 and 20 μg/L) + TiO2 (100 μg/L)	Adults (F0)	4 months	F1 larvae (5–10 dpf), derived from F0 parents	1. Significant concentration-dependent reduction in the swimming speed of the larvae in light phase 2. During the light- dark transition, the average swimming speed was significantly lower than the control larvae. 3. Concentration-dependent decrease in the serotonin, and dopamine contents of the larvae 4. ACh content. remained unaltered, while AChE activity significantly reduced in a concentration-dependent manner.	(Guo et al. (28)
TDCPP (4, 20, and 100 μg/L)	Adults	3 months	F1 larvae (5 and 10 dpf), derived from F0 parents	1. The swimming speed significantly reduced in a concentration-dependent manner in the larvae (5 dpf) during the light phase and light-dark transition phase, (significant only in larvae derived from parents exposed to 100 μg/L TDCPP for 3 months). 2. The average swimming speed in 10 dpf larvae derived from fish exposed to 100 μg/L TDCPP, was reduced significantly. 3. The neurotransmitter contents (serotonin, dopamine, GABA, and histamine) in 5 and 10 dpf larvae were altered by parental TDCPP exposure. 4. In 5 dpf larvae, histamine content remained unaltered, while serotonin content significantly decreased in a concentration-dependent manner (significant only in larvae when the parents were exposed to 100 μg/L) 5. The dopamine and GABA content was significantly reduced in 5 dpf larvae when the parents were exposed to 20 and 100 μg/L TDCPP. 6. In 10 dpf larvae, serotonin and GABA contents were reduced significantly when the parents were exposed to TDCPP (100 μg/L). 7. Dopamine and histamine contents were reduced significantly in larvae (10 dpf) when the parents were exposed to 20 and 100 μg/L TDCPP for 3 months. 8. AChE activity remained unaltered in both 5 and 10 dpf larvae derived from the parents exposed to TDCPP (4, 20, and 100 μg/L) for 3 months.	(Wang et al. (31)
Pb (10 μg/L)	Parents (F0)	4 months	F1 larvae (5 dpf), derived from F0 parents	1. The average swimming speed in both light and dark phages decreased significantly in larvae. 2. Among the 12 neurotransmitters studied, GABA, tryptophan and ACh were upregulated and glutamate was downregulated in larvae	(Chen et al. (27)
TBT	F0	3 months	Adults (parents)	1. The dopamine and serotonin contents and the AChE activity in the brain of adult male and female fish did not show any significant alteration	(Li and Li (32)
TBT	F1	7 days	Larvae (derived from F0 parents)	1. The dopamine and serotonin contents and the AChE activity in the F1 larvae (7 dpf) did not show any significant change	(Li and Li (32)
TBT	F1	5 months	Adults (derived from F0) parents)	1. The dopamine content in the brain of F1 adult males did not show any significant change. 2. In F1 adult females, TBT alone was able to significantly reduce the dopamine content in the brain of the fish. 3. The serotonin content in male brains did not show any significant change when compared with controls. 4. In females, the brain serotonin level, significantly decreased by TBT exposure. 5. The AChE activity remained unaltered in female brain by TBT exposure.	(Li and Li (32)
TBT	F2	7 days	Larvae (derived from F1 parents)	1. Dopamine and serotonin contents and the AChE activity in the larval body (7 dpf) significantly decreased	(Li and Li (32)
TBT+ Cd	F0	3 months	Adults (parents)	1. The dopamine content in the brain of adult male and female fish did not show any significant alteration. 2. The serotonin content and AChE activity in the brain of adult males did not show any significant changes; however, in females, serotonin levels in the brain significantly decreased, and AChE activity remained unaltered.	(Li and Li (32)
TBT+ Cd	F1	7 days	Larvae, derived from F0 parents	1. The dopamine and serotonin contents and the AChE activity in the F1 larvae (7 dpf), decreased significantly	(Li and Li (32)
TBT+ Cd	F1	5 months	F1 Adults, derived from F0 parents	1. The dopamine content in the brain of adult males (F1 adults) decreased significantly. 2. In F1 adult females, significant reduction in the dopamine content in the brain of the fish. 3. The serotonin content in male and female brains significantly reduced. 4. The AChE activity in the brain of male and female fish significantly decreased	(Li and Li (32)
TBT+ Cd	F2	7 days	Larvae	1. Dopamine and serotonin contents and the AChE activity in the larval body (7 dpf) significantly decreased	(Li and Li (32)
Cd	F0	3 months	Adults (F0 parents)	1. The dopamine and serotonin contents and the AChE activity in the brain of adult male and female fish did not show any significant alteration	(Li and Li (32)
Cd	F1	7 days	Larvae, derived from F0 parents	1. The dopamine and serotonin contents and the AChE activity in the F1 larvae (7 dpf) did not show any significant change	(Li and Li (32)
Cd	F1	5 months	F1 Adults, derived from F0 parents	1. The dopamine and serotonin contents in the brain of adult males and females did not show any significant change. 2. The AChE activity in the brain of male fish remained unaltered; however, decreased significantly in females	(Li and Li (32)
Cd	F2	7 days	F2 larvae, derived from F1 parents	1. Dopamine and serotonin contents, AChE activity in the larval body (7 dpf) significantly decreased	(Li and Li (32)

**Table 6:** Effects of the EDCs on the expression of genes in zebrafish embryos and adults.

Developmental stages during exposure	Name of the chemicals	Name of the studied genes	Genes upregulated	Genes downregulated
Embryos	ATZ	*ak7, aqp1a, caskin2, cavin4, ccdc39, cdk5, cyp26b1, dbt, dopey2, fam169a, ggct,htra2, ifgbp7, itm2cb, l2hgdh, mst1r, oaz2, osbpl3, pdia6, postn, prc1, prdx3, slc25a15, sult2b1, tnn12*	*ifgbp7, itm2cb, sult2b1*	*aqp1a, cdk5, cyp26b1*
Embryos	BPA	*abcg2a, agpat5, apoa1a, apoa2, apoa4b.1, apoc2*, *apoea, arhgef10, atf3, atp6ap2, atp8b, cat, cdk4, crybb1, cyp7a1, cyp7b1, dbi, ddc, dhcr24, dnmt1, dock3, dync1h1, eif3a, elovl12, elovl15, enox1, fabp6, fads2, fat2, fbl, gemin 5, gpx1a, gpx1b, gstp1, id1, lpin 1, ndufb6, ndufs4, ndufv2, osbp, pck1, plxna2, pparab, prdx1, prdx2, prdx3, prdx4, rack1, rho, rpl35a, rps13, rps19, sigmar1, slc7a1a, tgif1, tln2a, tnfrsf1a, ttr, tshz2, tubg1, txn, txn2, txnipa, txnipb, ubb, utrn, wwc1, xylt1*	*apoa1a^#^, apoa2*^#^, *apoa4b.1*^#^, *apoc2*^#^, *atp6ap2*^#^, *cat*^#^, *cdk4*^#^, *crybb1*^#^, *cyp7a1*^#^, *eif3a*^#^, *fabp6*^#^, *fads2*^#^, *gpx1a*^#^, *gpx1b^#^*, *gstp1*^#^, *id1*^#^, *ndufb6*^#^, *ndufs4*^#^, *ndufv2*^#^, *prdx1*^#^, *prdx2*^#^, *prdx3*^#^, *prdx4*^#^, *rack1*^#^, *rho*^#^, *rpl35a*^#^, *rps13*^#^, *rps19*^#^, *sigmar1*^#^, *slc7a1a*^#^, *tgif1^#^*, *tln2a^#^*, *ttr*^#^, *tshz2*^#^, *tubg1^#^*, *txn2*^#^, *txnipa*^#^	*abcg2a^#^, agpat5^#^, arhgef10*^#^, *atp8b1*^#^, *cyp7b1*^#^, *dbi*^#^, *dhcr24*^#^, *dnmt1^#^, dock3^#^*, *dync1h1^#^, elovl15^#^*, *fat2*^#^, *osbp*^#^, *plxna2*^#^, *pparab*^#^, *txnipb*^#^, *ubb*^#^, *utrn*^#^, *wwc1*^#^, *xylt1*^#^
Embryos	BPS	*Crestin, gap43, gfap, mbp*, sp*-a*, sp*-b*, sp*-c, syn2a, tyr, zhe1*	*gap43, gfap* (F1 larvae) ^#^, *mbp* (F1 larvae)	*crestin* (F1 larvae) ^#^, *sp-a* (F1 Larvae), *sp-b* (F1 larvae*)*, sp*-c* (F1 larvae), *syn2a*^#^ (F1 larvae), *tyr*^#^ (F1 larvae), *zhe1*(F1 larvae)
Embryos	E1	*abcg2a, agpat5, apoa1a, apoa2, apoa4b.1, apoc2, apoea, arhgef10, atf3, atp6ap2, atp8b1, cat, cdk4, crybb1, cyp7a1,cyp7b1, dbi, ddc, dhcr24, dnmt1, dock3, dync1h1, eif3a, elovl12, elovl15, enox1, fabp6, fads2, fat2, fbl, gemin 5, gpx1a, gpx1b, gstp1, id1, lpin 1, ndufb6, ndufs4, ndufv2, osbp, pck1, plxna2, pparab, prdx1, prdx2, prdx3, prdx4, rack1, rho, rpl35a, rps13, rps19, sigmar1, slc7a1a, tgif1, tln2a, tnfrsf1a, ttr, tshz2, tubg1, txn, txn2, txnipa, txnipb, ubb, utrn, wwc1, xylt1*	*apoea, atf3, elovl15, fbl, gemin 5,sigmar1, tnfrsf1a, txn*	*ddc, elovl12, enox1, fabp6, fads2, lpin, pck1, ttr, txnipa*
Embryos	mBDE-47	*tph2*		*tph2*
Embryos	TCEP	*dio1*(liver), *dio2* (liver), *elavl3, gap43, mbp, nis (slc5a5), syn2a, tg, trα, trβ, α1-tubulin, ugt1ab*	*elavl3*, *gap43, mbp, nis (slc5a5)*, *syn2a*, *trα, trβ, ugt1ab*	*tg, α1-tubulin*
Embryos	THM	*ahr2, angpt1, ascl1a, crh, cyp1a, dio1*(liver), *dio2* (liver), *eif1b, elavl3, flk1, hhex, ldha. left1, neurog 1, nks2.1, pax8, tg, trα, trβ, ttr, tshα, tshβ, tshγ, vegf1*	*crh, cyp1a, dio1, dio2, flk1, hhex, neurog 1, nks2.1, pax8, tg*^#^, *trα, trβ, ttr*^#^, *tshα, tshβ, tshγ, ugt1ab, vegf1*	*ldha*
Embryos	OBS	*crh, dio1*(liver), *dio2* (liver), *elavl3, gfap, mbp, nks2.1, syn2a, tg, tpo, trα, trβ, ttr, tshβ, ugt1ab* (liver)	*crh* ((♀ brain F0), *elavl3*^#^(F1larvae), *mbp*^#^(F1 larvae), *syn2a* (F1 larvae), *tpo* (F0♀^#^), *trα* (F1 larvae), *trβ* (F0 females^#^, F1 larvae), *ttr*(F0♀), *ugt1ab*(F0♂)	*dio1*(F0 ♂, F1 larvae), *dio2* (F0 ♂^#^, F0 ♀^#^, F1 larvae), *elavl3*^#^ (F1 larvae), *nks2.1*(F1 larvae), *tg* (F0 ♂, F1 larvae^#^), *tpo*(F0♂^#^), *trα*(F0♂), *tshβ* (F1 larvae), *ugt1ab* (F0♀, F1 larvae)
Adults	BPA	*crh, tshβ*	*crh* (F0), *tshβ* (F0 ♂ and F0♀)	
Adults	Cd	*crh, dio1*(liver), *dio2* (liver), *tg, ttr*(liver), *tshβ* (brain), *ugt1ab*		*crh* (F2 larvae), *dio2* (F2 larvae), *tg* (F1♀,F1 larvae, F2 larvae), *ttr*(F2 larvae), *tshβ* (F1 ♀, F2 larvae), *ugt1ab* (F2 larvae)
Adults	TBT	*crh, dio1*(liver), *dio2* (liver), *tg, ttr*(liver), *tshβ* (brain), *ugt1ab*		*crh* (F2 larvae), *dio1*(F1 larvae, F2 larvae), *tg* (F1♀, F1 larvae, F2 larvae), *ttr*(F2 larvae), *tshβ* (F1 ♀, F2 larvae), *ugt1ab* (F2 larvae)
Adults	TDCPP	*gap43, gfap, mbp, syn2a, α1-tubulin*	*gap43*(10 dpf larvae)	*mbp*^#^ (5 and 10 dpf larvae), *syn2a* (10 dpf larvae), *α1-tubulin* (both 5 and 10 dpf larvae)
Adults	Cd+TBT	*crh, dio1*(liver), *dio2* (liver), *tg* (liver*)*, *ttr*(liver), *tshβ*(brain), *ugt1ab*(liver)	*dio2* (F0 ♀), *tshβ* (F0♂and F0♀), *ugt1ab* (F0♂and F0♀)	*crh* (♀ brain F0, ♀ brain F1, larvae F2), *dio1*(F1 larvae, F2 larvae) *dio2* (F2 larvae), *tg* (F0 ♀; F1♀, F1 larvae, F2 larvae), *ttrr* (F2 larvae), *tshβ* (F1 ♀, F1 larvae, F2 larvae), *ugt1ab* (F1♀, F1 larvae, F2 larvae)

# symbol in superscript indicate the response is concentration dependent.

**Table 7:** Effects of EDCs on the expression of specific proteins in F1 larvae of zebrafish.

Name of the chemicals and concentrations	Developmental stage	Duration of exposure	Evaluation stages	Proteins	References
BDE-209 (1 μg/L)	Adults (F0)	3 months	F1 larvae (5 dpf), derived from F0 parents	1. Did not induce abnormal levels of vitellogenin in larvae (5 dpf) 2. Downregulation of cathepsin S was observed. 3. The neural protein syn2a decreased significantly in a concentration-dependent manner. 4. The neural protein mbp was decreased significantly in a concentration-dependent manner. 5. Proteomic analysis found increased and decreased levels of various crystalline proteins. 6. Proteomic analysis found increased and decreased levels of various muscle proteins in larvae. 7. Proteomic analysis found increased and decreased levels of various neuronal proteins such as internexin neuronal intermediate filament protein-alpha b, synaptic vesicle membrane protein VAT-1 homolog, syntaxin binding protein 1a, internexin neuronal intermediate filament protein alpha b, synaptotagmin binding, cytoplasmic RNA interacting protein, glial fibrillary acidic protein, myelin expression factor 2, synaptotagmin binding, cytoplasmic RNA-interacting protein, myelin expression factor 2, in larvae.	(Chen et al. (27)
BDE-209 (1 μg/L) + Pb (10 μg/L)	Adults (F0)	3 months	F1 larvae (5 dpf), derived from F0 parents	1. Downregulation of the thyroid hormone receptor protein 3b was observed. 2. Did not induce abnormal levels of vitellogenin. 3. Downregulation of cathepsin S was observed. 4. The neural protein syn2a decreased significantly in a concentration-dependent manner. 5. The neural protein mbp was decreased significantly in a concentration-dependent manner. 6. Proteomic analysis found increased and decreased levels of various crystalline proteins. 7. Proteomic analysis found increased and decreased levels of various muscle proteins in larvae. 8. Proteomic analysis found increased and decreased levels of various neuronal proteins such as internexin neuronal intermediate filament protein, alpha b, synaptic vesicle membrane protein VAT-1 homolog, syntaxin binding protein 1a, internexin neuronal intermediate filament protein alpha a, synaptotagmin binding, cytoplasmic RNA interacting protein, glial fibrillary acidic protein, myelin expression factor 2, Synaptotagmin binding, cytoplasmic RNA interacting protein, myelin expression factor 2, in larvae.	(Chen et al. (27)
Pb (10 μg/L)	Adults (F0)	3 months	F1 larvae (5 dpf), derived from F0 parents	1. Downregulation of the thyroid hormone receptor protein 3b 2. Downregulation of cathepsin S. 3. The neural protein syn2a and mbp decreased significantly. 4. Several of the crystalline proteins, muscle proteins, and neuronal proteins were altered. 5. Proteomic analysis found increased and decreased levels of various crystalline proteins. 6. Proteomic analysis found increased and decreased levels of various muscle proteins in larvae. 7. Proteomic analysis found increased and decreased levels of various neuronal proteins such as internexin neuronal intermediate filament protein, alpha b, synaptic vesicle membrane protein VAT-1 homolog, syntaxin binding protein 1a, internexin neuronal intermediate filament protein alpha a, synaptotagmin binding, cytoplasmic RNA-interacting protein, glial fibrillary acidic protein, myelin expression factor 2, synaptotagmin binding, cytoplasmic RNA interacting protein, myelin expression factor 2, in larvae	(Chen et al. (27)
BPA (2 and 20 μg/L)	Adults (F0)	4 months	F1 larvae (10 dpf), derived from F0 parents	1. The expression of mbp and syn2a proteins significantly decreased in a concentration-dependent manner. 2. The expression of α1-tubulin remained unaltered	(Guo et al. (28)
TiO2 (100 μg/L)	Adults (F0)	4 months	F1 larvae (10 dpf), derived from F1 parents	1. Significant reduction in the expression of mbp, syn2a, and α1-tubulin	(Guo et al. (28)
BPA (2 and 20 μg/L) + titanium (100 μg/L)	Adults (F0)	4 months	F1 larvae (10 dpf), derived from F1 parents	1. Significant concentration-dependent reduction in the expression of mbp. syn2a, and α1-tubulin	(Guo et al. (28)
TDCPP (4, 20, 100 μg/L)	Adults (4 months old)	3 months	F1 Larvae (5 and 10 dpf), derived from F1 parents	1. Significant concentration-dependent reduction in the expression of mbp, α1-tubulin, and syn2a was observed in larvae (5 and 10 dpf)	(Wang et al. (31)

Further, these 14 EDCs can also be classified as heavy metals (Cd, Pb, and Ti), brominated compounds (mBDE-47 and BDE-209), biocides (ATZ, TBT, and THM), flame retardants (OBS, TCEP, and TDCPP), plastic components (BPA and BPS), and sex steroids (E1). Moreover, seven of these chemicals (6-OH-BDE-47, ATZ, BPS, E1, OBS, TCEP, THM) were used during embryo-larval development, and six of them are in adults (BDE-209, Cd, Pb, TBT, TDCPP, TiO_2_), and one chemical (BPA) was studied on both embryos and adults (BPA). The transgenerational effects were studied in chemicals as binary (BDE209 and Pb, BPA and Ti, and TBT and Cd) and single (TDCPP, BPS, and OBS) exposures. Binary exposure experiments (exposed to two chemicals) were made exclusively on adult fish (ST-1).

During the experiment, zebrafish embryos from the wild type AB strain were used for ATZ, BPA, E1, mBDE-47, THM, and OBS as single chemicals (**Table 2A**). In two studies, TECP and BPS, embryos of the Tubingen (Tu) strain were used. In adult experiments, only AB-strain zebrafish were used; TDCPP was used as a single chemical (31), TBT and Cd, and BPA and TiO_2_ as a binary mixture (Guo et al., 2009). Two studies, one in embryos (OBS as a single exposure) and another in adults (BDE209 and Pb), did not mention the stain of the zebrafish in the published article (27, 38).

The duration of exposure also varied widely (**Table 2A**). Embryos were exposed during development (in *ovo*) until they hatched and were evaluated either immediately (mBDE-47, BPA, E1, TCEP, THM) or allowed for a significant time period for recovery before evaluation (ATZ). In case of BPS, the treatment is continued even after hatching (2 hpf-120 dpf) and evaluated immediately in adults as F0 or in eggs and larvae as F1 (35). For OBS, treatment was continued from 2 hpf to 21 days, and the adults (F0) were evaluated 180 dpf (depurated in a treatment-free condition 21–180 dpf) and in F1 larvae (7 dpf; the eggs and larvae were also maintained in treatment-free conditions). The adult fish were exposed for a required period of time (3–4 months) and evaluated immediately after the completion of the desired treatment (parental exposure as F0) and the eggs (F1) and larvae (F1; 5–10 dpf) were assessed after maintaining them in treatment-free conditions (BDE-209, Pb, BPA, Ti, TDCPP). Moreover, Li and Li (32) studied the effects of TBT and Cd by continuously exposing the adult fish for 90 days (F0), F1 larvae derived from F0 parents for 7 dpf (0–7 dpf); continued treatment for 5 months (F1 adults); and F2 as larvae (derived from F1 parents) for 7 dpf (**Table 2A**; **Supplementary Table 1**).

### 3.1 Effects of EDCs during embryo-larval development of zebrafish

The EDCs, ATZ, BPA, E1, mBDE-47, OBS, and THM were used during the embryonic development of zebrafish using the wild type AB strain; however, the EDCs, TECP, and BPS were used on embryos of the Tubingen (Tu) strain (**Table 2A**). Among these chemicals, ATZ is a biocide, BDE-47 is the brominated compound, BPA and BPS are plastic components, E1 is a steroid hormone, and OBS and TCEP are flame retardants (**Table 2B**).

#### 3.1.1 Atrazine

Atrazine (2-chloro-4-ethylamino-6-isopropylamino-s-triazine; ATZ) is a common water-soluble agricultural chlorinated herbicide and can leach from fields into surface and groundwater sources where it can persist in the environment (39–42). In the USA, it is the second most used herbicide, and the allowable maximum contamination level in the drinking water is 3 µg/L (43), while the European Union (EU), because of environmental persistence and groundwater contamination, banned ATZ in 2003 (44). As an EDC, ATZ induced abnormal metamorphosis and feminization in amphibians (45–47); disrupted the HPG and HPA axes in rodents (48–54); altered neurotransmission in rodents and zebrafish (55–59); caused reproductive abnormalities, including decreased semen quality in men (60); demasculinized and feminized male rats; and disrupted ovarian functions in female rats (48), also increased the risks of breast cancer in women (61). The mechanism of ATZ-induced endocrine disruption appears to be related to disrupted intracellular signaling through the inhibition of type 4 cyclic nucleotide phosphodiesterase (PDE4), increased cAMP levels, and decreased expression of steroidogenic proteins (62–65). ATZ exposure is also induced epigenetic changes in gene methylation and microRNA activity (66–68).

Our literature search found two manuscripts (29, 34) that studied the effects of ATZ on zebrafish after exposing them during embryonic larval development and evaluated neurobehavioral and epigenetic alterations in adults (**Table 2A**).

Zebrafish embryos were exposed to 0.3, 3, and 30 µg/L of ATZ for 72 hpf (1–72 hpf) and evaluated after 9, 12, and 14 months postfertilization (mpf). It was observed that the locomotor activity of the ATZ-exposed male fish significantly decreased at 9 mpf and the gene expression in nervous and reproductive system development was altered at 9, 12, and 14 mpf, and the raphe cell population decreased in the brain (29, 34). While global gene methylation analysis and brain weight during 12 and 14 mpf did not show any significant difference with controls (**Table 3**), transcriptomic analysis of the brain from 9 mpf male fish identified 123 genes with altered expression in 0.3 μg/L, 95 genes with 3 μg/L, and 121 genes with 30 μg/L. In females, at 9 mpf, hypomethylation in estrogen receptor signaling and hypermethylation in androgen signaling were observed in promoter regions, while in gene bodies, such variation was observed in genes, primarily enriched for mitochondria-related pathways (34). Gene expression analysis identified six genes that were altered in adult male brains with all three concentrations of ATZ; among them, three genes are upregulated (*ifgbp7*, *itm2cb*, and *sult2bl*), and three genes are downregulated (*aqp1a*, *cdk5*, and *cyp26b1*) (29). These studies suggested that ATZ, as a potential ED, targets the neurodevelopment of zebrafish embryos by epigenetic modification and modulation of behavior in adult stages.

#### 3.1.2 BPA

Bisphenol A (BPA) is widely used in the manufacture of myriads of commercial products, such as dental sealants, food packaging, water containers, baby bottles, and medical equipment (Hoekstra and Simoneau, 2013). Because of its persistent nature, contamination by BPA has been detected in various environmental matrices. In the aquatic environment, the level of BPA was found to be 28 μg/L (70). The ubiquitous presence of BPA in the environment exposed animals and humans to the continuous hazards associated with BPA (71–73). It is a well-known EDC and has the potential to impair reproductive potencies as well as the thyroid endocrine system by antagonizing the effects of TH receptors and also through several other mechanisms (74–80). Our literature search found two manuscripts that studied the effects of BPA on zebrafish embryos (36) and adults (28). Moreover, the effects of BPA in adult fish were studied as a combined exposure of BPA and TiO_2_ (28).

Zebrafish embryos were exposed to BPA (1, 10, 100, 1,000, and 10,000 nM; 0.223-2282.9 μg/L) at two different periods of development, 0–5 dpf (embryo-larval), which is considered an extended duration, and the 4–5 dpf (larvae) exposure was considered short-term exposure (36). Although compared with controls, BPA exposure (0–5 dpf) induced significant concentration-dependent skeletal abnormalities (significant only in fish exposed to 10,000 nM BPA) and total abnormalities (developmental abnormalities observed during embryo-larval development other than heart edema, skeletal abnormalities, yolk sac edema, and uninflated swim bladder) (significantly different in 100 and 10,000 nM); however, other abnormalities such as improperly inflated swim bladder, yolk sac edema, and cardiac edema did not show any significant difference. Moreover, short-term exposure (4–5 days) of the larvae also did not show any significant difference in all these morphological parameters (skeletal and total abnormalities) with controls. In behavioral analysis, embryos exposed to BPA (0–5 dpf) showed concentration-dependent hyperactivity in the dark; however, short-term exposure (4–5 dpf) induced hypoactivity in a concentration-dependent manner. A total of 168 DEGs were expressed in embryos (0–5 dpf) when exposed to BPA, while 1296 DEGs were observed when the larvae (4 dpf) were exposed to BPA for 1 day. Moreover, 90 DEGs were common between the larvae either exposed to BPA during embryonic development (0–5 dpf) or in larvae for 24h (4–5 dpf) (**Table 6**). Among the 33 neurological genes investigated, 28 genes were differentially expressed after BPA exposure (36).

Among the neurologic genes, BPA upregulates *tgif1*, *tubg1*, *cat*, *cdk4*, *ndufb6*, *rps13*, *rpl35a*, *rack1*, *ndufv2*, *rho*, and downregulates *slc7a1a*, *crybb1*, *fat2*, *rho*, when exposed during development (0–5 dpf). However, exposure of the larvae (4 dpf) in BPA (100 and 1000 nm) only for 24h (4–5 dpf) upregulates *ttr*, *ndufb6*, *rps13*, *rpl35a*, *crybb1*, *rack1*, *ndufv2*, *ndufs4*, *id1*, *atp6ap2*, *dbi*, *rho*, and *rps19*, while downregulating *pparab*, *fat2*, *tshz2*, *dock3*, *wwc1*, *thi2a*, *dync1h1*, *tshz2*, *arhgef10*, *ubb*, and *plxna2*. The genes belong to endocrine pathways related to reproduction. BPA in 0–5 dpf exposure upregulates *prdx3* and *prdx4*, while in larval exposure (4–5 dpf) upregulates *txn2*, *txnipa*, *gstp1*, *prdx2*, *gpx1a*, and *gpx1b*, and downregulates *txnipa* and *txnipb* in larvae exposed to 1000 nm BPA. The endocrine pathway genes related to lipid metabolism, BPA (1 nm) upregulates *fads2*, and *apoc2* when exposed during embryonic development (0–5 dpf), while short-term exposure of the larvae (4–5 dpf) *fads2*, *apoa1a*, *apoa2*, *apoa4b.1*, *apoc2* and downregulates *abcg2a*, and *dnmt1* genes (36). Other endocrine pathway genes related to cholesterol metabolism, BPA (0–5 dpf) did not show any significant effect (36), while exposure of the larvae (4 dpf) to BPA for 24h, upregulate *cyp7a1*and downregulates *osbp*, *dhcr24*, and *atp8b1* genes at 100 nm concentration, while downregulates *cyp7b1*, *osbp*, and *dhcr24* genes at 1000 nm concentrations (36). Moreover, two endocrine pathway genes (*agpat5* and *pck1*) related to glucose metabolism were studied in zebrafish embryos (0–5 dpf) and larvae (4–5 dpf). BPA has no significant effect on the expression of any of these genes during embryonic exposure, while in larvae, only *agpat5* gene was downregulated in a concentration-dependent manner (36). Other than neurological and endocrine pathway genes, BPA has a significant effect on genes related to skeletal development (**Table 6**). Among the four genes studied (*tnfrsf1a*, *eif3a*, *utrn*, and *xylt1*), only *eif3f* was upregulated by BPA during embryonic development (0–5 dpf), while in larvae (4–5 dpf), BPA upregulates *eif3f* and downregulates *utrn* in a concentration-dependent manner. Moreover, *xylt1* downregulation in larvae was nonmonotonic, significant only in the 100 nm group, not in the 1000 nm group (**Table 6**).

#### 3.1.3 BPS

BPS is a synthetic organic compound used as a substitute for BPA. It has been widely used in personal care products, thermal printing papers, and the production of polycarbonate plastics and epoxy resins (81, 82). BPS has higher heat stability; it is frequently detected in various environments, including water (83, 84). BPS was also detected in human urine with a concentration range of 0.02–21 ng/ml (85). It is an endocrine disruptor and has adverse effects on the reproductive system and nervous system in animals and humans (86). Zebrafish embryos, exposed to BPS for 7–75 days during development, altered whole body T3 and T4 levels and the expression of hypothalamus-pituitary-thyroid axis (HPT-axis) genes (87, 88).

Our literature search found only one manuscript that studied the effects of BPS as an EDC that modulates nervous system development in zebrafish (35). Embryos (2 hpf) of zebrafish (Tubingen strain) were exposed (2 hpf–120 dpf) to BPS (1, 10, and 100 μg/L), and the blood plasma of the parents (F0) was analyzed for thyroid hormone levels (T3 and T4) and also used for breeding in a treatment-free environment (no BPS) to obtain F1 eggs and larvae (96 hpf) for further analysis.

In adult (F0) females, compared with control fish, the plasma level of T4 was significantly decreased after BPS exposure (1, 10, and 100 μg/L), while in males (F0), the plasma T4 levels remained unaltered (**Table 4**). In contrast to T4, plasma T3 levels in females were significantly increased in fish by BPS (1, 10, and 100 μg/L); however, in males, a significant increase was observed in lower concentrations (1 and 10 μg/L) but not in the highest concentration (100 μg/L) of BPS used in this study (**Table 4**; **Supplementary Table 1**).

In F1 eggs, derived from F0 parents, hatching was significantly delayed. The head-trunk angles (HTA) were also significantly reduced, and the length of otic vesicles significantly increased in embryos (30 hpf) when compared with controls (**Table 3**; **Supplementary Table 1**). Moreover, T4 concentration was significantly reduced, and T3 concentrations were significantly increased in F1 eggs (**Table 4**). The spontaneous movements (touch-evoked response) during 48 hpf were significantly reduced in embryos (**Table 5**). The expression of *zhe1* gene (encoding the zebrafish hatching enzyme) was significantly reduced in F1 eggs (**Table 6**).

In larvae, compared with controls, the swim bladder inflation was decreased in the F1 generation derived from the parental exposure to BPS (1, 10, or 100 μg/L for 120 days). Consequently, the number (concentration-dependent) and area (in all concentrations) of lateral stripe melanocytes were significantly reduced in larvae. Moreover, the average swimming speed and the swirl-escape rate were also significantly reduced in F1 larvae (96 hpf) (**Table 5**; **Supplementary Table 1**). Furthermore, a significant concentration-dependent reduction in the expression of surfactant protein genes (*sp-a*, sp*-b*, and *sp-c*) was observed in F1 larvae (96 hpf). Compared with controls, a concentration-dependent significant upregulation in the expression of *gfap*, *gap43*, and *mbp*, and downregulation of *syn2a* mRNA and a concentration-dependent significant reduction in the expression of *crestin* and *tyr* genes were observed in larvae (96 hpf) derived from the parents exposed to BPS for 120 days (**Table 6**).

#### 3.1.4 E1

Estrone (E1), an estrogen and a sex steroid hormone primarily produced after menopause in women and responsible for sexual development, is naturally produced by the ovaries, adipose tissues, and adrenal glands. Postmenopausal women undergoing hormone therapy are typically prescribed estrone or estradiol, and estrogen can be converted to estrone and excreted in the urine and fecal matter (89). It is one of three naturally occurring estrogens, alongside estradiol (E2) and estriol (E3). E1 plays a role in female sexual development and function, and low or high levels can lead to various symptoms. It is also used in menopausal hormone therapy. E1 is present in the environment, coming from human and animal excretion and improper removal by wastewater management. The estrone are frequently detected in surface water mainly because of human usage or waste (76). It is considered an emerging pollutant, especially in water and soil, and can have adverse effects on both humans and wildlife (90).

Our search strategy found only one article where the effects of E1 in zebrafish embryos (0–5 dpf, extended duration) and larvae (4–5 dpf, short-term exposure) were evaluated and compared with the effects observed by BPA in identical conditions of exposure (36). The embryos were exposed to E1 (0.01, 0.1, 1, 10, and 100 nm or 0.027–27.03 μg/L) during development (0–5 dpf) and screened for mortality and total abnormalities (unhatched embryos, skeletal deformities, improperly inflated swim bladder, yolk sac edema, and heart edema) (36). Although the calculated total abnormalities were not significantly different from controls, E1 exposure concentrations of 1–100 nm had higher skeletal abnormalities and uninflated swim bladders than lower E1 concentrations (0.1 nm) and also than controls (**Table 3**; **Supplementary Table 1**). The exposure of the larvae (4 dpf) for 24h also showed similar effects of E1 on morphological abnormalities as observed in embryos (**Table 3**; **Supplementary Table 1**). Embryonic exposure to E1 (0–5 dpf) led to concentration-dependent skeletal abnormalities and an uninflated swim bladder, and the calculated total abnormalities are not significantly different from controls. The larvae (5 dpf) treated with E1 during development (0–5 dpf or 24h only 4–5 dpf) become hypoactive in the dark, which is concentration dependent (**Table 5**). Moreover, a total of 445 differentially expressed genes (DEGs) were observed after E1 exposure during embryonic development (0–5 dpf); however, a total of 83 DEGs were observed when the larvae (4 dpf) were exposed to E1 for 24h (**Supplementary Table 1**). Moreover, the genes related to neurological development, E1 exposure during development (0–5 dpf) significantly upregulates *apoea*, *sigmar1*, *fbl* and downregulated *ttr*, *enox1*, *ddc* (**Table 6**; **Supplementary Table 1**). Among the genes related to reproduction, E1 exposure to embryos (0–5 dpf) upregulated *gemin 5*, *atf3*, and *txn*, and downregulated *fabp6*, and t*xnipa*. The genes related to lipid metabolism, E1 exposure upregulated *elovl5* and downregulated *fads2*, *elovl2*, and *lpin1* in the embryos (0–5 dpf exposure). The genes related to cholesterol metabolism, developmental exposure (0–5 dpf) downregulated *cyp7a1* gene. Moreover, two genes (*agpat5* and *pck1*) related to glucose metabolism were studied in zebrafish embryos (0–5 dpf) and larvae (4–5 dpf). E1 downregulated *pck1* during embryonic development (0–5 dpf). E1 has a significant effect on skeletal development. Among the four genes studied (*tnfrsf1a*, *eif3a*, *utrn*, *xylt1*), only *tnfrst1a* was upregulated in embryos when exposed to E1.

#### 3.1.5 mBDE-47

The chemical 6-OH-BDE -47 (mBDE-47) is a hydroxyl-metabolite of BDE-47 (a flame retardant) showed stronger binding affinity to transthyretin (TTR) and thyroxine-binding globulin (TBG) the crucial plasma proteins that transport T3 and T4 and stabilized TH levels in the blood. Alterations in TTR or TBG typically result in a euthyroid phenotype (91). mBDE-47 also disrupts Ca^2+^ homeostasis and neurotransmitter release in PC12 cells (92). Our literature search found only one article that studied the effects mBDE-47 as an EDC targeting neurobehavioral development in zebrafish (33). Embryos of zebrafish were exposed to mBDE-47 (1, 10, 50, and 100 nM [0.5018–50.17 µg/L]; 4–96 hpf) and their coiling frequency (22, 26, 30, and 34 hpf) was evaluated during development (33). It was observed that in control embryos, the coiling frequency was found to be highest at 22 hpf and then gradually reduced over time and found to be minimal at 34 hpf. Compared with control embryos, mBDE-47 exposure significantly delayed the reduction in coiling frequency in a concentration and time-dependent manner. Injection of thyroid hormone receptor β (*thrβ*) mRNA to the embryos during 4 hpf and evaluating the coiling frequency during 26 hpf, indicated that *thrβ* mRNA partially rescued the embryos from the toxic effects (disrupting coiling frequency) of mBDE-47. Moreover, in the brain (26 dpf), a significant increase in apoptotic neurons induced by mBDE-47 exposure (26 hpf) was also partially rescued by injection of *thrβ* mRNA. The expression of *tph2* mRNA (crucial enzyme for serotonin synthesis, specifically in the raphe nuclei of the brain) was significantly downregulated in a concentration-dependent manner (100 nM) in the embryos (26 hpf) exposed to mBDE-47. Moreover, the number of 5-hydroxytryptamine (5-HT, serotonin) immunoreactive neurons in the hypothalamus was significantly reduced after mBDE-47 exposure as observed in larvae (96 hpf). Therefore, coiling behavior disorder, induced by mBDE-47 could potentially be induced due to the disruption of thyroid hormone reserves in the embryos and be used as an indicator of neurobehavioral toxicity in zebrafish development.

#### 3.1.6 Thifluzamide

THM is an organofluoride with a broad spectrum and a great potent succinate dehydrogenase inhibitor (SDHI) fungicide (93) and is widely used to control sheath blight, a fungal disease of rice caused by *Rhizoctonia solani*. Because of its wide application in agriculture, THM is easily transported into the aquatic environments (94, 95). Our search strategy found only one article on zebrafish where THM showed significant EDC and neurotoxic effects (37).

Zebrafish embryos (wild type AB strain) were exposed to THM (0.19, 1.90, and 2.85 μg/L) for 6 days and evaluated the toxicity (hatching, swimming behavior), and neuroendocrine effects during embryo-larval development (37). It was observed that THM exposure induced significant hatching delay, decreased larval length, induced spinal curvature, and neurobehavioral disorders during embryo-larval development (**Tables 3**, **5**; **Supplementary Table ST-1**). Although TRH and TSH levels in all THM exposed embryos/larvae (96 hpf) did not show any significant changes, thyroid hormone concentrations (T3 and T4) showed significant alterations (**Table 4**). The neurotransmitter contents such as 5-HT, and norepinephrine (NE) significantly increased in a concentration-dependent (5-HT) or independent (NE) manner and the AChE activity tended to decrease with the increase in THM concentrations (significant with 2.85 μg/L) (**Table 5**). Several genes related to nervous system development and functions were either up- (*flk, neurog1*, and *vegf*), or down-(*ldha* and *aidh2.1*)-regulated, or remained unaltered (*angpt1*, *ascl1a*, *eif1b*, *elavl3*, and *left1*) in larvae by THM exposed during embryo-larval development. The HPT axis genes (*pax8*, *hhex*, *tshγ*, *tshβ*, *thrα (trα)*, *thrβ (trβ)*, *ahr2*, *cyp1a*, *ugtlab*, *dio1*, *dio2*, *nkx2.1*, *tg*, *crh*, and *ttr*) were upregulated by THM exposure, even though the expression was sometimes nonlinear (**Table 6**). Therefore, THM disrupts TH concentrations in zebrafish embryos, probably by decreasing the maternal TH reserves during embryo-larval development.

#### 3.1.7 Sodium *p*-perfluorous nonenoxybenzene sulfonate

The sodium *p*-perfluorous nonenoxybenzene sulfonate (OBS), an alternative to perflurooctane sulfonate (PFOS), has been widely used in various industrial applications, such as fire protection, steel plant cleaning, printing, and electroplating industries (96). Therefore, OBS has possibilities of releasing to the aquatic environments, including rivers, lakes, drinking water, ground water, sea water, and wastewater (97–99). Although extensive studies on the toxicity of PFOS have been made on fish, only a few studies have been performed on OBS (100–102). It was observed that OBS has been accumulated in the liver, kidney, gill, and blood of crucian carp (fish) and its potential toxic effects in aquatic animals (103, 104). Our literature search strategy found only one article that showed the potential impact of OBS on neurological disorders induced in zebrafish (38).

Embryos of zebrafish (2 hpf) were exposed to OBS (3, 30, and 300 μg/L) for 21 days and then depurated and grown in OBS-free medium for an additional 159 days (total 180 days) to get F0 adults. The blood, brain, and liver tissues of F0 adults were collected for analysis, and the head-trunk region was used for studying thyroid histopathology. Moreover, some of the F0 adults (both males and females) are allowed for breeding. The eggs (F1) within 2 hpf were analyzed for TH (T3 and T4) concentrations. The developmental abnormalities (survival rates, malformations [pericardial edema, yolk sac edema, eye defects, spinal curvature, and tail curvature], hatching rates, heart rates, body length and body weight) during embryo-larval development was observed until 7 dpf (F1) and then the larvae (F1) were used for behavioral analysis and gene expressions related to HPT axis (38).

It was observed that in F0 adults, exposure to OBS during early developmental stages induced a sex-specific effect targeting the HPT-axis of the fish. Compared to control fish, the histopathological evaluation of the thyroid glands did not show any significant difference in the average colloid area in thyroid glands in both male and female fish; however, the thickening of the follicular area and depletion of the colloid were observed in experimental fish exposed to OBS during the early period of development (**Table 3**; **Supplementary Table 1**). Moreover, the height of epithelial cells in the thyroid gland of both male and female fish was significantly increased after OBS exposure during early life stages. T3 levels in plasma of both male and female fish showed non-monotonic enhancements when exposed to lower concentrations of OBS during early life stages (3 μg/L in females and 3 and 30 μg/L in males) of development. T4 levels showed sex-specific alterations (in females showed significant concentration-dependent decrease and in males, significant concentration-dependent increase) when OBS exposure was made during early life stages (**Table 4**). Gene expression analysis indicated that the expression of the *crh* gene was enhanced in a concentration-dependent manner (300 μg/L) in females (F0), and the expression of *tshβ* tended to decrease (not significant) in female fish brain exposed to OBS during early development, while in male brain, the transcription of *crh* and *tshβ* remained unaltered. The expression of *tshr* remained unaltered in the female brain, while downregulated only in the lower concentration (3μg/L) in the male brain in F0 fish exposed to OBS during early development (2 hpf–21 dpf). Compared with control fish, the expression of *tpo* was upregulated in the female brain in a nonmonotonic fashion, while in males, both *tg* and *tpo* were downregulated in a concentration-dependent manner. As a result, the expression of *trα* was significantly downregulated in male brains and *trβ* was upregulated in female brains. The expression of *ttr* was significantly upregulated in the liver of female fish in a concentration-dependent manner, while in males, no effects on *ttr* of liver were observed. The expression of *dio2* in the liver of female fish and *dio1* and *dio2* in the liver of male fish was downregulated in a concentration-dependent manner. The expression of *ugt1ab* in the liver of female fish was downregulated whereas, in the male fish it was upregulated by OBS exposure (**Table 6**).

In F1, compared with controls, the hatching, malformation, survival, and the lengths and weight of the hatched embryos (7 dpf) during embryo-larval development did not show any significant difference after the parental exposure to OBS during early life stages (**Table 3**; **Supplementary Table 1**). In F1 eggs (2 hpf), the T3 contents showed a significant increase while T4 levels remained unaltered (**Table 4**; **Supplementary Table 1**). Moreover. In F1 larvae (7 dph), compared with controls, the T3 content in the whole larvae (7 dpf) was significantly decreased in lower concentration (3 μg/L) and increased in higher concentrations of OBS groups (30 and 300 µg/L), whereas T4 content decreased significantly (**Table 4**; **Supplementary Table 1**). The swimming behavior (swirl-escape rate) of F1 larvae significantly decreased in a concentration-dependent manner due to parental exposure to OBS in early developmental stages (**Table 5**; **Supplementary Table 1**). The gene expression analysis indicates that parental exposure to OBS during early life stages significantly decreased the expression of *tshβ*, *tg*, and *nkx2.1* compared with control larvae. The expression of *syn2a* and *mbp* was upregulated when the parents were exposed to higher OBS concentration (300 μg/L) during early developmental stages.

#### 3.1.8 TCEP

Tris-(2-chloroethyl) phosphate (TCEP) is an organophosphorus flame retardant (OPFR) widely used in plastics, building materials, electronic devices, furniture, baby toys, and other products. TCEP is not covalently bound to the polymer matrices and tends to be released into the environment in many pathways, such as volatilization, leaching, and abrasion (105). Therefore, high concentrations of TCEP have been detected in various environments, including surface water, atmosphere, street dust, soil, sediments, blood of birds and humans (106–111). It is highly water soluble and therefore availability in the aquatic system is extremely high (112, 113).

Our search strategy found only one article that showed potential endocrinological and neurodevelopmental disorders induced by TCEP in zebrafish. Embryos of zebrafish (Tu strain) were exposed to TCEP (0.2, 2, 20, and 200 μg/L) during embryo-larval development and evaluated toxicological effects, neurobehavioral disorders, and thyroid endocrine disrupting effects induced by the chemical (TCEP) during 48–120 hpf of development (30). Significant development-specific reduction in heartbeats was observed in embryos (200 μg/L; 48 hpf) exposed to TCEP during development. Although not significant, a concentration-dependent inhibition in hatching rates (hatching delay) of the embryos (20 and 200 μg/L) was observed only during 72 hpf. Moreover, the length of the larvae was found to be reduced in a concentration-dependent manner (20–200 μg/L) in 72 and 120 hpf of development, even though the larval malformations (yolk sac edema, tail deformation, bent spine) induced by different concentrations of TCEP was not significantly different from the controls during embryo-larval development (**Table 3**; **Supplementary Table 1**). T3 levels of whole larvae (120 hpf) did not show any significant difference with controls, however, T4 levels significantly reduced after TCEP exposure in a concentration-dependent manner (**Table 4**; **Supplementary Table 1**).

Gene expression analysis related to thyroid endocrine disruptions in 120 hpf larvae indicated that expression of *nis (slc5a5)* and *trα* was significantly upregulated in embryos exposed to 2, 20, and 200 μg/L of TCEP (**Table 6**; **Supplementary Table 1**). Moreover, the expression of *trβ* and *ugt1ab* was also increased in a concentration-dependent manner (2 and 20 μg/L). However, the expression of *tg* and *tshr* was downregulated in larvae developmentally exposed to TCEP (0.2–200 μg/L). The expression of *α1-tubulin* was downregulated, whereas *gap43* and *mbp* were significantly upregulated in larvae (120 hpf) after exposure to TCEP (0.2, 2, 20, and 200 μg/L). Upregulation also occurred in *syn2a* mRNA in larvae exposed only to 200 μg/L (concentration dependent) during development. Moreover, the expression of *elavl3* was enhanced only in larvae exposed to 20 μg/L and downregulated in larvae exposed to higher concentrations of TCEP (200 μg/L) during development (**Table 6**).

### 3.2 Parental and transgenerational effects of EDCs on zebrafish

Despite the studies of neurodevelopmental disorders during embryo-larval development of zebrafish induced by EDCs, reproductively active adults with fully functional endocrine and nervous systems were used for other EDCs (BDE-209, BPA, Cd, Pb, TBT, TDCPP, TiO2), which were not studied in the zebrafish embryos (except BPA). Among these seven EDCs, three are metals (Cd, Pb, and Ti). Moreover, these metals were also studied as binary mixtures (BDE-209 and Pb, BPA and TiO2, and TBT and CD). The transgenerational effects were also studied in all seven chemicals as single (BDE-209, BPA, Cd, Pb, TBT, TDCPP, and Ti) and in six EDCs as binary mixtures (BDE-209 and Pb, BPA and Ti, and TBT and Cd).

#### 3.2.1 BDE-209

Polybrominated diphenyl ethers (PBDEs) are widely used as additive flame retardants in electronic devices and are released in significant amounts into the environment during the recycling of e-waste (114). Among them, decabromodiphenyl ether (BDE-209) is detected predominantly following the phasing out of lower brominated congeners (115, 116). BDE-209 can be bioaccumulated in various biological samples (115), and it is identified as a neurotoxicant because of its adverse effects on locomotor behavior (117), neuronal protein levels (118, 119), and neuronal apoptosis (120, 121). Our search strategy found only one article studied the effects of BDE-209 either alone or in combination with Pb on adults (**Table 2A**).

Zebrafish adults (both males and females) were exposed to BDE-209 for 3 months and did not show any change in the length and weight of the fish; however, the condition factor [(weight/Length ^3^) × 100] was inconsistently increased in fish exposed to 1 and 100 μg/L BDE-209 alone (**Table 3**; **Supplementary Table 1**). Coexposure of male fish with BDE-209 and Pb (10 μg/L), significantly decreased the body weight of male fish exposed to 1μg/L BDE 209+Pb and increased both length and weight of the fish exposed to 100 μg/L BDE-209+ Pb; however, the condition factor was found to be significantly decreased only in fish exposed to 1 μg/L BDE-209+Pb. In females, exposure to BDE-209 did not show any significant change in the length and weight of the fish; however, the length of the fish exposed to 1 μg/L BDE-209, showed significant increase when compared with controls, though the condition factor did not show any significant change with control fish when exposed to BDE-209 (1, 10, and 100 μg/L) either alone or in combination with Pb (10 μg/L).

The hepatosomatic index [HSI (liver weight/Body weight)X100) in male fish remained unaltered by BDE-209 (1, 10, and 100 μg/L) either alone or in coexposure with Pb (10 μg/L); however, in females, HSI significantly decreased in fish exposed to 1 μg/L BDE-209+Pb and increased in fish exposed to 100 μg/L+ Pb. The brain-somatic index [BSI = (weight of brain/body weight) × 100] did not show any significant change in male fish exposed to BDE-209 (1, 10, and 100 μg/L) either alone or in combinations with Pb (10 μg/L); however, females exposed to BDE-209 (100 μg/L) showed significant increase in BSI when compared with controls and coexposure with Pb showed significant decrease when compared with the fish exposed to 100 μg/L BDE-209 alone. The GSI significantly decreased in male fish exposed only to BDE-209 (10 μg/L) alone; coexposure with Pb significantly decreased GSI when compared with BDE-209 (10 and 100 μg/L) alone (**Table 3**). In females, GSI remained unaltered when exposed to BDE-209 either alone or in combinations with Pb; however, a significant increase in GSI was observed when females were exposed to BDE-209 (10 μg/L)+ Pb. No significant effect was observed on fecundity (number of eggs/female/day); however, a significant reduction in fecundity was observed when the parents were coexposed to 100 μg/L of BDE-209 and Pb (10 μg/L) (**Table 3**; **Supplementary Table 1**).

Significant concentration-dependent accumulation of PBDE (total) was found in F1 eggs derived from the parental exposure of BDE-209, and the presence of Pb in the environment further increased the concentration of PBDE in the eggs (**Table 3**; **Supplementary Table 1**). Moreover, the size of the eggs remained unaltered by parental exposure to BDE-209 alone; however, a significant increase in the egg size was observed when the parents were coexposed in a combination of BDE-209 (1 μg/L) and Pb (10 μg/L). BDE-209 alone has no significant effect on hatching of the embryos, while Pb either alone (10 μg/L) or in combination with BDE-209 (10 and 100 μg/L) significantly delayed hatching compared with control embryos. In larvae (F1), the length and weight remained unaltered when the parents were exposed to BDE-209 (1, 10, and 100 μg/L) and Pb (10 μg/L) either alone or in combination (**Table 3**).

The circulating TH levels in the adult fish serum (both males and females) were also investigated (**Table 4**). It was observed that although serum T3 level was significantly increased in a concentration-dependent manner in male fish exposed to BDE-209 (100 μg/L) either alone or in combination with Pb (10 μg/L), no significant alteration in the serum T4 levels of male fish exposed to BDE-209 alone or in combination with Pb was observed. However, in males, BDE-209 at 10 μg/L significantly increased serum T4 level when coexposed with Pb (10 μg/L). In females, compared with controls, serum T3 level significantly decreased when exposed to BDE-209 alone; coexposure with Pb (10 μg/L) significantly decrease serum T3 levels in females when BDE-209 concentrations are 1 and 10 μg/L. Serum T4 contents in females remained unaltered in fish exposed to lower concentrations of BDE-209 (1 and 10 μg/L) and significantly decreased in females exposed to BDE-209 (100 μg/L) alone. Presence of lead (10 μg/L) either alone or in combination with BDE-209 (1 and 100 μg/L) significantly decreased serum T4 levels in adult fish when compared with controls. T3 content significantly decreased in eggs (F1) derived from parents exposed to 1μg/L BDE-209 and remained unaltered in eggs derived from fish exposed to higher concentrations of BDE-209 (10 and 100 μg/L); T3 content in eggs derived from fish exposed to BDE-209 (10 and 100 μg/L) and Pb (10 μg/L) significantly reduced when compared with control eggs (F1). Moreover, T4 contents in eggs (F1) derived from parents exposed to BDE-209 (1, 10, and 100 μg/L) alone remained unaltered, while significantly reduced in eggs derived from the fish coexposed with Pb (10 μg/L) and BDE-209 (10 and 100 μg/L). T3 levels of larvae derived from parents exposed to BDE-209 alone (1 and 10 μg/L) remained unaltered and significantly increased in larvae derived from parents exposed to BDE-209 (100 μg/L) alone. T3 contents significantly increased in larvae derived from parents coexposed with lead (10 μg/L) and BDE-209 (10 and 100 μg/L). T4 contents in larvae derived from parents exposed to BDE-209 (1, 10, and 100 μg/L) alone remained unaltered; while a significant increase was observed in larvae derived from parents coexposed with BDE-209 (1, 10, and 100 μg/L) and Pb (10 μg/L).

The serum testosterone (T) levels in males (F0) remained unaltered by BDF-209 (1, 10, and 100 μg/L) or Pb (10 μg/L) exposure. However, coexposure of BDE-209 with lead significantly reduced serum T levels in male fish. In females, both BDE-209 (1, 10, and 100 μg/L) and Pb (10 μg/L) either alone or in combinations was able to significantly reduce serum T levels when compared with control fish. The serum E2 levels in male fish remained unaltered when the fish were exposed to lower concentrations of BDE-209 (1 and 10 μg/L) or Pb (10 μg/L) alone, while a higher concentration of BDE-209 alone (100 μg/L) significantly enhanced serum E2 levels in male fish. Moreover, combined exposure of Pb (10 μg/L) with all concentrations of BDE-209 used in this study (1, 10, and 100 μg/L), significantly reduced serum E2 levels in male fish when compared with the fish exposed to corresponding BDE-209 alone. In females, serum E2 levels significantly decreased by BDE-209 (10 and 100 μg/L) and Pb (10 μg/L) alone. Moreover, a significant reduction in serum E2 levels was also observed in fish (females) in combined exposure conditions (BDE-209+Pb). In F1 eggs derived from parents exposed to BDE-209 (1, 10, and 100 μg/L) alone, the concentration of T was significantly reduced when compared with corresponding control eggs. Eggs derived from parents coexposed with BDE-209 (1 and 10 μg/L) and Pb (10 μg/L) showed a significant reduction, while a significant enhancement was observed in the T content of eggs derived from parents exposed to BDE-209 (100 μg/L) in combination with Pb (10 μg/L). E2 content was enhanced in eggs derived from the parents exposed to 10 and 100 μg/L BDE-209 alone. Moreover, eggs derived from parents exposed to Pb either alone (10 μg/L) or in combinations with BDE-209 (1 and 10 μg/L+ Pb) did not show any significant effect. However, significant enhancement in the E2 content was observed in eggs derived from the parents exposed to BDE 209 (100 μg/L) and Pb (10 μg/L) in combinations. The T contents in larvae derived from the parents exposed either to BDE-209 (1, 10, and 100 μg/L) or Pb (10 μg) alone or in combination showed significant reduction when compared with controls. The E2 content in larvae derived from the parents exposed to BDE-209 (10 and 100 μg/L) either alone or in combination with Pb (10 μg/L) significantly reduced when compared with controls. Moreover, the E2 content in larvae derived from parents exposed with Pb (10 μg/L) alone did not show any significant change, while significantly reduced in larvae when the parents were exposed in combinations (1 μg/L BDE-209+Pb). Downregulation of the thyroid hormone receptor protein 3b was observed in larvae derived from parents exposed to BDE-209 (1 μg/L) and Pb (10 μg/L) either alone or in combinations (**Table 7**). Proteomic analysis of the larvae derived from parents exposed to BDE-209 (1 μg/L) either alone or in combination with Pb (10 μg/L) did not induce any significant change in the vitellogenin content of the larvae, while Pb (10 μg/g) alone was able to show universal over expression of vitellogenin. Moreover, downregulation of cathepsin S by BDE-209 and Pb either alone or in combination was observed. The neural protein syn2a was decreased significantly in a concentration-dependent manner in larvae derived from the parents exposed to BDE-209 (significant in 10 and 100 μg/L) and Pb (10 μg/L) either alone or in combinations. The neural protein mbp was decreased significantly in a concentration-dependent manner in larvae derived from the parents exposed to BDE-209 (1, 10, and 100 μg/L) and Pb (10 μg/L) either alone or in combinations. Proteomic analysis found increased and decreased levels of various crystalline proteins, muscle proteins, in larvae derived from parents exposed to BDE-209 (1 μg/L) and Pb (10 μg/L) either alone or in combinations. Moreover, proteomic analysis also found increased and decreased levels of various neuronal proteins such as internexin neuronal intermediate filament protein alpha b, synaptic vesicle membrane protein VAT-1 homolog, syntaxin binding protein 1a, internexin neuronal intermediate filament protein alpha a, synaptotagmin binding, cytoplasmic RNA interacting protein, glial fibrillary acidic protein, myelin expression factor 2, in larvae derived from parents exposed to BDE-209 (1 μg/L) and Pb (10 μg/L) either alone or in combinations. Various excitatory and inhibitory neurotransmitters, such as glutamate, GABA, serotonin, tryptophan, ACh, tyrosine, choline, 5-hydroxyindole acetic acid, dopamine, norepinephrine, epinephrine, 3,4-dihydroxy phenylacetic acid were altered in larvae derived from parents exposed to BDE-209 (1 μg/L) and Pb (10 μg/L) either alone or in combinations.

The protein analysis was restricted only to larvae of F1 generation after exposing the parents (F0) to the respective EDCs for 3 (BDE-209, Pb, TDCPP) and 4 (BPA and Ti) months. The breeding of the fish and the rearing of the larvae were made in a treatment-free medium.

#### 3.2.2 BPA

Zebrafish adults (AB strain 90 days old) were exposed to BPA (2 and 20 μg/L) either alone or in combination with TiO2 (100 μg/L) for 4 months and allowed breeding in a treatment-free (no BPA or TiO2) environment for obtaining F1 eggs and larvae. Moreover, the peripheral blood, liver, gonad, and brain of the parental fish (F0) were also analyzed. The development and growth of the F1 eggs and larvae were observed in control (no BPA or TiO2) conditions. The developmental abnormalities (survival, hatching, locomotion, and growth) in F1 eggs and the locomotor activity, thyroid hormone concentrations, neurotransmitter contents, and AChE activity were assessed in larvae (10 dpf).

It was observed that BPA was accumulated in F0 parents (both male and females) in a concentration-dependent manner, and the presence of TiO_2_ in the environment significantly enhanced the accumulation process (**Table 3**; **Supplementary Table 1**). Moreover, despite the accumulation of BPA, T4 levels in the blood of females were significantly decreased in a concentration-dependent manner, while in combined exposures, BPA together with TiO2 significantly reduced serum T4 levels in F0 parents (both males and females), also in a concentration-dependent manner. Plasma T3 levels remained unaltered in both F0 parents (male and female fish) after exposure to BPA alone, while in combined exposure, significant concentration-dependent reduction was observed in T3 levels in females and remained unaltered in males (**Table 4**). The neurotransmitter contents (serotonin, dopamine, and acetylcholine) and the AChE activity in the brain of the F0 parents remained unaltered; however, BPA in the presence of TiO_2_ significantly reduced the serotonin and dopamine contents of the brain of females and remained unaltered in males (**Table 5**). Also, the expression of *crh* and *tshβ* mRNAs were upregulated after BPA exposure (**Table 6**; **Supplementary Table 1**).

Like parents (F0 adults), BPA was accumulated in eggs (F1) due to the parental exposure to BPA either alone or in combination with TiO2. Moreover, accumulation of BPA was significantly higher and concentration-dependent when the parents were coexposed to a combination of BPA and TiO_2_ (**Table 3**). Similarly, a concentration-dependent reduction in the T4 contents of the F1 eggs was observed when the parents were exposed to BPA alone or in combination with TiO_2_ (**Table 4**). Moreover, as in parents, T3 contents of the F1 eggs did not show any significant changes with the controls when the parents were exposed either to BPA alone or in combination with TiO_2_ (**Table 4**). Hatching (3 dpf), malformation rates, and embryo-larval survivability were significantly reduced in a concentration dependent manner, when the parents (F0) were exposed to (2–20 μg/L) BPA. Moreover, combined exposure (BPA 20 μg/L+ TiO_2_ 100 μg/L) of parents, significantly reduced hatching (3 dpf), enhanced malformation rates, decreased survivability and body weight of the larvae (F1) compared with the larvae derived from the parents exposed either as controls or BPA alone (**Table 3**). Consequently, T4 content in the F1 larvae (10 dpf) decreased significantly in a concentration-dependent manner when parents were exposed for 4 months with BPA (2–20 μg/L) either alone or in combination with TiO_2_. The T3 level remained unaltered in all treatment groups, however, significantly reduced in larvae when parents (F0) were exposed to BPA (20 μg/L) and TiO_2_ (100 μg/L) together for 4 months (**Table 4**). The neurobehavioral analysis of the F1 larvae, indicates that the swimming speed in the dark phase and during light-dark transition, was reduced in a concentration-dependent manner (**Table 5**; **Supplementary Table 1**). The neurotransmitters, serotonin, and dopamine were significantly reduced in a concentration-dependent manner (BPA alone or in combination with TiO_2_), while no effect was observed in the ACh content of larvae, derived from the parents exposed to BPA (2–20 μg/L) either alone or in combinations with TiO_2_ (**Table 5**), while the AChE activity remained unaltered. The expression of mbp, syn2a, and α1 tubulin protein indicated that the mbp and syn2a (biomarkers for axon myelination and synapse formation) were reduced and α-tubulin (an intermediate filament protein, associated with the cytoskeletal organization of the developing neurons) remained unaltered in larvae (10 dpf) derived from the parents exposed to BPA alone, while reduced in larvae derived from the parents exposed to BPA (20 μg/l) in combination with TiO_2_ (100 μg/L) for 4 months.

#### 3.2.3 Cd

Cd is a highly toxic, non-essential heavy metal, and a common environmental pollutant found in the soil, contaminated water, and food (122, 123). Cd is used in various industries, including electroplating, battery production, and fertilizers. Cd is also a major component of tobacco. Acute exposure to Cd can cause inflammation and affect many organs, including the liver and kidney (123). There is a rising concern about the Cd effect on the endocrine system, since it has been demonstrated that Cd might mimic the activity of natural hormones such as estrogens and androgens, leading to the activation of specific signaling pathways or blocking the interaction of these hormones with their natural receptors (6, 124, 125).

Our searching strategy found only one article, which studied the effects of Cd on zebrafish either alone or in combination with TBT (32). Adult zebrafish (AB strain, 90 days old) were exposed to Cd (100 ng/L) and allowed breeding (**Table 2A**; **Supplementary Table 1**). In F1 generations (eggs, larvae, and adults) Cd treatment was continued as in parents (F0), and the larvae (F1) were assessed on 7 dpf, while adults (F1) were assessed after 5 months of exposure. The F2 larvae (obtained from F1 generations) were also exposed to 7 dpf in Cd. The brain, liver, and serum of the parents (both F0 and F1) were used for neurological analysis and thyroid disturbances, while the larvae (F1 and F2) were assessed for survival, neurotoxicity, and thyroid hormone deficiencies.

In parents (F0 adults) after 90 days of exposure, Cd was also unable to alter the plasma T3 and T4 contents in both male and female fish (**Table 4**). However, no significant effect on the dopamine and serotonin content and the AChE activity of the brain of male and female fish was observed (**Table 5**). The expression of the *crh* and *tshβ* genes remained unchanged in both male and female fish brains (F0) exposed to Cd for 90 days. In the liver of males and females (F0), the expression of *dio1*, *dio2*, *ttr*, *tg*, and *ugt1ab* remained unaltered after Cd exposure (**Table 6**).

In F1 fish (larvae), Cd exposure did not induce any significant effect in heart rates, survival or hatching, or malformations of the embryos (**Table 3**). Exposure to Cd also did not induce any significant change in the length of the larvae (F1, 7 dpf). Both T3 and T4 contents also remained unaltered in F1 larvae by Cd treatment when compared with controls (**Table 4**). Moreover, the neurotransmitter (dopamine and serotonin) contents and the AChE activity remained unaltered in F1 larvae (7 dpf) after Cd exposure (**Table 5**). Consequently, the expressions of HPT axis genes including *crh*, *tshβ*, *dio1*, *dio2*, *trr*, and *ugt1ab* remained unaltered, while expressions of *tg* reduced significantly in larvae (7 dpf) by Cd exposure (**Table 6**).

In F1 adults (5 months exposure to Cd), the THs (T3 and T4) remained unaltered in male fish, while in females, T3 remained unaltered, but T4 reduced significantly when compared with controls (**Table 4**). The neurotransmitters dopamine and serotonin and the AChE activity in the brain of male fish remained unaltered, while in females, the dopamine and serotonin content in the brain remained unaltered but the AChE activity reduced significantly when compared with controls (**Table 5**). In males (F1), the expression of HPT axis genes, including *crh*, *tshβ*, *dio1*, *dio2*, *ttr*, *ugt1ab*, and *tg* remained unaltered after Cd exposure. In F1 females, the expression of *crh*, *dio1*, *dio2*, *ttr*, and *ugt1ab* remained unaltered; however, the expression of *tshβ* and *tg* genes significantly reduced when compared with controls (**Table 6**).

In F2 larvae, embryo survivability, malformation in embryos, and larval body length during embryo-larval development was significantly decreased in fish exposed to Cd; however, heart rates and hatching did not significantly differ (**Table 3**). Compared with controls, both T3 and T4 contents in the whole body of the larvae significantly reduced in fish exposed to Cd alone (**Table 4**). Moreover, dopamine and serotonin contents and the AChE activity in the larval body (7 dpf) significantly decreased in fish exposed to Cd (**Table 5**). The expressions of *crh*, *tshβ*, *dio2*, *ttr*, *ugt1ab*, and *tg* in the larvae (7 dpf) were significantly downregulated when the fish were exposed to Cd; however, the expression of *dio1* in the whole body of F2 larvae (7 dpf) did not show any significant change by Cd exposure when compared with controls.

#### 3.2.4 Titanium dioxide

Titanium dioxide (TiO_2_) is extensively used in industrial and commercial products, including personal care products, surface coatings, paints, sunscreens, and food (126), and one of the commonly produced nanomaterials in the world (127). The sublethal effects of n-TiO2 exposure on aquatic life are reported to include oxidative stress and gill pathology reactions (128), retarded oogenesis and impaired reproduction. In zebrafish, TiO2 induced brain injuries, alterations in neurochemicals, and spatial recognition of memory impairments (129).

Our search strategy found only one article (28) where TiO2 was used as a modulator of the effects induced by BPA. Zebrafish adults (AB strain, 90 days old) were exposed to TiO2 (100 μg/L) for 4 months and allowed breeding in a treatment-free (no TiO2) environment to obtain F1 eggs and larvae. Moreover, the peripheral blood, liver, gonad, and brain of the parental fish (F0) were also analyzed. The development and growth of the F1 eggs and larvae were observed in control (no TiO2) conditions. The developmental abnormalities (survival, hatching, locomotion, and growth) in F1 eggs and the locomotor activity, thyroid hormone concentrations, neurotransmitter contents, and AChE activity were assessed in larvae (10 dpf).

Accumulation of titanium was observed in both male and female fish (F0). A significant amount of titanium was accumulated in the F1 eggs of the fish exposed to the metal parenterally (F0 males and females) (**Table 3**). No effect was observed in hatching, malformation, and survival of the larvae (F1) derived from the parents exposed to TiO_2_ for 4 months (130). Both T3 and T4 levels remained unaltered in the plasma of F0 parents, and in the eggs and larvae of F1 generations (**Table 4**). In females (F0), compared with controls, the serotonin and dopamine contents in the brain significantly reduced, while the ACh content remained unaltered. In males (F0), titanium exposure did not induce any significant change in the serotonin, dopamine, or acetylcholine contents in the brain (**Table 5**). Significant decrease in the swimming speed in light phase was observed in the larvae derived from the parents exposed to TiO_2_ for 4 months (**Table 5**). During the light-dark transition, the average swimming speed in F1 larvae (derived from the parents exposed to TiO2 for 4 months) was significantly lower than the control larvae. Consequently, there was a significant decrease in the serotonin and dopamine contents, while ACh content remained unaltered in larvae (5–10 dph) derived from the parents exposed to TiO2 for 4 months. Moreover, AChE activity in these larvae remained unaltered (**Table 5**). The expressions of mbp, syn2a, and α1 tubulin protein indicated that the mbp, syn2a, and α-tubulin was reduced in larvae derived from the parents exposed to TiO2 for 4 months (**Table 7**; **Supplementary Table 1**).

#### 3.2.5 Pb

Pb is a toxic metal widely used for many years in the industry (101, 131, 132), and due to mismanagement, it is now spread in the environment. Although significant efforts have been made to diminish Pb levels in the environment, such as the ban of Pb gasoline or Pb paint, as a nondegradable toxicant, Pb is still substantially present in the environment and exposure to this metal is still happening (133–135). In human and animal studies, it was observed that Pb affects almost all systems, including cardiovascular (135–138), endocrine (139–142), nervous (138, 143), immune, and many others (144).

Our literature search strategies found only one article in which Pb is used as a modulator of the effects induced by BDE-209 (**Tables 3**–**7**; **Supplementary Table 1**), and the data were compared with the data obtained from the fish exposed to Pb alone (27). Zebrafish adults were exposed to Pb (10 μg/L) for 4 months, and the parents were assessed for length, weight, organ weights (liver, brain, gonad), thyroid (T3 and T4), and sex steroid hormone (T and E2) levels in serum, and the fecundity of the fish during the last 7 days of exposure. The eggs (F1) were assessed for the accumulation of Pb in eggs due to parental exposure, developmental disorders, hatching, TH (T3 and T4), and sex steroid hormone concentrations. The F1 larvae (5 dpf) were assessed for growth (length and weight), accumulation of Pb in the larval body due to parental exposure, TH (T3 and T4) contents, sex steroid hormone (T and E2) contents, and the expressions of thyroid hormone receptor associated protein 3b, apolipoprotein Bb and Ea, cathepsin 8, various crystalline proteins, muscular proteins, neuronal proteins, and neurotransmitters (**Tables 3**–**7**; **Supplementary Table 1**).

It was observed that in parents, Pb (10 μg/L) did not show any significant change in the length and weight of the male and female fish; however, the condition factor in males, compared with controls, decreased significantly. The HSI, BSI, and GSI remained unaltered (**Table 3**; **Supplementary Table 1**). Also, Pb exposure (10 μg/L) was unable to alter the serum T3 levels in adult males and females; however, serum T4 remained unaltered in males and significantly reduced in females (**Table 4**: **Supplementary Table 1**). The serum T and E2 levels in male fish (F0) did not show any significant alteration; however, in females, Pb (10 μg/L) exposure (4 months) reduced both T and E2 levels significantly, even though the fecundity remained unaltered (**Table 3**).

In F1 eggs, parental exposure showed significant accumulation of Pb in the eggs and induced significant hatching delay, although the size of the eggs remained unaltered, and no significant developmental abnormalities were observed (**Table 3**). The T3 and T4 contents in F1 eggs did not show any significant difference by Pb exposure (parental) when compared with corresponding control eggs; the T content was found to be significantly reduced while E2 remained unaltered (**Table 4**).

In F1 larvae (5 dpf), significant accumulation of Pb was observed, and the condition factor significantly reduced even though the length and weight of the larvae remained unaltered (**Table 3**). The T content significantly reduced while the T3, T4, and E2 contents remained unaltered (**Table 4**). Downregulation of the thyroid hormone receptor protein 3b was observed in larvae derived from parents exposed to Pb (10 μg/L) (**Table 7**). The swimming behavior of the larvae (average swimming speed) significantly reduced both in light and dark phages (**Table 5**). The expression of thyroid hormone-associated protein 3b was downregulated, while the steroid-responsive vitellogenin showed universal overexpression (**Table 7**). Among the 12 neurotransmitters studied, GABA, tryptophan, and ACh were upregulated and glutamate was downregulated in larvae derived from the parents exposed to Pb (10 μg/L) alone (27). The neuronal proteins, syn2a, and mbp significantly downregulated by Pb, while several crystalline proteins and muscular proteins were affected by Pb in F1 larvae (**Table 7**; **Supplementary Table 1**). Moreover, the downregulation of cathepsin S was also observed in larvae derived from parents exposed to Pb (10 μg/L) for 4 months (**Table 7**).

#### 3.2.6 TDCPP

Tris (1, 3-dichloro-2-propyl) phosphate (TDCPP) is one of the most popular organophosphate esters (OPEs), which is widely used as a flame retardant additive for furniture, children’s foam toys, and automotive interiors, and has a high detection rate in the environment (145). In the indoor air in the USA, the concentration of TDCPP is as high as 56.08 mg/g (146), and in China is 40.1 mg/g (147). The average measurable amounts of TDCPP in Chinese surface water and soil were 85 ng/L (148) and 65.7 ng/g (149), respectively. This condition puts the human body at high risk of exposure to TDCPP, and it has become one of the most commonly detected OPEs in humans. For example, its concentration can reach 0.51 ng/L in plasma (150), 0.4 μM in breast milk (151), 82.8 ng/g in placenta (152), and 11 ng/mL in urine (153). Our literature search found only one article where TDCPP induced neuronal disorders in zebrafish (31).

Zebrafish adults (4 months old) were exposed to TDCPP (4, 20, and 100 μg/L) for 3 months (F0),and the laid eggs (F1) and the hatched larvae (F1) were maintained in treatment-free conditions (10 dph) until analysis. The parents (F0) were used for the analysis of the accumulation of TDCPP, and its metabolite BDCPP in the whole body (body burden) of the fish. Moreover, the thyroid hormone levels (T3 and T4) in the plasma of the fish (31) were also measured. The F1 eggs were used for TDCPP accumulation, hatching of the embryos, and thyroid hormone contents. The F1 larvae (5 and 10 dpf) were used for analyses of developmental disorders, behavior, oxidative stress, neurotransmitter contents, AChE activity, gene expression, protein expression, and thyroid hormone contents.

It was observed that the accumulation of TDCPP in the body (body burden) of adult male and female fish was concentration dependent and the females apparently accumulated more TDCPP from the environment than males (31). Significant amount of BDCPP (metabolite of TDCPP) was also found in the body of both male and female fish (**Table 3**). It was also observed that in females, TDCPP significantly reduced plasma T4 (only in 20–100 μg/L, no effect in 4 μg/L) and T3 (only in 100 μg/L) levels in a concentration-dependent manner (**Table 4**). However, in males, TDCPP remained ineffective in altering any significant change in TH levels (both T3 and T4) in the plasma of the fish. (31).

In F1 eggs, a significant amount of accumulation of TDCPP was observed, which is dependent on the exposure concentration of the F0 parents (the higher the concentration of exposure of adult fish, the higher the accumulation of TDCPP in eggs). Moreover, the concentration of metabolite BDCPP also appeared to be concentration dependent, even though the concentration was negligible compared to TDCPP. It was also observed that there was a significant concentration-dependent decrease in hatching rates of the 3 dpf embryos derived from parents exposed to TDCPP (20–100 μg/L) for 3 months. T4 levels were significantly reduced in eggs derived from the fish exposed to 100 μg/L TDCPP, while T3 content remained unaltered in the eggs obtained from the parents exposed to TDCPP (4, 20, and 100 μg/L) for 3 months (**Table 4**).

The growth of the larvae (F1) was significantly inhibited as observed in 5 and 10 dpf in a concentration-dependent manner (significant only in larvae derived from the parents exposed to 100 μg/L TDCPP). The malformation (spinal curvature) of the larvae increased, and survivability (5 and 10 dpf) decreased in a concentration-dependent (parents exposed to 100 μg/L for 5 dpf, and 20 and 100 μg/L for 10 dpf) manner (**Table 3**). T4 content was significantly reduced in 5dpf larvae derived from the fish exposed to 100 μg/L TDCPP. However, in 10 dpf larvae, T4 content significantly reduced when the parents were exposed to both 20 and 100 μg/L TDCPP. In contrast, T3 content remained unaltered in the larvae (5 and 10 dpf) obtained from the parents exposed to (4, 20, and 100 μg/L) for 3 months (**Table 4**).

The swimming speed significantly reduced in the larvae (5 dpf) during the light phase and light-dark transition phase, which were obtained from the parents exposed to 100 μg/L TDCPP for 3 months. The average swimming speed in 10 dpf larvae derived from fish exposed to 100 μg/L TDCPP for 3 months, was significantly reduced than the larvae obtained from control fish. The neurotransmitter analysis (serotonin, dopamine, histamine, and GABA) was made in the larvae (5 and 10 dpf) obtained from parents exposed to TDCPP for three months (**Table 5**). In 5 dpf larvae histamine content did not show any significant change; however, serotonin content was significantly reduced when the parents were exposed to TDCPP (100 μg/L) for 3 months. The dopamine and GABA contents were also reduced in 5 dpf larvae when the parents were exposed to TDCPP (20 and 100 μg/L). In 10 dpf larvae, serotonin and GABA contents were significantly reduced when the parents were exposed to the highest concentration of TDCPP (100 μg/L) used in this study; while dopamine and histamine significantly decreased in larvae (10 dpf) when the parents were exposed to both 20 and 100 μg/L TDCPP for 3 months. In contrast, the AChE activity remained unaltered in both 5 and 10 dpf larvae when the parents were exposed to TDCPP (4, 20 and 100 μg/L) for 3 months (31) (**Table 5**).

The generation of ROS is also evaluated in F1 larvae derived from the parents exposed to 100 μg/L TDCPP for 3 months. It was observed that ROS significantly increased in these larvae (10 dpf) (**Table 3**). Moreover, both 5 and 10 dpf larvae were used for gene analysis (**Table 6**). The mRNA expression pattern of *mbp, gap-43, syn2a, gfap*, and *α1-tubulin* were analyzed in the larvae derived from the fish exposed to TDCPP for three months. It was observed that in 5 dpf larvae derived from the fish exposed to 20 and 100 μg or TDCPP, the expression of *mbp* was downregulated in larvae compared with controls. There was also a significant downregulation of *α1-tubulin* in larvae (5 dpf) derived from the parents exposed to 100 μg/L TDCPP. Other mRNAs such as *syn2a, gfap*, and *gap-43* remained unaltered in larvae when parents were exposed to TDCPP (4, 20, and 100 μg/L) for 3 months. In 10 dpf larvae, derived from parents exposed to 100 μg/L TDCPP, the expression of *mbp* and *syn2a* were significantly downregulated than the larvae obtained from control fish. Moreover, the expression of *α1-tubulin* was also downregulated when the larvae were derived from the fish exposed to 20 and 100 μg/L. In contrast to these mRNAs, the expression of *gap-43* was significantly upregulated in 10 dpf larvae derived from the parents exposed to 100 μg/L TDCPP, while the expression of *gfap* mRNA remained unaltered in 10 dpf larvae derived from all the experimental fish exposed to TDCPP (4, 20, and 100 μg/L) for 3 months (**Table 6**).

Protein expression analysis indicated that parental exposure to TDCPP (100 μg/L) significantly reduced mbp content in 5 dpf larvae. Moreover, significant reduction in α1-tubulin and syn2a was also observed in larvae derived from the parents exposed to 20 and 100 μg/L TDCPP. All three proteins (mbp, syn2a, and α1-tubulin) were significantly reduced in 10 dpf larvae when the parents were exposed to 20 and 100 μg/L TDCPP (**Table 7**).

#### 3.2.7 TBT

TBT is an organotin which has been restricted by many countries as antifouling agent, due to its ecotoxicological properties. Because of the difficult degradation characteristics in the sediments and the nontarget toxicity, the adverse influence of TBT is still a great concern (154, 155). Moreover, TBT has been detected in aquatic environments (156, 157). The literature involving effects of organotin in biota has focused primarily on reproductive toxicity, such as the ability of TBT to sex skewing (females develops phenotypic male sex organs) in many aquatic organisms (158–160). Also, TBT-induced neurotoxicity, developmental toxicity, and endocrine disruption have been reported (161–164).

Our literature search found only one article, which studied the toxic potentials of TBT either alone or in combination with Cd (32). Zebrafish adults (AB strain 90 days old) were exposed to TBT (100 ng/L) either alone or in combination with Cd (100 ng/L) for 90 days and assessed for endocrine and neurological disorders (**Tables 2A**, **ST-1**). The F1 generations (eggs, larvae, and adults) were continued to be exposed in the TBT and Cd either alone or in combinations (as parents were). The larvae (F1) were assessed on 7dpf while adults (F1) were assessed after 5 months of exposure. The F2 larvae (obtained from F1 generations) were also exposed until 7 dpf in TBT and Cd, either alone or in combinations. The brain, liver, and serum of the parents (both F0 and F1) were used for analysis of neurotoxicity and thyroid disturbances, while the larvae (F1 and F2) were assessed for survival, neurotoxicity, and thyroid hormone content.

In parents (F0 adults), after 90 days of exposure to TBT, either alone or co-exposed with Cd did not alter the TH levels (both T3 and T4) in males, while in females, TBT alone was unable to alter the TH levels; however, significantly reduced TH levels (both T3 and T4) when coexposed with Cd (**Table 4**). The dopamine content and the AChE activity in the brain of male and female fish remained unaltered in F0 parents after 3 months of exposure to TBT and Cd, either alone or in combinations (**Table 5**). However, the serotonin content in the brain of male fish remained unaltered (in all treatment groups), while in females, a significant decrease in the serotonin level was observed in the brain when the fish were exposed in combinations with Cd (TBT+Cd), even though TBT treatment alone did not show any significant alteration (**Table 5**).

The expression of the *crh* gene remained unchanged in male fish brains exposed to TBT, alone or in combination (Cd+TBT). However, the expression of *tshβ* was upregulated only in fish exposed in combinations, not in those exposed to TBT alone. In female brain, both *crh* and *tshβ* was upregulated when the fish were coexposed in combinations (Cd+TBT), while remaining unaltered by TBT alone (**Table 6**). In liver of males (F0), the expression of *dio1*, *dio2*, *ttr* and *tg* remained unaltered in fish exposed to TBT either alone or in combinations; however, the expression of *ugt1ab* was significantly upregulated only in the co-exposed group (Cd+TBT). In liver of females (F0), the expression of *dio1* and *ttr* did not show any significant changes when exposed to Cd or TBT either alone or in combinations; however, the expression of *dio2* and *ugt1ab* was upregulated and *tg* was downregulated only in fish exposed to Cd and TBT in combination (Cd+TBT).

In F1 fish (larvae), no effect was observed in survival rates and hatching of the embryos exposed to TBT either alone or in combinations (**Table 3**). Significant decrease in heart rates was observed when the fish were exposed to a combination of TBT+ Cd; single exposure to TBT did not induce any significant change in heart rates. A decrease in the larval length (7 dpf) and increase in malformation rate were observed in fish exposed to TBT either alone or in combination with Cd (**Table 3**). Both T3 and T4 contents of the whole body of the larvae (7 dpf) remained unaltered when the fish were exposed to TBT alone; moreover, coexposure with TBT and Cd significantly reduced both T3 and T4 contents of the larvae when compared with the controls (**Table 4**). The neurotransmitters dopamine and serotonin contents and the AChE activity in the F1 larvae (7 dpf), decreased significantly in fish exposed to TBT with Cd, while TBT alone did not induce any significant change in the dopamine and serotonin contents as well as AChE activity of the F1 larvae (**Table 5**). The expression of *crh,dio2*, and *trr* genes in F1 larvae remained unaltered by TBT and Cd exposure (either alone or binary), however, *tshβ* and *tg* were down regulated in binary exposure and remained unaltered in single exposure experiments. The gene *dio1* was downregulated by TBT either alone or in combination, however, it remained unaltered after Cd (alone) exposure. The expression *ugt1ab* was downregulated by TBT either alone or in combination with Cd; however, it remained unaltered by Cd alone.

In F1 adult fish (5 months exposure), plasma T3 level in male and female fish remained unaltered by TBT and Cd when exposed alone, while coexposure, significantly reduced T3 levels, in both male and female fish. Plasma T4 level in male fish remained unaltered if exposed to TBT alone, while coexposure (Cd+TBT) significantly reduced T4 levels in the plasma of male fish. In females, plasma T4 level was significantly reduced in fish exposed to TBT either alone or in combination (**Table 4**). In the male brain, the neurotransmitters dopamine and serotonin remained unaltered while AChE activity decreased significantly by TBT alone (**Table 5**). Coexposure with Cd resulted significant reduction in the dopamine and serotonin contents as well as AChE activities in the brain of male fish (F1). In adult F1 females, TBT either alone or in combinations with Cd was able to significantly reduce the dopamine and serotonin contents in the brain, while the AChE activity remained unaltered by TBT alone. Significant reduction in the AChE activity was observed when the fish were (F1 females) coexposed to TBT and Cd (**Table 5**). In males (F1), the expression of *crh* and *tshβ* in the brain and the expression of *dio1, dio2, ttr, tg, ugt1ab*, did not show any significant alteration in fish exposed to TBT alone or in combinations with Cd (**Table 6**). In females (F1), the expression of *crh* in the brain showed significant downregulation when co-exposed to Cd and TBT. However, TBT alone was unable to induce any significant change in *crh* expression. The expression of *tshβ* in the brain of female fish was downregulated significantly when the fish were exposed to TBT either alone or in combination with Cd. In the liver, the expressions of *dio1*, *dio2*, and *ttr* did not show any significant alteration when exposed to TBT either alone or in combination with Cd. However, the expression of *tg* was significantly downregulated by TBT either alone or in combination and the expression of *ugt1ab* was downregulated only in fish (F1 females) exposed to a combined mixture of Cd and TBT. Moreover, TBT alone has no significant effect on the expression of *ugt1ab* in the liver of female fish (**Table 6**).

In F2 larvae, survivability during embryo-larval development was significantly decreased in fish exposed to TBT, either alone or in combination with Cd (**Table 3**). Hatching was significantly delayed in the embryos exposed to TBT with Cd; however, not significantly differ in embryos exposed to TBT alone. Significant decrease in heart rates was observed when the fish were exposed either to TBT alone or together with Cd. Significant induction in the malformation rates, and reduction in the length of the F2 larvae (7 dpf) were observed when exposed to TBT, either alone or in combinations (**Table 3**). Compared with controls, both T3 and T4 contents in the whole body of the F2 larvae significantly reduced in fish exposed to TBT either alone or in combinations (**Table 4**). Dopamine content in the larval body (7 dpf) significantly decreased in fish exposed to TBT either alone or with Cd. The serotonin content in the whole body of the larvae (7 dpf) showed significant reduction when the fish were exposed to TBT either alone or in combination. The AChE activity in the whole body of the larvae (7 dpf) significantly decreased in fish exposed to TBT either alone or in combinations (Cd+TBT) (**Table 5**). The expressions of *crh, tshβ*, *dio1*, *dio2, ttr, ugt1ab* and *tg* in the larvae (7 dpf) were significantly downregulated when the fish (F2 larvae) were exposed to TBT either alone or in combinations with Cd.

## 4 Discussion

EDCs impact on aquatic life and human health via acute or chronic exposures occurs throughout the life. EDCs interact with the normal activities of the body by activating or blocking hormone receptors, disrupting the synthesis, or degradation of the hormones. Anthropogenetic sources of EDCs are mainly from solid and liquid wastes, as well as leaching from manufactured products disposed in the environment. Contaminated surface water is a major EDC exposure route to aquatic life and may also contribute to human exposure via food chain pathways (76). EDC levels in surface water ranged from nano- to micromolar, with higher concentrations where the surface water received the treated effluent from the wastewater treatment plants and hospital sewages (76). The disruption of endocrine systems by EDCs could have the potential to induce neuronal disorders and regulate the expression of several neuronal genes (165). Zebrafish have become a popular model for the study of vertebrate development and gene functions (166, 167). The virtually transparent embryo of this species and the ability to accelerate genetic studies by gene knockdown or overexpression have led to the widespread use of zebrafish in the investigation of vertebrate gene function and the study of human genetic diseases (17).

The objective of this systematic review is to determine how EDCs affect the development and function of the nervous system in zebrafish. To address this aim, we conducted a comprehensive search of peer-reviewed literature indexed in the PubMed database, following PRISMA 2020 guidelines (26). Although the search was limited to a single highly reliable and reputable database (168), we identified 14 chemicals (**Table 1**) across 12 research articles (see Flow diagram, **Tables 2A**, **B**) classified as EDCs in zebrafish. From these studies, we extracted multiple toxicological (**Table 3**), endocrinological (**Table 4**), and neurobehavioral endpoints (**Table 5**) as well as potential genes (**Table 6**) and proteins (**Table 7**) that serve as indicators of neurodegenerative disorders.

Both embryos (AB and Tu strains) and adults (AB strain) of zebrafish were exposed to these chemicals (EDCs) in laboratory conditions, and the studies were made during embryo-larval development, eggs, larvae, and adults in F0 generation as well as transgenerational effects were observed in F1 and F2 generations (**Tables 2A**, **2B**). It was expected and also observed that during exposure periods, the embryos and the adults accumulated significant amount of the chemicals from the environment either as parent compounds (Pb, Ti) or metabolites (PBDE, BDCPP), and sometimes the accumulation is sex-specific (TDCPP) and have potential for transgenerational inheritance in F1 and F2 generations even though the F1 and F2 generators (except the studies made by 32) were mostly maintained in the treatment-free environment (27, 28, 31, 35, 38). Moreover, the metals (Pb and Ti) significantly enhanced the accumulation of the test compounds in binary exposures (27, 28).

The significant toxicological endpoints focused on were coiling frequency in embryos during development, hatching delay, fecundity, heart rates, pericardial and yolk sac edema, growth (length and weight), curved spine and skeletal deformities, swim bladder inflation, survivability rates, and disruption of lateral stripe melanocytes formation/deposition (**Table 3**). The endocrinological endpoints are thyroid hormone levels/contents (T3, T4), sex hormones (E2 and T), thyroid, and gonad histology, HSI, GSI and expression of the related hepatic and gonadal genes (**Tables 4**, **6**). The neurobehavioral endpoints are swimming behavior and movement, BSI, neurotransmitter contents, AChE activity, and related genes and proteins expressed in the nervous system (**Tables 5**–**7**). Despite wide variability in experimental design, maintenance, age, and strain of the zebrafish (**Table 2B**), the targeted endocrine system was found to be thyroid (ATZ, BDE-209, mBDE-47, BPA, BPS, Cd, OBS, Pb, TBT, TCEP, TDCPP, THM, TiO_2_) and steroid (BDE-209, E1, Pb) hormones synthesis and pathways, and the neurotransmitters (ACh, dopamine, serotonin, norepinephrine, etc.) and neural peptides (smp2a, mbp, etc.) and the enzyme (AChE) in the nervous system (**Tables 3**–**7**; **Supplementary Table 1**).

Further, these 14 EDCs were also grouped into six categories and, seven of them (ATZ, BPS, E1, mBDE-47, OBS, TCEP, THM) were used on embryos when the neuroendocrine system is ill-developed, and six of them were in adults (BDE-209, Cd, Pb, TBT, TDCPP, TiO2) when the neuroendocrine system is fully functional. Moreover, only BPA was studied on both embryos and adults. Further, the binary effect was investigated only on adults with metals (BPA and Pb, TBT and Cd, and BDE-209 and Pb) (**Tables 2A**, **2B**; **Supplementary Table 1**). Further, the transgenerational effects were studied in F1 and F2 generations derived from the adults exposed to BDE-209 and Pb (either alone or in combinations), BPA and Ti (either alone or in combination), TBT and Cd (either alone or in combinations), and TDCPP. Moreover, embryos exposed to OBS (2 hpf–21 dpf and depurated until 180 dpf) and BPS (2hpf–120 dpf adults) were also used for transgenerational studies by generating F1 larvae.

The eight EDCs evaluated in zebrafish embryos exhibited substantial variability in exposure conditions and tested concentrations. Our analysis indicates that among these compounds, the concentrations required to induce neurological disorders during embryonic development were lowest for E1 and highest for BPA, following the gradient: E1<mBDE-47<ATZ<BPS<OBS<TCEP<THM<BPA. In other words, within this group of EDCs, the steroid hormone [E1] demonstrated the greatest potency as an inducer of neurological impairment, whereas the plastic component BPA was the least potent. These findings align with the classification hierarchy: steroid hormone [E1] < brominated compounds [mBDE] < boicides [ATZ] < plastic components [BPS] < flame retardants [OBS, TCEP] < biocides [THM] < plastic components[BPA]) Thus, during embryo-larval development, when the neuroendocrine system has not yet matured, environmental exposure to E1 appears more effective at inducing neurological disorders than exposure to BPA. This heightened sensitivity is likely due to the ability of E1 to target genes associated with neuroendocrine pathways in zebrafish (36).

The mode of exposure of EDCs used for zebrafish adults is not consistent with the embryos (**Table 2A**). Adults with full functional nervous systems and endocrine organs have been exposed for a prolonged period [3–4 months] either alone or in combination with metals (Cd+ TBT, Pb+BDE-209; Ti +BPA) (**Tables 2A**), which is different from the conditions used in embryonic exposures. Moreover, the transgenerational studies were also made in a few cases in F1 eggs and larvae, as well as in F2 larvae (TBT and Cd). Additionally, due to prolonged exposure, the accumulation of chemicals, which is sex-specific in many cases (Cd, Pb, Ti), as parental compound or metabolite was also observed in body burdens of adults (BPA; Ti; BDCPP as metabolite of TDCPP), F1 eggs (BDE-209 as PBDE; Pb; BPA and Ti, mostly TDCPP very little BDCPP) and larvae (Pb) of zebrafish. The neuroendocrinological disorders observed in F0 adults are disruptions in TH (T3 and T4), T and E2 levels, neurotransmitters, AChE activity, alteration in syn2a, mbp, α1-tubulin mRNAs and proteins (**Tables 4**–**7**) and up- and downregulation of many genes related to thyroid and reproductive pathways (**Table 6**).

Among the three studied metals (Cd, Pb, and Ti), adult zebrafish were exposed for 3 months with Cd and Pb and 4 months with Ti (**Table 2A**), with the concentration of Ti was found to be higher than other two (Cd<Pb<Ti). Although the serum T3 and T4 levels remained unaltered after Ti exposure, the sex-specific alteration in the neurotransmitter contents and AChE activity was observed (serotonin and dopamine reduced, and ACh remained unaltered in females; no alteration in males). Fish exposed to Pb (3 months), showed sex-specific alteration in serum thyroid and sex steroid hormone levels (no alteration in serum T3, T4, T, and E2 levels in males; in females, except T3, which remained unaltered, T4, T, and E2 levels significantly reduced). In Cd-exposed fish, no effect was observed in serum T3 and T4 levels, as well as serotonin and dopamine contents, and AChE activity in male and female fish. Therefore, despite the variability in concentration and duration of exposure, the Ti alone was found to have modulating effects in the studied neuroendocrine parameters, while Cd remained unresponsive in these respects.

Cd and Pb are well-established endocrine disruptors in zebrafish, impacting both reproductive and thyroid systems, while Ti has a less-studied endocrine-disrupting profile, primarily impacting the thyroid system. Cd and Pb disrupt the thyroid axis by downregulating key genes and affecting hormone levels, and Cd can also interfere with the HPT axis and cause neurobehavioral effects (27, 32) Ti has been shown to affect thyroid hormone levels and has been linked to other endocrine disruptions in zebrafish larvae (28), but more research is needed to fully understand its effects. Brominated compounds, biocides, flame retardants, plastic components, and E1 can have neurotoxic effects, impacting brain development, cognitive function, and motor skills. These compounds can disrupt neurological functions through mechanisms like oxidative stress, mitochondrial dysfunctions, and interference with hormone regulation and neurotransmitter pathways. Exposure during critical developmental periods, particularly during embryo-larval development, is linked to adverse outcomes like learning disabilities, behavioral issues, and potentially Alzheimer’s and Parkinson’s disease-like symptoms in zebrafish (165, 169).

Moreover, when classifying the EDC by category, we observed that E1 (steroid hormone) was used exclusively during embryo-larval developmental stages of zebrafish (0–5 dpf and 4–5 dpf). E1 exposure resulted hypoactivity in locomotor behavior and induced signification alterations in neuronal gene expression, thereby impacting nervous system development. Among the affected genes, *apoea, fbl, sigmar1* were upregulated while *ddc*, *enox1* and *ttr* was down regulated (**Table 6**). The brominated compound mBDE47 (a metabolite of PBDE) disrupted coiling frequency during embryonic development, downregulated *tph2* (tryptophan hydroxylase) mRNA and induced apoptosis 5-HT (serotonin) expressing neurons likely through interference with thyroid hormone regulation (33). Another brominated compound BDE-209, a potent modulator of locomotor behavior (117), was administered to adults (F0) alone or in combination with Pb and neurotoxicity was evaluated in F1 larvae (5 dpf). Significant reductions in the neural proteins syn2a and mbp were observed, along with alterations in several other neuronal proteins and neurotransmitters (27). The interaction between BDE-209 and Pb resulted in transgenerational developmental neurotoxicity in the offspring, inhibiting neuronal growth and neurotransmitter signaling and inducing neurobehavioral deficits.

The biocides ATZ (an organochlorine) and THM (an organofluoride) when administered during embryo-larval development, induced significant behavioral changes including concentration-dependent inhibition of movement-related behaviors and disrupted the expression of genes associated with nervous system development. ATZ upregulated *sult2bl* and downregulated *cdk5* and *cyp26b*, whereas THM upregulated *flk1*, *neurog1* and *vegf1* and downregulated *ldha* expression in zebrafish (29, 37) Additionally, THM increased serotonin (5-HT) and norepinephrine (NE) levels, and reduced AChE activity (37). The biocide TBT (an organotin) administered to adults with or without Cd for 3 months and continued across F1 and F2 stages (32). As a biocide, TBT showed minimal neurotoxic effects with regard to genes related to HPT axis (*crh* and *tshβ*); however, significant disruption was observed in F2 generations showed minimal neurotoxic effects on genes related to the HPT axis (crh, tshβ) in adults; however, significant disruptions emerged in F2 larvae (32). Overall, the biocides administered during embryo-larval development or adulthood have the potential to disrupt neurodevelopment, neuronal function, and behavior by altering key CNS genes (**Table 6**) with greater complexity observed under in binary exposures.

Flame retardants (TCEP and OBS) administered during embryo-larval development also disrupted the expression of several genes involved in nervous system development. TCEP upregulated *gap43, syn2a*, and *mbp* and downregulated *α1-tubulin* and *elavl3* in a concentration-dependent manner. Parental exposure to OBS significantly upregulated *syn2a* and *mbp* and reduced swirl-escape responses (a locomotor behavior) in F1 larvae (7 dpf). The upregulation of *syn2a* and *mbp* might affect synapse formation and neurotransmission, thereby influencing neurobehavior (38). Adult exposure to TDCPP, an organophosphate flame retardant (31), significantly decreased locomotion in F1 larvae and downregulated genes (*α1-tubulin, mbp, syn2a*) associated with neuronal development, along with reduction in multiple neurotransmitters (serotonin, dopamine, GABA, histamine) (**Table 6**). Thus, flame retardants, whether organophosphate (TCEP and TDCPP) or perfluoroalkyl substance (OBS) disrupt nervous system development either directly or via transgenerational transfer.

Zebrafish embryos were also exposed to plastic components (BPS and BPA), and the adults were exposed to BPA with or without TiO2 (28, 35, 36). Parental BPS exposure upregulated *gfap*, *gap43* and *mbp* and downregulated syn2a in F1 larvae (96 hpf) accompanied by reduced motility (35). BPA exposure during embryonic (0–5 dpf) or early larval (96–120 hpf) stages altered locomotor activity and modulated estrogenic and non-estrogenic genes associated with neurological disorders (36). In adults, (serotonin, dopamine and ACh were unaffected, however, larvae exhibited significant concentration-dependent reductions in serotonin and dopamine, while ACh and the enzyme AChE remained unchanged. Consequently, the expression of mbp and syn2a were downregulated in a concentration-dependent reduction, whereas α1-tubulin expression remained unaltered, (**Table 7**).

Adult zebrafish were also exposed to metals (Cd, Pb, and Ti) either alone or in combinations with other EDCs (Cd + TBT; Pb + BDE-209; TiO2 + BPA) as well as TDCPP. Evaluations were performed across F0 adults (BDE-209, BPA, TDCPP, and TBT), F1 eggs and larvae (BDE-209, BPA, and TBT), F1 adults (TBT), and F2 larvae (TBT). In general, although these metals significantly affected endocrine function, their combined exposures modulated the effects of TBT, BPA, and BDE-209.

The brain development in zebrafish is a rapid process that begins with early regional demarcation by 12 hpf and progresses to the establishment of functional systems by 3 dpf. Key milestones include the formation of the basic neural tube structure by 10 hpf, initial regionalization by 12 hpf, major organ system maturation by 36 hpf, and the hatching period between 48 and 72 hpf, after which the nervous system is functional (170, 171). TH regulates zebrafish brain development by controlling the timing and differentiation of neural cells, including neurons and oligodendrocytes, influencing myelination, and interacting with other signaling pathways like retinoic acid (RA). TH also promotes neural crest cell (NCC) migration and patterning necessary for craniofacial development and regulates the development of blood vessels within the brain. These effects are mediated by TH receptor binding to DNA and altering the expression of specific genes involved in neural cell development and function (172). Maternal THs, stored in the zebrafish egg, are essential for proper brain development, acting before the embryo’s own thyroid gland is functional (173). During the initial three days (72 hpf) of development, the zebrafish embryo’s thyroid gland is not yet formed, making it entirely dependent on the maternal THs stored in the yolk sac. These maternal THs regulate the differentiation of specific neural cell populations, ensure the correct patterning of the brain and spinal cord, and affect the expression of key developmental genes like *pax2a* and *pax7* (173). Knocking down the specific TH transporter (MCT8), which blocks the uptake of maternal THs, leads to profound and irreversible neurological deficits, including impaired motor function and altered neuron populations, highlighting the critical time-dependent and irreversible role of maternal THs (174). Therefore, disruption of the thyroid hormone storage coming from maternal sources during embryonic-larval development or during transgenerational inheritance by EDCs is the potential cause of neurodegenerative disorders observed in zebrafish. Moreover, sex steroid disruption by EDCs (BDE-209, E1, and Pb) in zebrafish embryos and adults causes a wide range of reproductive and developmental disorders, including skewed sex ratios (masculinization or feminization), abnormal gonad development, altered sexual behavior, and reduced fertility. These effects are probably mediated through the HPG axis genes, enzymes and neurotransmitters that can be persistent or irreversible, particularly after exposure during embryo-larval development (175–178).

Taken together, our literature-based analysis indicates that EDCs including metals, brominated compounds, flame retardants, biocides, plastic components, and sex steroid hormones, administered during embryo-larval development or adulthood, significantly disrupt neurodevelopment, neuronal function, and behavior by altering key genes involved in central nervous system development and function (**Table 6**). These effects are compounded under binary exposures. Moreover, the transgenerational transfer of the EDCs further disrupts nervous system development, posing potential threat to human health. Although several EDC-induced neuronal disorders have been highlighted in zebrafish, important knowledge gaps remain. For example, ATZ has been shown to induce epigenetic modifications during embryogenesis in zebrafish; however, the epigenetic effects of other EDCs remain largely unexplored. Additionally, neurobehavioral assessments have primarily focused on larval locomotion, other behavioral domains such as feeding, reproduction, pain response, and stress, in both larvae and adults warrant further investigations. Moreover, binary exposure studies have thus far been limited to metals in adult fish; the use of binary or multi-compound mixtures in embryo-larval models could further advance our understanding of nervous system development. Further, the effects of EDCs on the regenerative capacity of the zebrafish nervous system also merit investigations.

## 5 Conclusion

Our systematic review of literature identified 14 EDCs, including metals, brominated compounds, flame retardants, biocides, plastic components, and the steroid hormone E1, that exhibit the potential to induce neurodegenerative disorders in zebrafish during embryo-larval development as well as in F1 and F2 generations through intergenerational and transgenerational inheritance. Zebrafish possess substantially more active adult neurogenesis and a markedly greater regenerative capacity than mammals, particularly following injury. In contrast, mammals exhibit limited neurogenesis, and injury typically result in minimal or no regeneration. Notably, with the exception of bisphenol A (BPA), the EDCs identified in embryo and adult exposure studies were not identical, and adult zebrafish were exposed for longer durations than embryos.

Most of the identified EDCs mostly target the thyroid endocrine system. Because zebrafish embryos lack a functional thyroid gland during early development, they rely on maternally derived thyroid hormone reserves. Adult zebrafish, however, possess fully functional thyroid glands. Disruption of these maternal thyroid hormone reserves during embryo-larval development, as well as EDC-induced alterations in thyroid histology and hormone levels in adults are likely associated with oxidative stress. Deregulation of thyroid hormones in zebrafish, whether caused by developmental thyroid deficiency or genetic defects, can lead to multiple neurodevelopmental impairments, including disrupted myelination, and altered neurotransmitter levels, ultimately resulting in movement-related neurobehavioral disorders. In binary exposure experiments with metals, the effects of EDCs were more complex than those observed in single exposure studies. Furthermore, the transgenerational transfer of the EDC-induced effects demonstrated significant contributions to neurodegeneration, highlighting the potential threat to human health. In addition, EDCs severely impact zebrafish by disrupting sex steroid hormones, leading to significant reproductive dysfunction, developmental abnormalities, and altered behavior in both embryos and adults. Despite the insights provided by these limited studies, substantial knowledge gaps remain regarding the mechanisms by which EDCs affect nervous system development, including epigenetic regulation, neurobehavior outcomes, and neuronal transmission.

## Data Availability

The datasets presented in this study can be found in online repositories. The names of the repository/repositories and accession number(s) can be found in the article/**Supplementary Material**.
