# Supplementary material for: A systematic review on the evaluation of endocrine-disrupting chemicals as potential neurotoxins during zebrafish development
Source: Front Endocrinol (Lausanne). 2026 Apr 23;17:1741250. doi: 10.3389/fendo.2026.1741250 (PMC13149087; doi:10.3389/fendo.2026.1741250)
Supplement: Supplementary file 1 [file Table1.docx]

**A systematic review on the evaluation of endocrine-disrupting chemicals as potential neurotoxins during zebrafish development.**

Asok K Dasmahapatra^1^, Chayan Dutta^2^, Anitha Myla^3^, Paul B Tchounwou^4, *^

^1^Department of BioMolecular Science, Environmental Toxicology Division, University of Mississippi, University, Oxford, MS 38677

^2^Department of Biology, Experimental Biophysics, Humboldt University, Berlin, Germany

^3^Mississippi State Department of Health, 570 East Woodrow Wilson Drive, Jackson, MS 39216

^4^ RCMI Center for Urban Health Disparities Research and Innovation, School of Computer, Mathematical and Natural Sciences, Morgan State University, Baltimore, MD 21251

Abstract

Endocrine-disrupting chemicals (EDCs) are well known for their ability to interfere with endocrine function, however, growing evidence indicates that they can also cause profound neurotic effects. These substances affect both the central and peripheral nervous systems of aquatic organisms, including fish, and can disrupt neurobehavioral development, posing a potential threat to aquatic species. In this systematic review, we examine the neurotoxic effects of EDCs on zebrafish (*Danio rerio*) , a widely utilized model organism for investigating vertebrate nervous system development. A comprehensive bibliographic search was conducted in PubMed using the terms “developmental neurotoxicity” and “fish,” – which yielded 603 articles. The search was refined by incorporating the terms “endocrine disruptors” and “zebrafish,” resulting in 15 relevant studies. Of these, three were excluded—two due to irrelevance to the topic, and one for being a review on strobilurin, a fungicide. The remaining 12 studies provided detailed insights into the neurotoxic effects of 14 chemicals, including 6-OH-BDE-47, Atrazine, BDE-209, Bisphenol (BPA), bisphenol S (BPS), cadmium (Cd), estrone, OBS, lead (Pb), tributyltin (TBT), tris(2-chloroethyl) phosphate (TCEP), tris(1,3-dichloro-2-propyl) phosphate (TDCPP), THM, and titanium (Ti). These compounds, encompassing fungicides, herbicides, heavy metals, hormones, and flame retardants, were identified as potential EDCs that disrupt nervous system development and behavior in zebrafish during embryonic, larval, and adult stages. Several studies have also investigated co-exposure effects of binary mixtures such as BDE 209 with Pb, BPA with Ti, and TBT with Cd. We identified several significant neurodevelopmental endpoints, including alterations in hormone levels (T3 and T4), neurotransmitter contents (dopamine, serotonin, acetylcholine, GABA, histamine, norepinephrine), acetylcholinesterase (AChE) activity, locomotor behavior, and expression of sensitive genes and proteins. Notably, these neurotoxic effects were shown to have intergenerational and transgenerational consequences. Overall, this review provides comprehensive evidence of the neurotoxic potential of EDCs on zebrafish development, emphasizing their relevance to vertebrate neurodevelopment and the potential implications for human health.

Supplementary Table 1

| Developmental stage | Chemicals | Concentration, and mode of exposure | Duration of exposure | Evaluation | Effects | References |
| --- | --- | --- | --- | --- | --- | --- |
| Embryos (AB strain) | 6-OH-BDE-47  [mBDE-47] | 1, 10, 50, and 100 nM; waterborne | 4-96 hpf | 22, 26, 30, 34, and 96 hpf | 1. Coiling frequency (behavior) in control embryos was gradually reduced as the development proceeds. 6-OH-BDE-47 exposure significantly delayed the reduction of coiling frequency of the zebrafish embryos in a time and concentration-dependent manner 2. Injection of thyroid hormone receptor β mRNA ( 3 nl of 265 μg/μL) to the embryos during 4 hpf, partially rescued the embryos from the toxic potential of 6-OH-BDE-47 as evidenced by coiling behavior evaluated during 26 hpf of development. 3. The number of apoptotic neurons in the brain of the embryos (26 hpf) were significantly enhanced in the embryos exposed to 6-OH-BDE-47. Injection of THRβ mRNA rescued this effect. 4. The number of serotonin (5-HT) immunoreactive neurons in the hypothalamus of the brain of the embryos (96 hpf larvae) significantly reduced after 6-OH-BDE-47 exposure. 5. The expression of *tph2* mRNA was significantly reduced in a concentration-dependent manner in the embryos (26 hpf) exposed to 6-OH-BDE-47 (only in embryos exposed to 100 nm 6-OH-BDE-47). | Wang et al., (2018) |
| Embryos (AB strain) | Atrazine (Herbicide) | 0.3, 3, and 30 μg/L; 1-72 hpf, waterborne | 9,12, and 14 months | Adults (9, 12, and 14 months of age) | 1. At 9 months post fertilization males had decreased locomotor parameters. 2. Transcriptomic analysis identified altered gene expression in organismal development, cancer, and nervous and reproductive system development 3. Histopathological analysis of the brain identified morphometric differences and reduced number of cells in the raphe populations. 4. No significant difference was observed in the body weight, brain size, and weight of the fish, at 14 months of age. 5. The global genome methylation pattern remained unaltered as observed at 12 months of age. | Horzmann et al., (2022) |
| Embryos (AB strain) | Atrazine (Herbicide) | 0.3, 3, and 30 μg/L; 1-72 hpf, waterborne | 9 months | Adults (9 months of age) | 1. The number of differentially methylated genes (DMG) in the brain of female fish increased with increasing treatment concentrations 2. Hypomethylation in estrogen receptor signaling 3. Hypermethylation in androgen signaling 4. DMGs with methylation changes in gene body were primarily enriched for mitochondria-related pathways | Wang et al., (2022) |
| Embryos | Bisphenol A | 1, 10, 100, 1,000, and 10,000 nM of BPA | 0-5 dpf and 4-5 dpf | Larvae 5 dpf | 1. BPA exposure (0-5 dpf) induced significant concentration-dependent skeletal (10,000 nM) and total abnormalities (100 and 10,000 nM) when compared with controls. 2. Other abnormalities, such as uninflated swim bladder, cardiac edema, yolk sac edema did not show any significant difference when exposed 0-5 dpf of development. 3. Short term exposure (4-5 days) did not show significant difference with regard to skeletal and total abnormalities, swim bladder inflation, cardiac edema, and yolk sac edema. 4. In behavioral analysis, embryos exposed to BPA (0-5 dpf) showed concentration-dependent hyperactivity in the dark (100 and 10,000 nM); however, short-term exposure (4-5 dpf) induced hypoactivity (1, 10, 100, and 10,000 nM) in a concentration-dependent manner 5. A total of 168 DEGs were expressed in embryos (0-5 dpf) when exposed to BPA, while 1296 DEGs were observed when the embryos (4 dpf) were exposed to BPA for 1 day(4-5 dpf) only. 6. 90 DFGs were common between the larvae either exposed to BPA (0-5 dpf) or larvae (4-5 dpf). 7. Five DEGs (*pck1, pnp4b, psmb5, dnajc5gh*, and *zgc:92590*) are common to the larvae exposed to estrone | Wu et al., (2021) |
| Embryos (Tübingen strain) | BPS | 1, 10, and 100 μg/L | 2hpf- 120 dpf | F0 Adults (120 dpf), F1 embryos and larvae. | A; Adults (F0)   1. In females, plasma levels of T4 were significantly decreased in all concentrations of BPS used in this study; in males, the plasma T4 levels remained unaltered. 2. In females, plasma T3 levels were significantly increased in fish exposed to all concentrations of BPS, however, in males significant increase was observed in lower concentrations (1 and 10 μg/L) but not in highest concentration (100 μg/L) used in this study.   B: F1 eggs:   1. Hatching was significantly delayed in embryos obtained from the parents exposed to BPS (1, 10, and 100 μg/L). 2. The head-trunk angles (HTA) were significantly reduced and the length of otic vesicle significantly increased in embryos (30 hpf) obtained from the parents exposed to BPS (1, 10, and 100 μg/L) for 120 days. 3. Compared with controls, the spontaneous movements (touch-evoked response) during 48 hpf was significantly reduced in embryos derived from the parents exposed to BPS (1, 10, and 100 μg/L) for 120 days. 4. The expression of *zhe1* gene (encoding zebrafish hatching enzyme) was significantly reduced in F1 eggs derived from the parental exposure to BPS for 120 days 5. As in parents, compared with controls, T4 concentration was significantly reduced in eggs derived from the parents exposed to BPS (1, 10, and 100 μg/L) for 120 days. 6. The T3 concentrations in the eggs derived from the F0 parents exposed to BPS (1, 10, and 100 μg/L) for 120 days were increased significantly when compared with controls.   C: larvae (F1)   1. Compared with controls, swim bladder inflation was significantly decreased in F1 larvae derived from the parents exposed to BPS (1, 10, 100 μg/L) for 120 days. 2. Compared with controls, the number (concentration-dependent) and area (in all concentrations) of lateral stipe melanocytes were significantly reduced in larvae derived from the parents exposed to BPS (1, 10, and 100 μg/L) for 120 days 3. Compared with controls, the average swimming speed and the swirl-escape rate was significantly reduced in F1 larvae (96 hpf) obtained from the parents exposed to BPS (1, 10, 100 μg/L) for 120 days. 4. Compared with controls, a concentration-dependent significant reduction in the expression of surfactant protein genes (*sp-a, sp-b*, and *sp-c*) was observed in larvae (96 hpf) obtained from the parents exposed to BPS (1,10, and 100 μg/L) for 120 days. 5. Compared with controls, a concentration-dependent significant upregulation in the expression of *gfap, gap43*, and *mbp* and downregulation of *syn2a* mRNA was observed in larvae (96 hpf) obtained from the parents exposed to BPS (1,10, and 100 μg/L) for 120 days. 6. Compared with controls, a concentration-dependent significant reduction in the expression of *crestin* and *tyr* was observed in larvae (96 hpf) obtained from the parents exposed to BPS (1,10, and 100 μg/L) for 120 days. | Wei et al., (2018) |
| Embryos | Estrone (E1) | 0.01, 0.1,10 and 100 nM estrone; | 0-5 dpf and 4-5 dpf | Larvae 5 dpf | 1. Embryonic exposure to estrone (0-5 dpf) led to concentration-dependent skeletal abnormalities and uninflated swim bladder and the calculated total abnormalities are not significantly different from controls. 2. The larvae (5 dpf) treated with estrone during development (0-5 dpf or 24 h only 4-5 dpf) become hypoactive in dark which is concentration dependent. 3. A total of 445 differentially expressed genes (DEG) were observed after estrone exposure during embryonic development (0-5 dpf); however, a total of 83 DEGs were observed when the embryos (4 dpf) were exposed to estrone for 24 h. 4. Five DEGs (pck*1, pnp4b, psmb5, dnajc5gh*, and *zgc:92590*) are common to the embryos exposed to BPA | Wu et al., (2021) |
| Embryos | Sodium *p*-perfluorous nonenoxybenzene sulfonate (OBS) | 3, 30, and 300 μg/L | 2 hpf- 21 days | Evaluated on F0 adults (180 dpf), F1  eggs (2 hpf) and F1 larvae (7 dpf) | F0 adults:   1. OBS exposure did not significantly affect the average colloid area in thyroid 2. The thickening of follicular epithelia and depletion of colloid in the lumen of thyroid follicles was observed in fish exposed to OBS 3. The height of epithelial cells in the thyroid gland of both male and female zebrafish is significantly increased after OBS exposure during early life stages 4. T3 levels in plasma of both male and female fish showed non-monotonic enhancements when exposed to lower concentrations of OBS during early life stages (3 μg/L in females and 3 and 30 μg/L in males). 5. T4 levels in females showed significant concentration-dependent decrease and in males significant concentration-dependent increase was observed when OBS exposure was made during early life stages. 6. The expression of *crh* gene was enhanced in a concentration-dependent manner (300 μg/L), and the expression of *tshβ* tended to decrease (not significant) in female fish brain exposed to OBS during early development. 7. In male brain, the transcription of *crh* and *tshβ* remained unaltered when the fish were exposed to OBS during early developmental stages. However, expression of tshr was significantly downregulated in only in lower dose of OBS ( 3 μg/L) 8. The expression of *tpo* was upregulated in female brain in a nonmonotonic fashion, while in males, both *tg* and *tpo* were downregulated in a concentration-dependent manner when the fish were exposed to OBS during early development. 9. The expression of *trα* was significantly downregulated in male brains and *trβ* was upregulated in female brains when the fish were exposed to OBS during early developmental stages. 10. The expression of *ttr* was significantly upregulated in the liver of female fish in a concentration-dependent manner, while in males no effects on *ttr* of liver was observed when the fish were exposed to OBS during early developmental stages. 11. The expression of *dio2* in the liver of female fish and *dio1* and *dio2* in male fish was downregulated in a concentration-dependent manner when the fish were exposed to OBS during early stages of development. 12. The expression of *ugt1ab* in liver of female fish was downregulated while in male fish was upregulated by OBS exposure during early stages of development.   B: Eggs (F1).   1. The T3 contents in F1 eggs showed significant increase while T4 levels remained unaltered when the parents were exposed to OBS during early life stages 2. During embryo-larval developments, the rates of hatching, malformation, survival, and the lengths and weight of the hatched embryos (7 dpf) did not show any significant difference after the parental exposure to OBS during early life stages.   C: Larvae (F1, 7 dpf)   1. The swirl-escape rate in the F1 larvae (7 dpf) significantly decreased in a concentration-dependent manner when the parents were exposed to OBS in a concentration-dependent manner. 2. Compared with controls, the T3 content in the whole larvae (7 dpf) was significantly decreased in lower concentration (3 increased in higher concentrations OBS groups (30 and 300 μg/L) while T4 content decreased significantly due to parental exposure in OBS during early stages of develoment 3. Gene expression analysis indicates that parental exposure to OBS during early life stages, significantly decreased the expression of *tshβ*, *tg*, and *nkx2.1* 4. The expression of *syn2a* was upregulated when the parents were exposed to lower concentration of OBS (3 μg/L) and the expression of *mbp* was upregulated when the parents were exposed to OBS at higher concentrations (300 μg/L). | Zhao et al., (2024) |
| Embryos (TU strain) | Tris (2-chloroethyl) phosphate) [TCEP] | 0.2,2,20, and 200 μg/L | 120 hpf | Evaluated on 48, 72, 96, and 120 hpf. | 1. Significant development-specific reduction in heartbeats was observed in embryos (200μg/L; 48 hpf) exposed to TCEP during development. 2. The length of the embryos reduced in a concentration-dependent manner (20-200 μg/L) in 72 and 120 hpf of development. 3. A concentration-dependent inhibition (not significant) in hatching rates of the embryos (20 and 200 μg/L) was observed only during 72 hpf. 4. Did not affect the malformation rates of the larvae (yolk sac edema, tail deformation, bent spine) 5. T3 levels of larvae (120 hpf) did not show any significant difference with controls, however, T4 levels significantly reduced after TCEP exposure in a concentration-dependent manner 6. Gene expression analysis in 120 hpf larvae indicated that expression of *nis* and *trα* were significantly upregulated in embryos exposed to 2, 20 and 200 μg/L of TCEP 7. The expression of *trβ,* and *ugt1ab* was upregulated (2 and 20 μg/L), while the expression of *tg* and *tshr* was downregulated in larvae developmentally exposed to TCEP (0.2-200 μg/L) 8. The expression of *α1-tubulin* were downregulated whereas *gap43* and *mbp* were significantly upregulated in larvae (120 hpf) after exposure with TCEP (0.2, 2, 20 and 200 μg/L) 9. Upregulation of *syn2a* mRNA was observed only in larvae exposed only to 200 μg/L (concentration-dependent) during development 10. The expression of *elavl3* was enhanced only in larvae exposed to 20 μg/L, however, downregulated in larvae exposed to 200 μg/L during development. | Hu et al., (2021) |
| Embryos | Thifluzamide (THM) | 0.19, 1.90, 2.85 mg/L | 4-6 days | Evaluated on larvae 6 dpf | 1. Reduced hatching 2. Induced spinal curvature in larvae 3. Induced pericardial edema in embryos (72 hpf) 4. Larval length significantly decreased (96 h onwards) 5. The distance moved, the average velocity, and the larval activity was significantly inhibited by THM exposure 6. The neurotransmitters, serotonin (5-HT) and norepinephrine significantly increased in larvae exposed to THM in a concentration-dependent manner. 7. The AChE activity significantly decreased in the larvae exposed to THM in a concentration-dependent manner. 8. The expressions of *flk1*, *neurog1*, and *vegf* were upregulated by THM in a concentration-dependent manner while the expression of *idha*, *aidh2.1* were significantly downregulated in larvae exposed to THM. 9. The thyroid releasing hormone (TRH) and thyroid stimulating hormones (TSH) did not show significant changes in zebrafish larvae after THM exposure 10. The T3 and T4 contents in the larval body (96 hpf) were significantly reduced in a concentration-dependent manner by THM exposure 11. The expression of HPT axis genes showed significant changes. 12. The expressions of *pax8, tshγ,tshβ,ahr2, cyp1a, thrα,thrβ, hhex*,and *ugt1ab* were upregulated after THM exposure and the enhancement is positively correlated with the concentration of THM used in this study. 13. The expression of *dio1, dio2, nkx2.1, tg, crh*, and *ttr* by THM are not significantly correlated with the concentrations of THM used in this study. | Yang et al., (2021) |
| Adults ( 4 months age) | BDE-209 and Lead acetate trihydrate. | BDE-209 (1, 10, and 100 μg/L) and Pb (10 μg/L) | 3 months | Evaluated in F0 adults, F1 eggs, and in larvae (5 dpf) | A: F0 adults   1. In adult males exposure to BDE-209 did not change the length and weight of the fish, however, the condition factor [(weight/Length ^3^)X100] was inconsistently increased in fish exposed to 1 and 100 μg/L BDE-209. 2. Coexposure of male fish with BDE-09 and Pb, significantly decreased the body weight in fish exposed to 1μg/L BDE 209+Pb and increased both length and weight of the fish exposed to 100 μg/L BDE-209+ Pb; however, the condition factor was found to be significantly decreased only in fish exposed to 1 μg/L BDE-209+Pb. 3. Fish exposed only to Pb did not show any significant change in the length and weight of the male and female fish, however, the condition factor in males, significantly decreased when compared with controls. 4. In females, exposure to BDE-209 did not show any significant change in the length and weight of the fish, except the length of the fish exposed to 1μg/L BDE-209, where the length showed significant increase when compared with controls, though the condition factor did not show any significant change with control fish when exposed to BDE-209 (1, 10, and 100 μg/L) either alone or in combination with Pb (10 μg/L). 5. The hepatosomatic index [ ( liver weight/Body weight)X100) in male fish remained unaltered by BDE-209 (1, 10, 100 μg/L) either alone or in coexposure with Pb (10 μg/L). however, in females, HSI significantly decreased in fish exposed to 1 μg/L BDE-209+Pb and increased in fish exposed to 100 μg/L+ Pb. 6. The brain somatic index [BSI= (weight of brain/body weight)X100] did not show any significant change in male fish exposed to all concentrations of BDE-209 either alone or in combinations with Pb; however, females exposed to BDE-209 (100 μg/L) showed significant increase when compared with controls and coexposure with Pb showed significant decrease when compared with the fish exposed to 100 μg/L BDE-209 alone. 7. The gonadosomatic index ( [GSI=( gonad weight/bodyweight)X100]   significantly decreased in male fish exposed only to BDE-209 (10 μg/L) alone; coexposure with Pb significantly decreased GSI when compared with BDE-209 (10 and 100 μg/L) alone.   1. In females, GSI remained unaltered when exposed to BDE-209 either alone or in combinations with Pb; however, significant increase in GSI was observed when the females were exposed to BDE-209 (10 μg/L)+ Pb. 2. No significant effect was observed on fecundity; however, significant reduction was observed when the parents were exposed to 100 μg/L of BDE-209 + Pb. 3. Significant concentration-dependent increase in serum T3 level was observed in male fish exposed to BDE-209 (100 μg/L) either alone or in combination with Pb (10 μg/L) 4. No significant alteration in the serum T4 levels of male fish exposed to BDE-209 alone or in combination with P; however, BDE-209 at 10 μg/L significantly increased serum T4 level when coexposed with Pb (10 μg/L) 5. In females, compared with controls, serum T3 level significantly decreased when exposed with BDE-209 alone; coexposure with lead 10 μg/L) significantly decreased serum T3 levels in females when BDE-209 concentrations are 1 and 10 μg/L. 6. Serum T4 contents in females remained unaltered in fish exposed to lower concentrations of BDE-209 (1 and 10 μg/L) and significantly decreased in females exposed to BDE-209 (100 μg/L). presence of lead (10 μg/L) either alone or in combinations with BDE-209 ( 1, and 100 μg/L) significantly decreased serum T4 levels when compared with controls. 7. The serum testosterone (T) levels in males remained unaltered by BDF-209 (1, 10, 100 μg/L) or Pb(10 μg/L) exposure. However, coexposure of BDE-209 with lead significantly reduced serum T levels in male fish. 8. In females both BDE-209 (1, 10, 100 μg/L) and Pb (10 μg/L) either alone or in combinations was able to significantly reduce serum T levels when compared with control fish. 9. The serum E2 levels in male fish remained unalter when the fish were exposed to lower concentrations of BDE-209 (1, and 10 μg/L) or lead (10μg/L) alone, while higher concentration of BDE-209 alone (100 μg/L) significantly enhanced serum E2 levels in male fish. Moreover, combined exposure of Pb (10 μg/L) with all concentrations of BDE-209 (1, 10, 100 μg/L) serum E2 levels in male fish reduce significantly when compared with the fish exposed to corresponding BDE-209 alone. 10. In females, serum E2 levels significantly decreased by BDE-209 (10 and 100 μg/L) and Pb (10 μg/L) alone. Moreover, significant reduction in serum E2 levels were also observed in fish (females) in combined exposure conditions (BDE-209+Pb)     B: F1 eggs   1. Significant concentration-dependent accumulation of PBDE (total) was found in F1 eggs derived from the parental exposure of BDE-209, and the presence of Pb in the environment further increased the accumulation of PBDE in the eggs. 2. Accumulation of Pb in the eggs was increased in a concentration-dependent manner when the parents were coexposed with BDE-209 (1, 10, and 100), however, significant increase was observed only in parents exposed to 100 μg/L of BDE-209+Pb 3. The size of the eggs significantly increased when the parents were exposed only to BDE-209 (1 μg/L)+ Pb. 4. BDE-209 alone has no significant effect on hatching of the embryos, while Pb either alone (10 μg/L) or in presence of BDE-209 (10 and 100 μg/L) significantly delayed hatching. 5. T3 content significantly decreased in eggs (F1) derived from parents exposed to 1μg/L BDE-209 and remained unaltered in eggs derived from fish exposed to higher concentrations of BDE-209 (10 and 100 μg/L); T3 content in eggs derived from fish exposed to BDE-209 (10 and 100 μg/L) and Pb (10 μg/L) significantly reduced when compared with control eggs (F1). 6. T4 contents in eggs (F1) derived from parents exposed to BDE-209 (1, 10, and 100 μg/L) alone remained unaltered, while T4 contents significantly reduced in eggs derived from the fish coexposed fish with Pb (10 μg/L) and BDE-209 (10 and 100 μg/L). 7. Concentration of T significantly reduced in eggs derived from parents exposed to BDE-209 (1, 10, 100 μg/L) alone; Pb (10 μg/L) alone was able to significantly reduce T contents when compared with controls. Eggs derived from parents coexposed with BDE-209 (1 and 10 μg/L) and Pb (10 μg/L) showed significant reduction, while significant enhancement was observed in the T content of eggs derived from parents exposed to BDE-209 (100 μg/L) in combination with Pb (10 μg/L). 8. E2 content was enhanced in eggs derived from the parents exposed to 10 and 100 μg/L BDE-209 alone. Moreover, eggs derived from parents exposed to Pb either alone (10 μg/L) or in combinations (1, and 10 μg/L+ Pb) did not show any significant effect. However, significant enhancement in the E2 content was observed in eggs derived from the fish exposed to BDE 209 (100 μg/L) and Pb (10 μg/L) in combinations.   C: F1 larvae (5 dpf)   1. The length and weight of the larvae remained unaltered when the parents were exposed to BDE-209 (1, 10, and 100 μg/L) and lead (10 μg/L) either alone or in combination. 2. The accumulation of PBDE (total) was found to be concentration-dependent in larvae derived from the parents exposed to BDE-209 alone. Coexposure with Pb (10 μg/L) significantly increased the accumulation of PBDE in the larvae derived from the parents exposed to BDE-209 (10 μg/L+ Pb) and 100 μg/L+ Pb). 3. Although accumulation of Pb was decreased in larvae coexposed with BDE-209 and PB, it was significant only in larvae when the parents were coexposed only with BDE-209 (10μg/L+ Pb) for 4 months. 4. T3 levels of larvae derived from parents exposed to BDE-209 alone (1 and 10 μg/L) remained unaltered and significantly increased in larvae derived from parents exposed to BDE-209 (100 μg/L). T3 contents significantly increased in larvae derived from parents coexposed with lead (10 μg/L) and BDE-209 (10 and 100 μg/L). 5. T4 contents in larvae derived from parents exposed to BDE-209 (1, 10, 100 μg/L) alone remained unaltered; while significant increase in T4 contents was observed in larvae derived from parents coexposed with BDE-209 (1,10, 100 μg/L) and Pb (10μg/L). 6. Downregulation of the thyroid hormone receptor protein 3b was observed in larvae derived from parents exposed to BDE-209 (1μg/L) and Pb (10 μg/L) either alone or in combinations. 7. The T contents in larvae derived from the parents exposed either to BDE-209 (1, 10, and 100 μg/L) or Pb (10 μg) alone or in combination showed significant reduction when compared with controls. 8. The E2 content in larvae derived from the parents exposed to BDE-209 (10 and 100 μg/L) either alone or in combination with Pb (10 μg/L) significantly reduced when compared with controls. Moreover, the E2 content in larvae derived from parents exposed with Pb (10 μg/L) alone did not show any significant change, while significantly reduced in larvae when the parents were exposed in combinations (1 μg/L BDE-209+Pb). 9. Proteomic analysis of the larvae derived from parents exposed to BDE-209 (1 μg/L) either alone or in combination with Pb (10μg/L) ) did not induce any significant change in the vitellogenin content of the larvae, while Pb (10 μg/g) alone was able to show universal over expression of vitellogenin. Moreover, downregulation of cathepsin S by BDE-209 and Pb either alone or in combination was observed. 10. The average swimming speed in both light and dark phages was decreased significantly in larvae derived from parents exposed to BDE 209 (1, 10, and 100 μg/L) and Pb (10 μg/L) either alone or in combinations. 11. The neural protein syn2a was decreased significantly in a concentration-dependent manner in larvae derived from the parents exposed to BDE-209 (significant in 10 and 100 μg/L) and Pb (10 μg/L) either alone or in combinations. 12. The neural protein mbp was decreased significantly in a concentration-dependent manner in larvae derived from the parents exposed to BDE-209 (1, 10 and 100 μg/L) and Pb (10 μg/L) either alone or in combinations. 13. Proteomic analysis found increased and decreased levels of various crystalline proteins in larvae derived from parents exposed to BDE-209 (1μg/L) and Pb (10 μg/L) either alone or in combinations. 14. Proteomic analysis found increased and decreased levels of various muscle proteins in larvae derived from parents exposed to BDE-209 (1μg/L) and Pb (10 μg/L) either alone or in combinations. 15. Proteomic analysis found increased and decreased levels of various neuronal proteins such as internexin neuronal intermediate filament protein, alpha b, synaptic vesicle membrane protein VAT-1 homolog, syntaxin binding protein 1a, internexin neuronal intermediate filament protein alpha a, synaptotagmin binding, cytoplasmic RNA interacting protein, glial fibrillary acidic protein, myelin expression factor 2, Synaptotagmin binding, cytoplasmic RNA interacting protein, myelin expression factor 2, in larvae derived from parents exposed to BDE-209 (1μg/L) and Pb (10 μg/L) either alone or in combinations. 16. Various excitatory and inhibitory neurotransmitters, such as glutamate, GABA, serotonin, tryptophan, ACh, tyrosine, choline, 5-hydroxyindole acetic acid, dopamine, norepinephrine, epinephrine, 3,4-dihydroxy phenylacetic acid were altered in larvae derived from parents exposed to BDE-209 (1μg/L) and Pb (10 μg/L) either alone or in combinations. | Chen et al., (2017) |
| Adults (4 months of age) | BPA and Titanium dioxide (TiO_2_) | BPA (0.2 and 20 μg/L) + Titanium (100 μg/L) | 4 months | Evaluated in F0 adults (8 months of age), F1 eggs, and F1 larvae (5-10 dpf) | A: F0 adults   1. In single exposure experiments, concentration-dependent increase in the BPA and Titanium in the body of both males and females. In combined exposures (BPA and Titanium) the accumulation of both BPA and Titanium was found to be higher than the Titanium and BPA content of the fish exposed as single exposer. 2. In the brain of adult parents BPA upregulates the expression of *crh* and *tshβ* mRNAs. 3. In the brain of both male and female fish, the serotonin, dopamine and acetylcholine contents and the AChE activity remained unaltered after BPA exposure 4. Titanium is able to reduce serotonin and dopamine contents in the female fish. The acetylcholine content in female brain and the serotonin, dopamine, and acetylcholine content in male brain remained unaltered in adult F0 fish after titanium exposure. 5. In combined exposure, serotonin, and dopamine content in the female brain significantly reduced than controls as well as single exposure groups (20 μg/L BPA or 100 μg/L titanium); the acetylcholine content and the AChE activity in female brain, as well as melatonin, dopamine, acetylcholine contents and the AChE activity in male brain remained unaltered in F0 adults after 4 months of exposure to both BPA and titanium. 6. Plasma T4 level in females, not in males, were significantly decreased in a concentration dependent manner after BPA exposure, TiO2 is unable to induce any significant change in the serum T4 levels in F0 adult fish. However, combined exposure significantly reduce serum T4 levels in F0 adults ( both males and females) in a concentration-dependent manner. 7. T3 remained unaltered in both F0 adult male and female fish after exposure with BPA or titanium alone, while in combined exposure T3 levels in female fish significantly reduced in a concentration-dependent manner; in males, it remained unaltered.   B: F1 eggs   1. Concentration-dependent accumulation in the BPA and titanium in the F1 eggs was observed after the parents were exposed to the chemicals as single exposure; in combined exposures, the accumulation of BPA and titanium was significantly higher than the single exposure groups. 2. T4 contents in the F1 eggs significantly reduced when adults were exposed to BPA (2 and 20 μg/L) alone or in combination with titanium (20 μg/L BPA+ 100μg/L titanium) in a concentration-dependent manner; titanium alone was unable to induce any significant change in T4 contents. 3. T3 level remained unaltered in F1 eggs when parents were exposed to BPA (2 and 20 μg/L) or titanium ( 100 μg/L) for 4 months either alone or in combinations.   C: Larvae (F1; 5-10 dpf)   1. Hatching ( 3 dpf), malformation rates, and embryo-larval survivability, was significantly reduced in a concentration dependent manner, when the parents (F0) were exposed to (2-20 μg/L) BPA; titanium was unable to induce any hatching delay, malformations, or survivability; however, at combined exposure ( BPA 20 μg/L+ titanium 100 μg/L) significantly reduced hatching ( 3dpf), enhanced malformation rates, decreased survivability and body weight of the larvae. 2. Significant concentration-dependent reduction in the average swimming speed of the larvae in the light phase or during light dark transition phase, when the parents were exposed for 4 months with BPA (2-20 μg/L), or titanium (100 μg/L) alone, or in combinations 3. The neurotransmitters, serotonin and dopamine significantly reduced in a concentration-dependent manner (BPA), while no effect was observed in the acetylcholine contents of larvae, when the parents were exposed to BPA (2-20 μg/L) and titanium (100 μg/L) either alone or in combinations. 4. The AChE activity remained unaltered in larvae when the parents were exposed to BPA (2-20 μg/L), or titanium (100 μg/L) for 4 months. However, a significant reduction in the AChE activity was observed when the parents were exposed in a combined condition of BPA (20 μg/L) and titanium (100 μg/L) for 4 months. 5. The expression of Mbp, Syn2a, and α1 tubulin protein in the larvae (10 dpf) indicated that the Mbp and Syn2a (biomarkers for axon myelination and synapse formation) were reduced in larvae derived from the parents exposed to BPA (20 μg/L), and titanium either alone or in combinations for 4 months. However, α-tubulin (an intermediate filament protein, associated with the cytoskeletal organization of the developing neurons) was reduced in larvae derived from the parents exposed either to titanium alone or in combination with BPA (20 μg/l). 6. T4 content in the F1 larvae (10 dpf) decreased significantly in a concentration-dependent manner when parents were exposed for 4 months with BPA (2-20 μg/L) alone or in combinations with titanium. Titanium alone was unable to alter the T4 contents of the larvae (F1) when parents (F0) were exposed to TiO2 for 4 months. 7. The T3 level remained unaltered in all treatment groups, however, significantly reduced in larvae where parents were exposed to BPA (20 μg/L) and titanium (100 μg/L) together for 4 months. | Guo et al., (2019) |
| Zebrafish (Adults, 4 months old) (AB strain) | 1, 3-dichloro-2-propyl phosphate (TDCPP) | 4, 20, and 100 μg/L | 3 months | Evaluated in F0 adults (7 months of age), F1 eggs, and F1 larvae (5 and 10 dpf) | A: Adults (F0)   1. A concentration-dependent sex-specific accumulation (females appears to accumulate more than males) of TDCPP was observed (body burden) in adult zebrafish exposed for 3 months, Significant amount of BDCPP also detected in the body burden of both male and female fish. 2. Plasma T4 level in females was significantly reduced in a concentration-dependent manner (20-100 μg/L) 3. Plasma T3 levels in females significantly reduced in a concentration-dependent manner (100 μg/L). 4. In males TDCPP was unable to induce any significant change in the thyroid hormone levels (T3 and T4) in plasma of the fish.   B: Embryos (F1)   1. Depending on the concentration of exposure of the adult fish to TDCPP, significant amount of TDCPP was detected in the eggs of the fish (concentration-dependent). However, the metabolite BDCPP was found to be very minimum (undetectable -minimum) 2. The hatching of the embryos (3 dpf) was significantly reduced (only the eggs derived from the parents exposed to 20 and 100 μg/L TDCPP) 3. T4 levels was significantly reduced in eggs derived from the fish exposed to 100 μg/L TDCPP. 4. T3 content remained unaltered in the eggs obtained from the parents exposed to (4, 20 and 100 μg/L)   C: larvae (F1)- 5-and 10 dpf:   1. The growth of the larvae was inhibited in a concentration-dependent manner (only in larvae derived from the parents exposed to 100 μg/L TDCPP) 2. The malformation (spinal curvature) was increased, and survivability of the larvae ( 5 and 10 dpf) was decreased in a concentration-dependent ( parents exposed to 100 μg/L for 5 dpf, and 20 and 100 μg/L for 10 dpf) manner. 3. The swimming speed significantly reduced in the larvae ( 5 dpf) during light phase and light-dark transition phase, which were obtained from the parents exposed to 100 μg/L TDCPP for 3 months. 4. The average swimming speed in 10 dpf larvae derived from fish exposed to 100 μg/L TDCPP for 3 months, was reduced significantly than the larvae obtained from control fish. 5. The ROS content in the larvae (10 dpf) was found to be significantly increased when the parents were exposed to 100 μg/L TDCPP for 3 months. 6. The neurotransmitter content (serotonin, dopamine, GABA, and histamine) in 5 and 10 dpf larvae were also altered by TDCPP exposure to parents. 7. In 5 dpf larvae, histamine content remained unaltered, while serotonin content significantly decreased when the parents were exposed to highest concentration of TDCPP (100 μg/L) used in the study. GABA content was significantly reduced when the parents were exposed to 20 and 100 μg/L TDCPP for 3 months. 8. In 10 dpf larvae, serotonin and GABA was reduced significantly when the parents were exposed to the highest concentration of TDCPP (100 μg/L) used in this study. However, dopamine and histamine were reduced significantly in larvae (10 dpf) when the parents were exposed to 20 and 100 μg/L TDCPP for 3 months. 9. The mRNA expression pattern of *mbp* in 5 dpf larvae was significantly downregulated when the parents were exposed to 20 and 100 μg/L TDCPP and significant downregulation of *α1-tubulin* was also observed in 5 dpf larvae when the parents were exposed to only 100 μg/L TDCPP. The expression of *syn2a, gfap*, and *gap-43* remained unaltered in larvae (5 dpf) by parental exposure of TDCPP (4, 20, and 100 μg/L) for 3 months. 10. In 10 dpf larvae, the mRNA expression pattern of *mbp* and *syn2a* were significantly downregulated when the parents were exposed to 100 μg/L, while downregulation of *α1-tubulin* was observed in larvae (10 dpf) when the parents were exposed only to 100 μg/L TDCPP for 3 months; in contrast, the expression of *gap-43* was significantly upregulated in 100 μg/L group, while *gap* mRNA remained unaltered by all the concentrations of TDCPP used in this study. 11. Expression of mbp protein was significantly reduced in 5 dpf larvae, when the parents were exposed to 100 μg/L TDCPP for 3 months; while α1-tubulin and syn2a was also significantly reduced in larvae, when the parents were exposed to 20 and 100 μg/L TDCPP for 3 months. 12. The protein analysis in 10 dpf larvae indicated that the expressions of mbp, α1-tubulin, and syn2a were significantly reduced when the parents were exposed to 20 and 100 μg/L TDCPP for 3 months. 13. T4 levels was significantly reduced in 5dpf larvae derived from the fish exposed to 100 μg/L TDCPP. However, in 10 dpf larvae T4 content significantly reduced in eggs derived from the fish exposed to both 20 and 100 μg/L TDCPP 14. T3 content remained unaltered in the larvae (5 and 10 dpf) obtained from the parents exposed to (4, 20 and 100 μg/L) for 3 months. | Wang et al., (2015) |

| Zebrafish  (AB strain, 3 months old) | TBT and Cd | 100 ng TBT and 100 ng Cd | 3 months (F0); 5 months (F1)  7 days (F2) | Evaluated in F0 adults (6 months of age), F1 ( larvae 7 dph; adults 5 months of age) and F2 (7 dpf larvae) | F0:  1.The dopamine content in the brain of adult male and female fish did not show any significant alteration exposed to Cd, TBT either alone or in combinations.  2. The serotonin content in the brain of adult males did not show any significant changes in fish exposed to TBT and Cd either alone or in combinations; however, in females, serotonin levels in the brain significantly decreased in fish exposed only to the combination of TBT and Cd. Single exposure, either to CD or TBT did not induce any significant change.  3. The AChE activity in the brain of both male and female fish remained unaltered after exposure to Cd, TBT, either alone or in combinations.  4. In both male and female fish, Cd or TBT alone was unable to alter the plasma T3 and T4 contents, while in coexposure groups (Cd+ TBT), T3 and T4 levels in males remained unaltered, but in females TH levels (both T3 and T4) significantly reduced when compared with controls.  5. The expression of crh gene remained unaltered in the brain of male fish exposed to Cd or TBT either alone or in combinations (Cd+TBT), however, the expression of tshβ was upregulated only in fish exposed to combinations of Cd and TBT, not in fish exposed to Cd or TBT alone.  6. in female brain both *crh* and *tsh β* was upregulated when the fish is coexposed with Cd and TBT (Cd+TBT), however, remained unaltered in single exposure groups.  7. in liver of males, the expression of *dio1*, *dio2*, *ttr* and *tg* remained unaltered in fish exposed to Cd or TBT either alone or combinations, however, the expression of *ugaf1ab* was significantly upregulated only in coexposed group (Cd+TBT) not with Cd or TBT when exposed to these chemicals alone.  8. In liver of females, the expression of dio1 and ttr did not show any significant changes when exposed to Cd or TBT either alone or in combinations, however, the expression of dio2 was upregulated and tg was downregulated only in fish exposed to Cd and TBT in combination (Cd+TBT); no significant effect was observed in the expression of dio2 and tg when the fish were exposed to these chemicals (Cd or TBT) alone.  9. The expression of ugat1ab in liver of females was upregulated by TBT either alone or in combination with Cd; however, Cd alone was unable to induce any significant effect on the expression of ugat1ab in the liver of females.  F1:   1. No significant alterations in survival rates and hatching of the embryos exposed to TBT or Cd, either alone or in combinations 2. Significant decrease in heart rates were observed when the fish exposed to a combination of TBT+ Cd; single exposure either to TBT or Cd alone did not induce any significant change. 3. Significant decrease in the larval length (7 dpf) was observed in fish exposed to TBT either alone or in combination with Cd. Exposure to Cd alone did not induce any significant change in the length of the larvae (7 dpf) 4. The dopamine content in the F1 larvae (7 dpf), and brain of adult males decreased significantly in fish exposed to TBT and Cd in combinations. Exposure of fish either to TBT or Cd alone did not induce any significant change in the dopamine content of the larvae or in the brain of male fish. 5. In F1 adult females, TBT either alone or in combinations with Cd were able to significantly reduce the dopamine content in the brain of the fish. 6. The serotonin content in the whole larvae (7 dpf) and male brains significantly reduce when exposed to combination of TBT and Cd; exposure either to TBT or Cd alone did not induce any significant change 7. In females, the brain serotonin level, significantly decreased by TBT either alone or in combinations with Cd. Single exposure of Cd had no significant effect on the brain serotonin content of the female fish 8. The AChE activity in the larvae (7 dpf) significantly decreased in fish exposed to a combination of Cd and TBT; however, in brain of male fish TBT alone and in combination with Cd significantly decreased the AChE activity in the brain; in females, Cd either alone or in combination with TBT significantly decreased the brain AChE activity. 9. In F1 fish (both male and females), plasma T3 content remained unaltered when exposed to either TBT or Cd alone, however, coexposure (Cd+ TBT) significantly reduced serum T3 content in male and female fish. 10. Plasma T4 level in male fish remained unaltered in fish exposed to TBT or Cd alone, while coexposure (Cd+TBT) significantly reduced T4 levels in plasma of male fish when compared with controls. 11. Plasma T4 level in female fish significantly reduced in fish exposed to Cd or TBT either alone or in combinations when compared with control fish. 12. The T3 and T4 contents of the whole body of the larvae (7 dpf) remained unaltered when the fish were exposed to Cd or TBT alone; however, coexposure with TBT and Cd significantly reduced both T3 and T4 contents of the larvae when compared with the controls. 13. In males, the expression of crh and tshβ in the brain and the expression of dio1, dio2, ttr, ugat1ab, and did not show any significant alteration in fish exposed to Cd and TBT either alone or in combinations. 14. In females, the expression of crh in the brain showed significant downregulation when coexposed with Cd and TBT. However, these chemicals (Cd and TBT) were unable to induce any significant change in crh expression when the fish were exposed to these chemicals (Cd or TBT) alone. 15. The expression of tshβ in the brain of female fish was downregulated significantly when the fish were exposed to Cd, and TBT either alone or in combinations. 16. In the liver, the expression of dio1, dio2, and ttr did not show any significant alteration when exposed to Cd and TBT either alone or in combinations, however, the expression of tg was significantly downregulated by Cd and TB either alone or in combination and the expression of ugat1ab was downregulated only in fish exposed to combined mixture of Cd and TBT. These chemicals (Cd or TBT) alone has no significant effect on the expression of ugt1ab in the liver of female fish. 17. In F1 larvae (7 dpf) the expression of crh, dio2, ttr remained unaltered in fish exposed to Cd and TBT either alone or in combinations; the expression of tshβ was downregulated when the fish was exposed only to the mixture of Cd and TBT. Moreover, the expression of dio1, and ugt1ab was downregulated by TBT either alone or in combinations with Cd and the expression of tg was significantly downregulated in fish exposed to Cd and TBT either alone or in combinations.   F2:   1. Survivability during embryo-larval development was significantly decreased in fish exposed to TBT or Cd, either alone or in combinations 2. Hatching was significantly delayed in the embryos exposed to TBT and Cd in combination; however, not significantly differ in embryos exposed either to TBT or Cd alone. 3. Significant decreases in heart rates were observed when the fish were exposed either to TBT alone or in combination with Cd. However, single exposure to Cd did not induce any significant change in heart rates. 4. Significant reduction in the length of the larvae (7 dpf) was observed when exposed to Cd, and TBT, either alone or in combinations. 5. Dopamine content in the larval body (7 dpf) significantly decreased in fish exposed to Cd and TBT either alone or in combinations. 6. The serotonin content in the whole body of the larvae (7 dpf) showed significant reduction when the fish exposed to Cd, and TBT either alone or in combinations. 7. The AChE activity in the whole body of the larvae (7 dpf) significantly decreased in fish exposed to Cd and TBT either alone or in combinations (Cd+TBT) 8. Compared with controls, both T3 and T4 contents in the whole body of the larvae significantly reduced in fish exposed to Cd or TBT either alone or in combinations 9. The expression of crh, tshβ, dio2, ttr, ugat1ab and tg in the larvae (7 dpf) were significantly downregulated when the fish were exposed to Cd and TBT either alone or in combinations. Moreover, the expression of dio1 in the larval body (7 dpf) was significantly downregulated in fish exposed to TBT either alone or in combination with Cd. Single exposure with Cd did not show any significant change in the expression of dio1in larvae (F2, 7dpf). | Li and Li (2020) |
| --- | --- | --- | --- | --- | --- | --- |
